# Changes in the global burden of untreated dental caries from 1990 to 2019: A systematic analysis for the Global Burden of Disease study

**DOI:** 10.1016/j.heliyon.2022.e10714

**Published:** 2022-09-21

**Authors:** XiaoFeng Qin, Hao Zi, XiaoJuan Zeng

**Affiliations:** a College of Stomatology, Guangxi Medical University, Nanning 530021, China; b Center for Evidence-Based and Translational Medicine, Zhongnan Hospital of Wuhan University, Wuhan 430071, China

**Keywords:** Burden of disease, Untreated dental caries, Incidence, Prevalence, Years lived with disability

## Abstract

**Objective:**

To investigate the burden of untreated dental caries in 204 countries and territories over 30 years.

**Methods:**

Data of untreated dental caries from 1990 to 2019, including the incidence, prevalence, and years lived with disability, were extracted from the Global Burden of Disease 2019 database. Estimated annual percentage changes were calculated to assess the changes in the age-standardized incidence, prevalence, and years lived with disability rates.

**Results:**

Globally, in 2019, there were 3.09 billion (95% uncertainty interval [UI]: 2.76–3.39 billion) new cases of untreated dental caries in permanent teeth (48.00% increase), 2.03 billion (1.77–2.33) prevalent cases (46.07% increase), and 2.00 million (0.93–3.88) YLDs (45.64% increase), all since 1990. From 1990 to 2019, the age-standardized incidence rate (ASIR) of untreated dental caries in permanent teeth showed an upward trend (estimated annual percentage changes [EAPC] = 0.01), but age-standardized prevalence rate (ASPR) (EAPC = −0.13) and age-standardized YLD rate (ASYR) (EAPC = −0.13) decreased. There were 1.15 billion (0.79–1.52) new cases of untreated dental caries in deciduous teeth (11.74% increase), 0.52 billion (0.41–0.63) prevalent cases (5.89% increase), and 0.20 million (0.09–0.43) YLDs (6.03% increase), all since 1990. From 1990 to 2019, the ASIR of untreated dental caries in permanent teeth showed a stable trend (EAPC = 0), but the ASPR (EAPC = −0.15) and ASYR (EAPC = −0.14) decreased. The incidence of untreated dental caries peaked at the ages of 5–9 and 20–24 years, and the prevalence and years lived with disability at 1–4, 20–24, and 60–64 years.

**Conclusion:**

Untreated dental caries remains a major global public health challenge, but demographic, sex, and regional differences in trends remain. Proactive intervention strategies, at both administrative and academic levels, based on dynamic changes, are needed.

## 1 Introduction

Untreated dental caries is a global public health issue and poses a serious economic burden. [1] Untreated caries can cause considerable pain and discomfort, and, if it spreads to the dental pulp, it can also cause infection, and ultimately sepsis and tooth loss, or even aggravate or induce systemic diseases. [2]

Untreated dental caries has obvious epidemiological characteristics and its prevalence is high. The Global Burden of Disease (GBD) 2017 study showed that the prevalence of dental caries in permanent teeth ranked first among 328 diseases. Approximately 2.3 billion people worldwide have caries in their permanent teeth, with years lived with disability (YLDs) amounting to 1.62 million, an increase of 9.4% compared with the incidence rate in 2007. [3]

In addition, dental caries is considered preventable and controllable. However, more scientific evidence will be needed to support the approaches for the prevention and control of dental caries.

The 2019 GBD, alongside its updated population-based dental caries registration data and methodological improvements, provides the most comprehensive estimate of the untreated dental caries burden. Seventy-seven new data sources have been added in the 2019 GBD. A total of 1696 citations were identified after deduplication, of which 147 were selected for full-text review, and 77 new sources were extracted from the following countries: Argentina (1), Brazil (47), Chile (5), Colombia (5), Cuba (5), Ecuador (1), El Salvador (1), Honduras (1), Mexico (5), Peru (5), and Venezuela (1).

However, data analysis had not been performed since 2010. Therefore, in this study, we describe the incidence, prevalence, and YLDs for untreated caries by using the framework of the Global Burden of Diseases Study (GBD) 2019. This is the first effort to study untreated caries at global, regional, and national levels for all ages, both sexes, and over time, from 1990 to 2019. The results of this study will help in the identification of the current burden of untreated caries and facilitate the development of global, regional, and national responses to support the prevention and treatment of untreated caries.

## 2 Materials and methods

### 2.1 Data source

The incidence, prevalence, and YLDs of untreated dental caries and their corresponding age-standardized rates (ASRs) in 204 countries and territories from 1990 to 2019 were obtained from the Global Health Data Exchange query tool (http://ghdx.healthdata.org/gbd-results-tool). The GBD study collected data on the incidence and prevalence of untreated dental caries through a systematic review following the Cochrane Handbook [4]. Details on the modeling strategy for caries data are available from Kassebaum et al. [5]. Many of the studies presented dmft or DMFT scores, which represent lifetime prevalence and were often described as “caries experience.” Lowercase letters (dmft) are used for deciduous dentition and uppercase letters (DMFT) for permanent dentition. “D” denotes decayed, “M” missing, “F” filled, and “T” teeth. The maximum dmft score is 20, and the maximum DMFT score is 32. To measure the burden of disability from dental caries, we considered only the data on current prevalence to be relevant and thus converted the lifetime prevalence data to current prevalence and incidence where possible. The following formula was used to convert lifetime prevalence to current prevalence: lifetime prevalence multiplied by the D/DMF ratio; the incidence was calculated from the DMFT increment. Both incidence and prevalence of untreated caries were considered in this study because their relative magnitude reflects the treatment rate of dental caries. High incidence and low prevalence suggest a high caries treatment rate [6].

Disability-adjusted life years are an important demographic indicator in GBD studies and are the sum of the years of life lost and YLDs [7]. Because deaths directly caused by oral diseases are uncommon, only YLDs were used in the present study. YLDs are calculated by multiplying frequency (prevalence), severity (disability weight), and duration of the condition [8]. The GBD 2019 study divided the world into 21 regions and 204 countries and territories according to geographic location.

The sociodemographic index (SDI) is a composite index of development status that is strongly correlated with health outcomes and was used to divide regions or countries into five levels (high, high-middle, middle, low-middle, and low). The SDI values and reference quintiles for all the GBD 2019 locations are available in the GBD datasets [9].

In addition, the age groups were divided into 17 subgroups based on an interval of 5 years, except for the 0‒1-year-old group. The methodological details were described in a previous GBD 2019 study [7, 9, 10].

### 2.2 Untreated dental caries definition

The present study included caries in permanent and deciduous teeth. In accordance with the WHO definition of untreated dental caries [11], the GBD study defined untreated caries as unfilled teeth with an unmistakable coronal cavity at the dentin level or a root cavity in the cementum that felt soft or leathery to probing. In the GBD database, untreated caries corresponded to the ICD-9 code 521.0 and the ICD-10 codes K02.3-K02.9.

### 2.3 Statistical analysis

The estimation process for untreated dental caries was introduced in the GBD 2019 publications [7, 9, 10]. A 95% uncertainty interval (UI) was reported for all estimates. The ASRs (per 100,000 population) were calculated as the sum of the products of age-specific rates (*a*_*i*_, where *i* denotes the ith age) and the number of individuals (or weight *w*_*i*_) in the same age group *i* of the selected reference standard population, divided by the sum of the standard population weights:

$$\text{ASRs}=\frac{\sum_{i=1}^{A}a_{i}w_{i}}{\sum_{i=1}^{A}w_{i}}\times 100,000$$

Estimated annual percentage changes (EAPCs) were used to describe the ASR trends within a specified time interval [12]. The EAPCs were estimated using a linear regression model: *y* = *α* + *βx* + ε, where *y* is ln(ASRs), *x* is the calendar year, and ε is the error term. The EAPCs were calculated as 100 × (exp(*β*) − 1), and the 95% confidence interval (CI) was obtained from the linear regression model [13]. When the estimated EAPCs and the lower limit of the 95% CI were both >0, the ASRs were considered to be increasing. Conversely, if the estimated EAPCs and the upper limit of the 95% CI were both <0, the ASRs were considered to be decreasing.

R software (version 4.0.5; https://www.r-project.org/) and Microsoft Excel (Version 2019; https://www.office.com/) were used for statistical analysis and visualization.

## 3 Results

### 3.1 Global burden and temporal trend in untreated dental caries

Globally, 3.09 billion (95% UI: 2.76–3.39 billion) incident cases of caries in permanent teeth were identified in 2019, with an estimated age-standardized incidence rate (ASIR) of 39200.36/100,000 (95% UI: 34905.21/100,000–43168.27/100,000) (Table 1). Caries in permanent teeth accounted for approximately 2.03 billion (95% UI: 1.77–2.33 billion) prevalent cases with an age-standardized prevalence rate (ASPR) of 25625.53/100,000 (95% UI: 22281.05/100,000–29372.01/100,000). YLDs for caries of permanent teeth amounted to nearly 2.00 million (95% UI: 0.93–3.88), with an age-standardized YLD rate (ASYR) of 25.26/100,000 (95% UI: 11.54/100,000–48.99/100,000). From 1990 to 2019, the ASIR of caries in permanent teeth showed an upward trend, but the age-standardized prevalence rate (ASPR) and the age-standardized YLD rate (ASYR) showed a downward trend.

**Table 1:** Incident cases, prevalent cases, and years lived with disabilities (YLDs) for caries in permanent teeth and deciduous teeth in 2019 for both sexes and the estimated annual percentage changes (EAPCs) between 1990 and 2019 by Global Burden of Disease region.

	Incidence (95% UI)				Prevalence (95% UI)				YLDs (95% UI)			
	permanent teeth		deciduous teeth		permanent teeth		deciduous teeth		permanent teeth		deciduous teeth	
	Change (%) **(1990–2019)**	**EAPC of ASIR (95% CI)**	Change (%) **(1990–2019)**	**EAPC of ASIR (95% CI)**	Change (%) **(1990–2019)**	**EAPC of ASPR (95% CI)**	Change (%) **(1990–2019)**	**EAPC of ASPR (95% CI)**	Change (%) **(1990–2019)**	**EAPC of ASYR (95% CI)**	Change (%) **(1990–2019)**	**EAPC of ASYR (95% CI)**
Global	48.00%	0.01 (0.01–0.0 2)	11.74%	0 (**−**0.02 to 0.03)	46.07%	**−**0.13 (**−**0.14 to **−**0.13)	5.89%	**−**0.15 (**−**0.18 to **−**0.12)	45.64%	**−**0.13 (**−**0.13 to **−**0.13)	6.03%	**−**0.14 (**−**0.17 to **−**0.12)
Male	46.70%	0 (**−**0.01 to 0.01)	12.19%	0.01 (**−**0.01 to 0.03)	45.60%	**−**0.13 (**−**0.13 to **−**0.13)	6.36%	**−**0.15 (**−**0.18 to **−**0.12)	45.22%	**−**0.12 (**−**0.13 to **−**0.12)	6.49%	**−**0.15 (**−**0.17 to **−**0.12)
Female	49.33%	0.03 (0.02–0.04)	11.26%	0 (**−**0.03 to 0.02)	46.52%	**−**0.14 (**−**0.14 to **−**0.13)	5.40%	**−**0.15 (**−**0.18 to **−**0.12)	46.04%	**−**0.13 (**−**0.13 to **−**0.13)	5.53%	**−**0.14 (**−**0.17 to **−**0.11)
Socio- demographic index												
High SDI	20.79%	**−**0.01 (**−**0.01 to 0)	2.45%	0.03 (**−**0.01 to 0.08)	19.53%	**−**0.19 (**−**0.22 to **−**0.15)	**−**10.87%	**−**0.26 (**−**0.37 to **−**0.15)	18.82%	**−**0.19 (**−**0.22 to **−**0.15)	**−**10.88%	**−**0.26 (**−**0.37 to **−**0.15)
High-middle SDI	25.38%	0.02 (0.01–0.03)	20.84%	0.03 (**−**0.03 to 0.1)	21.97%	**−**0.23 (**−**0.25 to **−**0.22)	**−**20.43%	**−**0.05 (**−**0.08 to **−**0.01)	21.46%	**−**0.23 (**−**0.24 to **−**0.21)	**−**20.32%	**−**0.04 (**−**0.08 to **−**0.01)
Middle SDI	37.43%	0.06 (0.01–0.11)	6.81%	0.12 (0.07–0.1 7)	43.81%	**−**0.1 (**−**0.1 to **−**0.09)	**−**8.16%	**−**0.04 (**−**0.06 to **−**0.02)	43.08%	**−**0.09 (**−**0.1 to **−**0.08)	**−**8.01%	**−**0.04 (**−**0.06 to **−**0.02)
Low-middle SDI	65.44%	0 (**−**0.01 to 0.01)	**−**9.27%	**−**0.1 (**−**0.12 to **−**0.07)	65.12%	**−**0.1 (**−**0.13 to **−**0.07)	6.98%	**−**0.21 (**−**0.27 to **−**0.15)	64.95%	**−**0.09 (**−**0.11 to **−**0.06)	7.28%	**−**0.2 (**−**0.26 to **−**0.14)
Low SDI	115.38%	0.04 (**−**0.01 to 0.09)	**−**45.57%	**−**0.06 (**−**0.1 to **−**0.01)	120.34%	**−**0.09 (**−**0.12 to **−**0.06)	84.03%	**−**0.13 (**−**0.15 to **−**0.12)	121.03%	**−**0.08 (**−**0.11 to **−**0.05)	84.56%	**−**0.12 (**−**0.14 to **−**0.11)
Region												
Andean Latin America	74.97%	0.1(0.08–0.13)	19.45%	0.07 (0.03–0.1 1)	71.34%	**−**0.24 (**−**0.31 to **−**0.17)	17.51%	**−**0.29 (**−**0.38 to **−**0.21)	70.86%	**−**0.24 (**−**0.3 to **−**0.17)	17.91%	**−**0.28 (**−**0.37 to **−**0.2)
Australasia	47.61%	**−**0.01 (**−**0.11 to 0.09)	16.51%	0.6 (0.04–1.16)	20.26%	**−**0.24 (**−**0.47 to **−**0.01)	12.05%	0.95 (0.55–1.36)	19.73%	**−**0.24 (**−**0.46 to **−**0.01)	12.04%	0.95(0.55–1.3 5)
Caribbean	35.98%	0.03 (0.01–0.0 4)	1.87%	**−**0.02 (**−**0.03 to **−**0.02)	35.04%	0.03 (**−**0.01 to 0.06)	0.03%	**−**0.04 (**−**0.05 to **−**0.03)	34.37%	0.02 (**−**0.01 to 0.06)	0.01%	**−**0.04 (**−**0.05 to **−**0.03)
Central Asia	41.76%	0.01 (**−**0.01 to 0.03)	9.58%	0 (0–0.01)	41.66%	**−**0.03 (**−**0.06 to 0)	6.71%	0.01 (0.01–0.0 2)	41.46%	**−**0.03 (**−**0.06 to 0)	6.94%	0.02 (0.01–0.0 3)
Central Europe	**−**4.35%	0.09 (0.08–0.1)	-35.52%	0.18 (0.15–0.2 2)	**−**10.69%	**−**0.17 (**−**0.2 to **−**0.13)	**−**41.91%	**−**0.22 (**−**0.24 to **−**0.19)	**−**11.17%	**−**0.16 (**−**0.2 to **−**0.12)	**−**41.88%	**−**0.21 (**−**0.24 to **−**0.19)
Central Latin America	53.09%	**−**0.12 (**−**0.14 to **−**0.1)	**−**2.39%	**−**0.08 (**−**0.12 to **−**0.05)	70.74%	0.18 (0.15–0.22)	**−**4.98%	0.07 (0–0.13)	69.75%	0.18 (0.15–0.2 2)	**−**4.85%	0.07 (0.01–0.1 4)
Central Sub- Saharan Africa	149.51%	0 (**−**0.01 to 0.02)	123.57%	0 (**−**0.02 to 0.01)	150.86%	0 (**−**0.04 to 0.04)	115.97%	0.04 (0.02–0.0 5)	152.01%	0.02 (**−**0.02 to 0.06)	115.94%	0.05 (0.03–0.06)
East Asia	17.60%	**−**0.04 (**−**0.07 to **−**0.02)	**−**25.95%	0.21 (0.15–0.2 8)	16.21%	**−**0.26 (**−**0.3 to **−**0.22)	**−**28.89%	0.09 (0.05–0.1 3)	15.44%	**−**0.25 (**−**0.3 to **−**0.21)	**−**28.78%	0.1 (0.06–0.14)
Eastern Europe	**−**6.78%	0.05 (0.03–0.0 8)	**−**27.18%	0 (**−**0.02 to 0.01)	**−**8.17%	**−**0.15 (**−**0.19 to **−**0.11)	**−**29.58%	**−**0.07 (**−**0.09 to **−**0.04)	**−**8.49%	**−**0.14 (**−**0.18 to **−**0.1)	**−**29.56%	**−**0.06 (**−**0.09 to **−**0.04)
Eastern Sub- Saharan Africa	132.85%	0.05 (0.04–0.0 5)	96.91%	**−**0.09 (**−**0.14 to **−**0.03)	116.45%	**−**0.25 (**−**0.28 to **−**0.23)	80.17%	**−**0.34 (**−**0.4 to **−**0.29)	117.18%	**−**0.24 (**−**0.26 to **−**0.21)	80.95%	**−**0.33 (**−**0.38 to **−**0.27)
High-income Asia Pacific	1.96%	**−**0.03 (**−**0.05 to **−**0.01)	**−**32.72%	**−**0.06 (**−**0.1 to **−**0.03)	6.27%	**−**0.16 (**−**0.19 to **−**0.13)	**−**36.48%	**−**0.8 (**−**1.02 to **−**0.58)	5.48%	**−**0.15 (**−**0.19 to **−**0.12)	**−**36.51%	**−**0.8 (**−**1.02 to **−**0.58)
High-income North America	26.62%	**−**0.03 (**−**0.06 to **−**0.01)	7.38%	0.07 (0.01–0.1 3)	24.85%	**−**0.12 (**−**0.19 to **−**0.05)	4.29%	0.07 (**−**0.19 to 0.32)	23.80%	**−**0.13 (**−**0.2 to **−**0.05)	4.23%	0.07 (**−**0.19 to 0.32)
North Africa and Middle East	91.76%	0.07 (0.06–0.0 9)	22.35%	0.03 (0.02–0.0 4)	89.68%	**−**0.1 (**−**0.14 to **−**0.07)	20.99%	**−**0.01 (**−**0.03 to 0.02)	89.02%	**−**0.1 (**−**0.14 to **−**0.06)	21.16%	0 (**−**0.03 to 0.02)
Oceania	110.40%	0.05 (0.04–0.0 6)	83.98%	0.03 (**−**0.02 to 0.08)	106.65%	**−**0.05 (**−**0.06 to **−**0.04)	86.68%	**−**0.04 (**−**0.11 to 0.02)	106.23%	**−**0.05 (**−**0.06 to **−**0.04)	86.40%	**−**0.04 (**−**0.1 to 0.02)
South Asia	78.69%	0.03 (0.02–0.0 4)	12.86%	**−**0.06 (**−**0.08 to **−**0.05)	76.41%	**−**0.08 (**−**0.14 to **−**0.03)	9.51%	**−**0.11 (**−**0.16 to **−**0.06)	76.27%	**−**0.07 (**−**0.12 to **−**0.02)	9.86%	**−**0.1 (**−**0.15 to **−**0.05)
Southeast Asia	51.94%	0.04 (0.03–0.0 5)	**−**2.28%	0.05 (0.03–0.0 8)	47.56%	**−**0.23 (**−**0.26 to **−**0.2)	**−**7.60%	**−**0.13 (**−**0.17 to **−**0.1)	47.21%	**−**0.22 (**−**0.25 to **−**0.19)	**−**7.34%	**−**0.12 (**−**0.15 to **−**0.09)
Southern Latin America	37.05%	0.01 (**−**0.06 to 0.08)	1.84%	**−**0.03 (**−**0.06 to 0)	44.56%	0.02 (**−**0.07 to 0.11)	1.47%	**−**0.05 (**−**0.18 to 0.08)	44.03%	0.02 (**−**0.07 to 0.12)	1.63%	**−**0.05 (**−**0.18 to 0.08)
Southern Sub- Saharan Africa	58.71%	0.05 (0.04–0.0 6)	25.17%	0.3 (0.23–0.37)	59.76%	**−**0.04 (**−**0.06 to **−**0.01)	27.85%	0.5 (0.37–0.62)	58.95%	**−**0.04 (**−**0.07 to **−**0.02)	28.09%	0.5(0.38–0.63)
Tropical Latin America	49.91%	**−**0.05 (**−**0.13 to 0.02)	**−**10.81%	**−**0.15 (**−**0.47 to 0.17)	53.25%	**−**0.22 (**−**0.33 to **−**0.11)	**−**12.48%	**−**0.04 (**−**0.33 to 0.26)	52.62%	**−**0.21 (**−**0.32 to **−**0.11)	**−**12.38%	**−**0.03 (**−**0.32 to 0.26)
Western Europe	10.50%	0.05 (0.04–0.05)	18.36%	0.21 (0.05–0.3 7)	8.38%	**−**0.27 (**−**0.32 to **−**0.23)	**−**13.63%	**−**0.65 (**−**0.74 to **−**0.56)	7.80%	**−**0.27 (**−**0.31 to **−**0.23)	**−**13.58%	**−**0.64 (**−**0.73 to **−**0.55)
Western Sub- Saharan Africa	149.99%	0.06 (0.05–0.07)	126.32%	**−**0.02 (**−**0.05 to 0)	144.24%	**−**0.08 (**−**0.09 to **−**0.06)	114.88%	**−**0.06 (**−**0.1 to **−**0.02)	144.96%	**−**0.07 (**−**0.08 to **−**0.05)	115.43%	**−**0.05 (**−**0.09 to **−**0.01)

EAPC estimated annual percentage change; ASIR age-standardized incidence rate; ASPR age-standardized prevalence rate; ASYR age-standardized YLDs rate; CI confidence interval. ∗All data reported as number or rate (95% uncertainty interval).

In 2019, 1.15 billion (95% UI: 0.79–1.52 billion) incident cases of caries of deciduous teeth were identified. Globally, there were approximately 0.52 billion (95% UI: 0.41–0.63 billion) prevalent cases of caries of deciduous teeth, with an ASPR of 7672.90/100,000 (95% UI: 6113.14/100,000–9339.58/100,000). YLDs for caries of deciduous teeth amounted to nearly 0.20 million (95% UI: 0.09–0.43), with an ASYR of 2.94/100,000 (95% UI: 1.28/100,000–6.27/100,000) (Table 1). From 1990 to 2019, the ASIR of caries in deciduous teeth showed a stable trend; however, the ASPR and ASYR showed a downward trend (Table 1).

### 3.2 Variation in untreated dental caries burden at regional and national level

**Figure 1:**
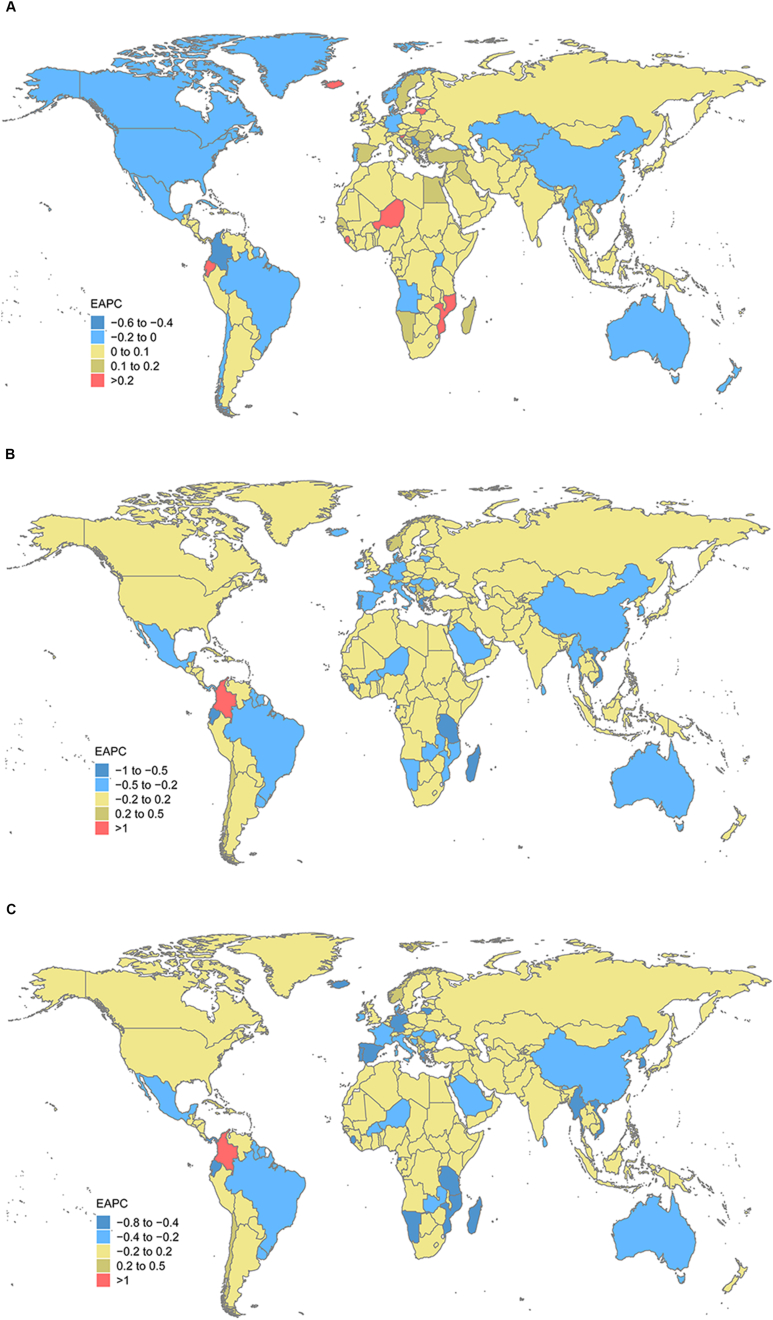
The Estimated Annual Percentage Changes (EAPCs) of untreated caries in permanent teeth Age-Standardized Rates Worldwide for (a) incidence (b) prevalence (c) YLDs. YLDs, years lived with disability.

The ASIR, ASPR, and ASYR of caries in permanent teeth during 1990–2019 varied across 21 regions and 204 countries and territories. The top three regions by age-standardized incidence rate of caries in permanent teeth in 2019 were South Asia, Southeast Asia, and Eastern Europe, and the top three countries and territories were Indonesia, Philippines, and India (appendix Table 1s). The three regions with the lowest age-standardized incidence rates were Southern Latin America, Andean Latin America, and Central Latin America, and the three countries and territories with the lowest rates were Romania, Chile, and Colombia (appendix Table 1s). In addition, the changes in the trends of the age-standardized rates of untreated caries in permanent teeth varied from country to country (Figure 1(a,b,c)). The top three regions by age-standardized prevalence rate of caries in permanent teeth in 2019 were East Asia, Oceania, and Eastern Europe, and the top three countries and territories were Taiwan (Province of China), Armenia, and Azerbaijan (appendix Table 1s). Over 120 countries and territories over the period 1990–2019 saw an upward trend in EAPC for ASIR, while ASPR and ASYR showed a downward trend, such as in Thailand, Argentina, and France. During 1990–2019, the EAPC of ASIR in 27 countries and regions showed a stable trend, while ASPR and ASYR showed a decreasing trend, such as in Brazil, Japan, and Canada.

**Figure 2:**
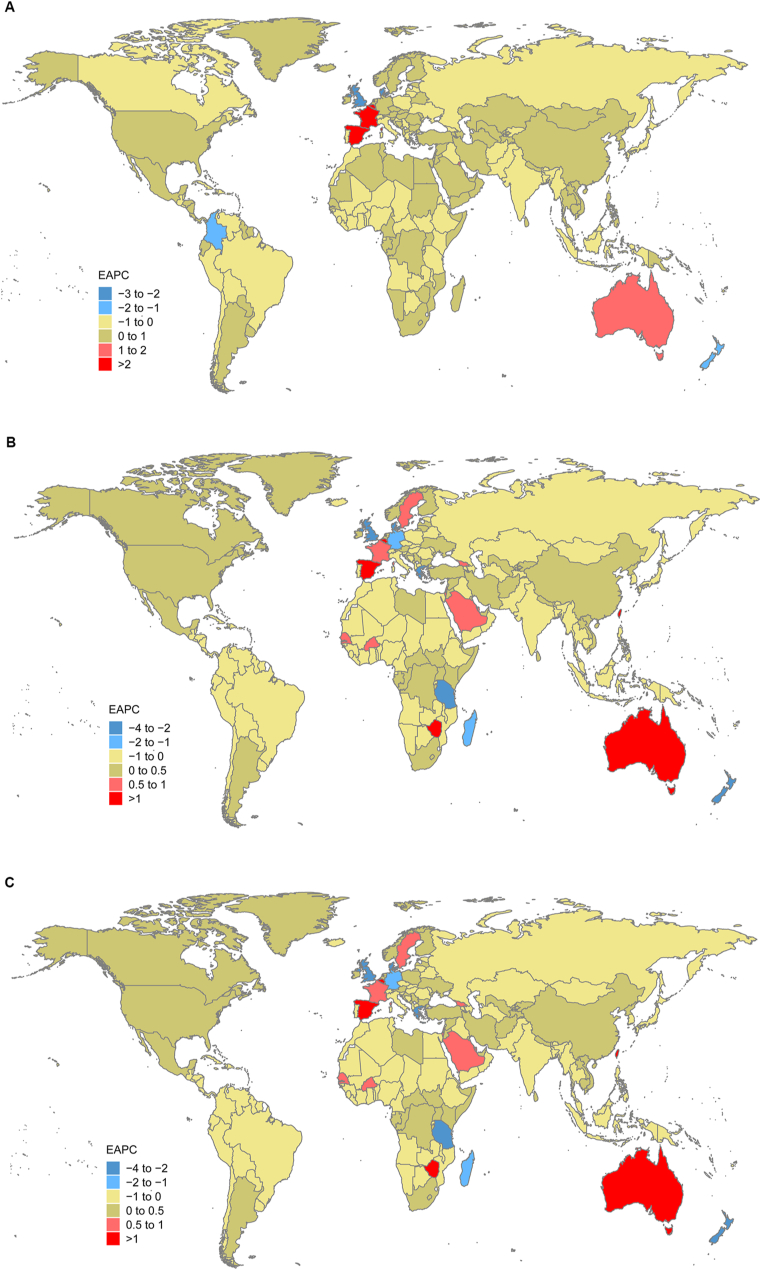
The Estimated Annual Percentage Changes (EAPCs) of untreated caries in deciduous teeth Age-Standardized Rates Worldwide for (a) incidence (b) prevalence (c) YLDs. YLDs, years lived with disability.

The top three regions by age-standardized incidence rate of caries in deciduous teeth in 2019 were Southeast Asia, high-income Asia Pacific, and Australasia, and the top three countries and territories were Cambodia, Kiribati, Lao People's Democratic Republic (appendix Table 2s). The three regions with the lowest age-standardized incidence rates were Central Latin America, Western Europe, and Southern Sub-Saharan Africa, and the three countries and territories with the lowest rates were Botswana, South Africa, and Madagascar (appendix Table 1s). The changing trend of the age-standardized rate of caries in untreated deciduous teeth in different countries is shown in Figure 2(a,b,c).

### 3.3 Burden of untreated dental caries by SDI

**Figure 3:**
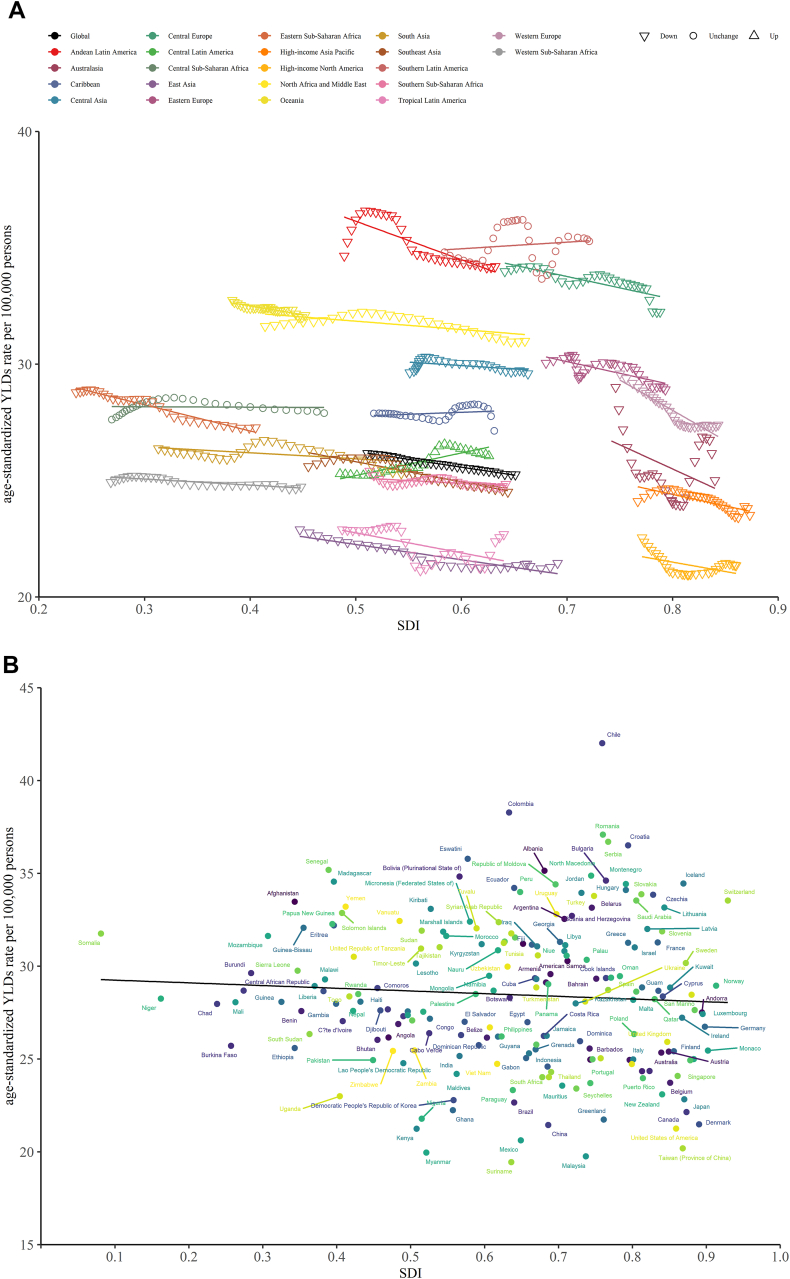
The association between ASYR of untreated caries in permanent teeth and SDI for (a) 21 regions and (b) 204 countries and territories. ASYR, age-standardized years lived with disabilities rate; SDI: sociodemographic index.

An overall negative association was observed between the ASYR of untreated dental caries in permanent teeth and the SDI at the global and regional levels from 1990 to 2019. Except for Southern Latin America, the Caribbean, and Central Sub-Saharan Africa, where the trends were stable, the burden in all regions declined (Figure 3a). As shown in Figure 3a, Andean Latin America, Southern Latin America, and Central Europe had the highest burdens compared with other regions during almost all measurement periods. Figure 3b shows the shape of the association between the ASYR of untreated dental caries in permanent teeth and the SDI for 204 countries and territories in 2019. A decreasing trend in ASYR was observed. High-level SDI regions and countries had lower ASYRs than did low-level SDI regions.

**Figure 4:**
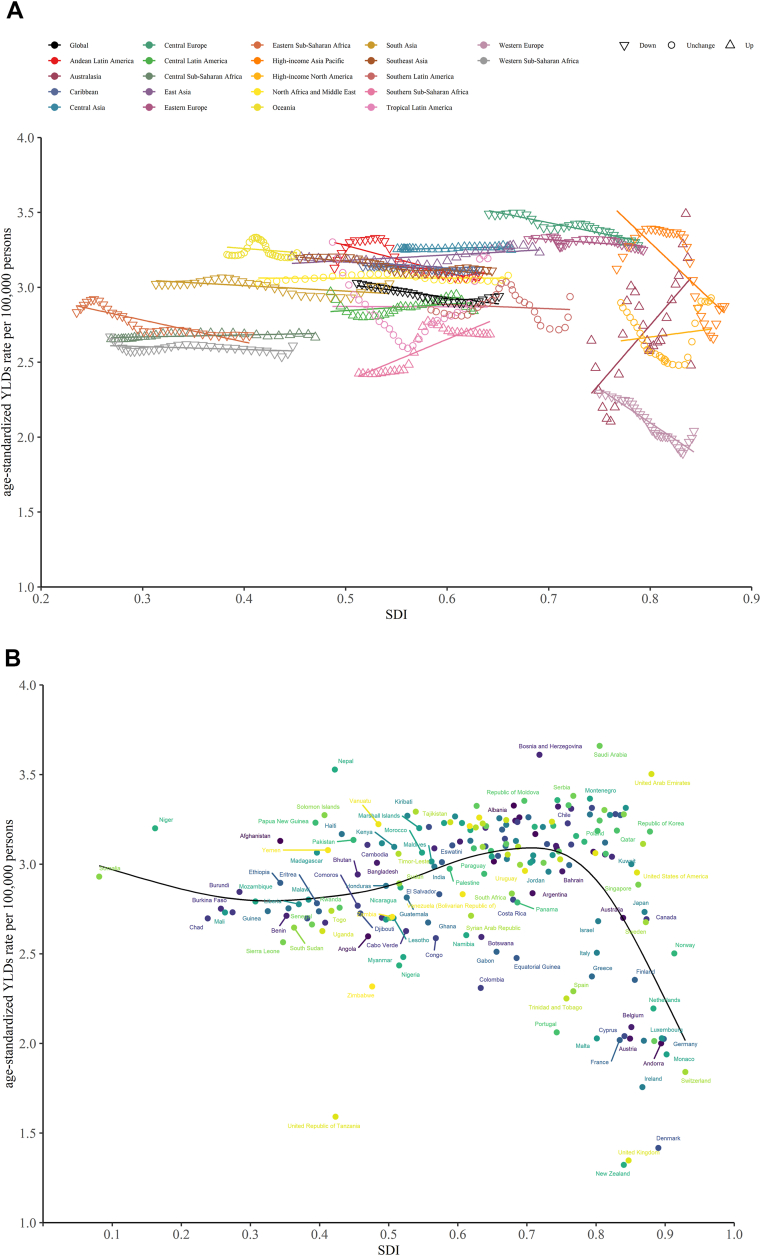
The association between ASYR of untreated caries in deciduous teeth and SDI for (a) 21 regions and (b) 204 countries and territories. ASYR, age-standardized years lived with disabilities rate; SDI: sociodemographic index.

There was a negative correlation between ASYR and regional SDI for deciduous untreated dental caries in most regions during the 1990–2019 period, with a higher ASYR in regions and countries with high levels of SDI than in those with low levels. However, a positive association was found in Australasia, Southern Sub-Saharan Africa, Central Sub-Saharan Africa, and Central Asia (Figure 4a). Figure 4b shows the shape of the association between the ASYR and SDI for the 204 countries and territories in 2019. No clear pattern was observed up to an SDI of 0.75. After this point, a declining burden was observed in most countries and territories for each unit increase in SDI.

### 3.4 Burden of untreated dental caries by age and sex

**Figure 5:**
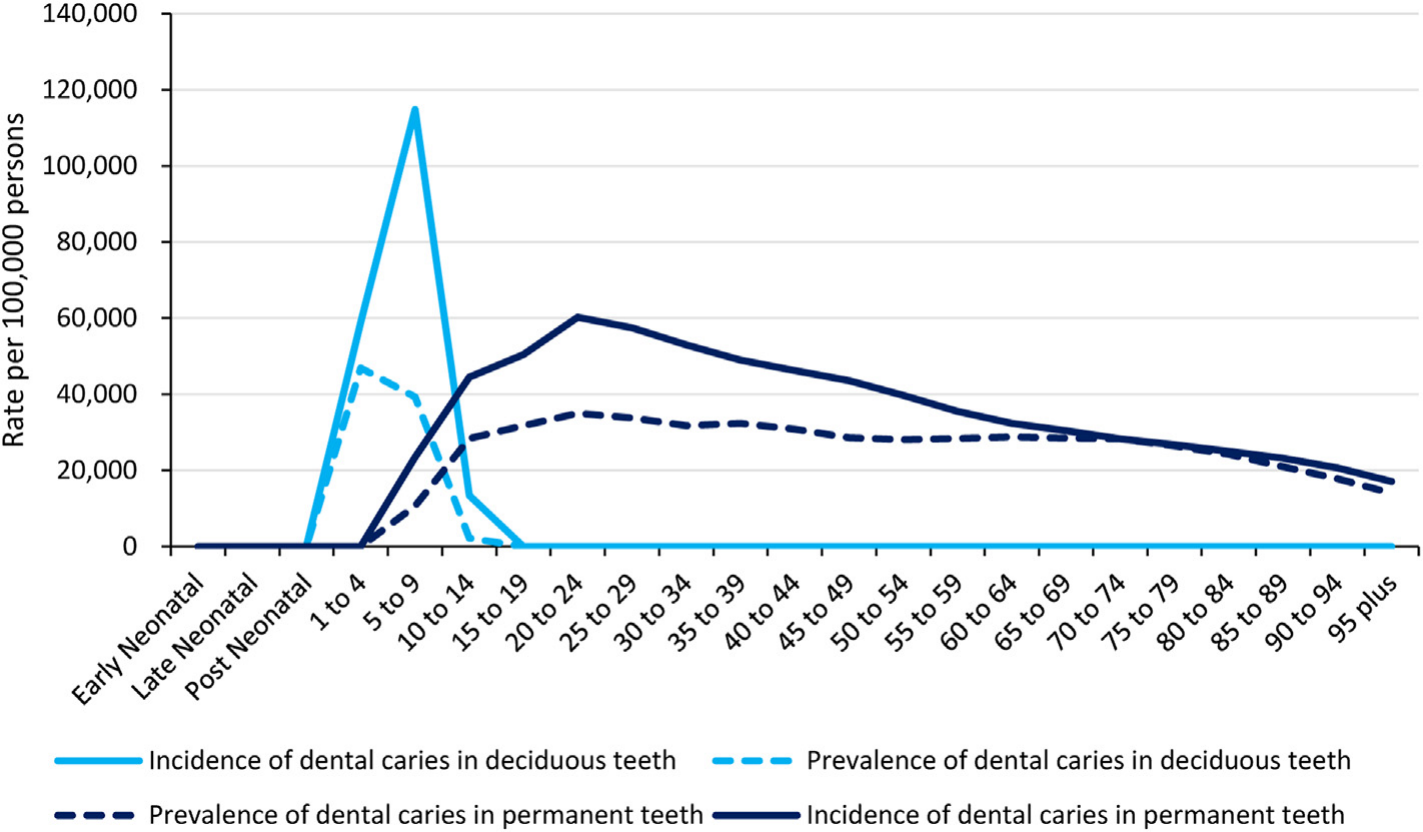
Global age pattern of prevalence and incidence of untreated caries in deciduous teeth and permanent teeth in 2019 for both sexes combined.

The global patterns in the prevalence and incidence of untreated dental caries by age in 2019 are shown in Figure 5. The prevalence and incidence rates in children aged 1–4 years and those over 60 years of age were almost equal, whereas the prevalence rate for people aged 5–59 years was significantly lower than the incidence rate.

**Figure 6:**
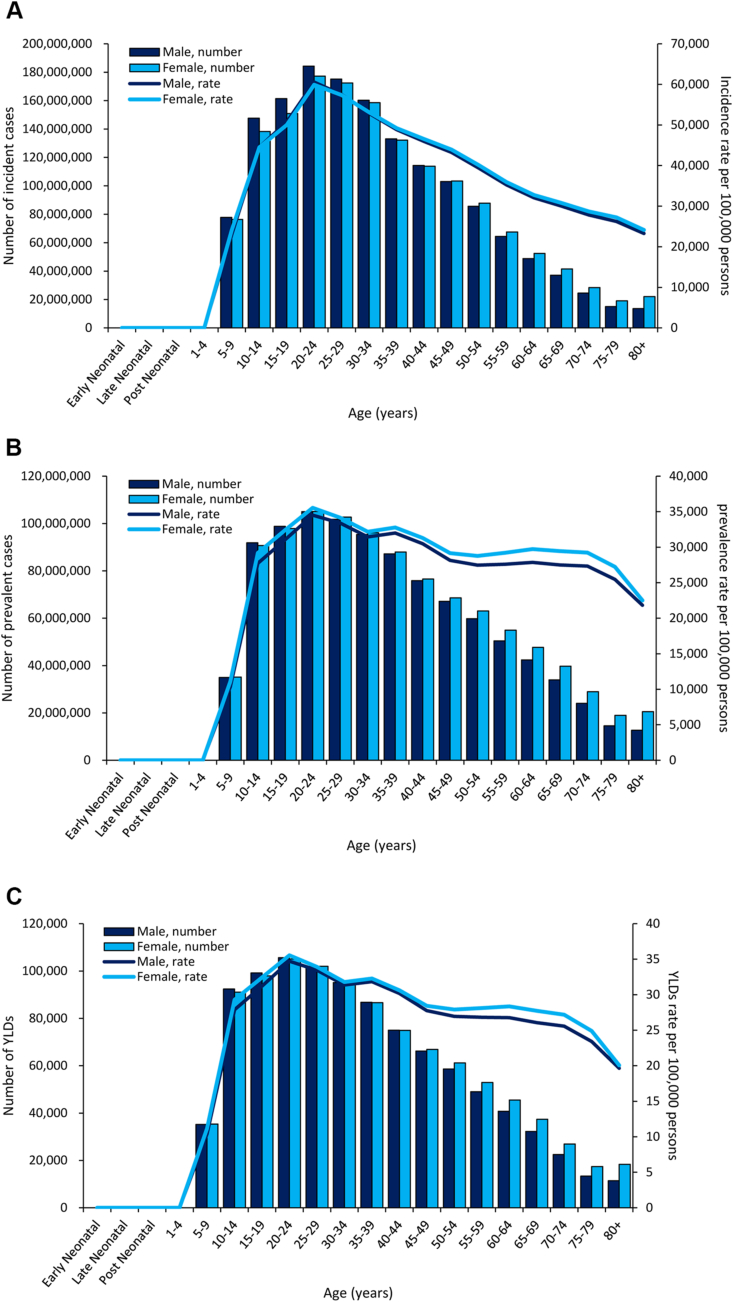
(a) Global incidence and (b) prevalence of and (c) YLDs for untreated caries in permanent teeth by age and sex in 2019. YLDs, years lived with disability.

The 20‒24-year age group had the most incident cases and rate, prevalent cases and rate, and YLDs cases and rate among men and women in 2019 (Figures 6(a,b,c)). Another small peak in the prevalence and YLD rates occurred in the 60‒64-year age group among men (27848.96, 95% UI: 17702.32–42887.02 and 26.77, 95% UI: 9.97–56.61) and women (29738.02, 95% UI: 18864.48–45308.71 and 28.37, 95% UI: 10.52–59.78), respectively.

**Figure 7:**
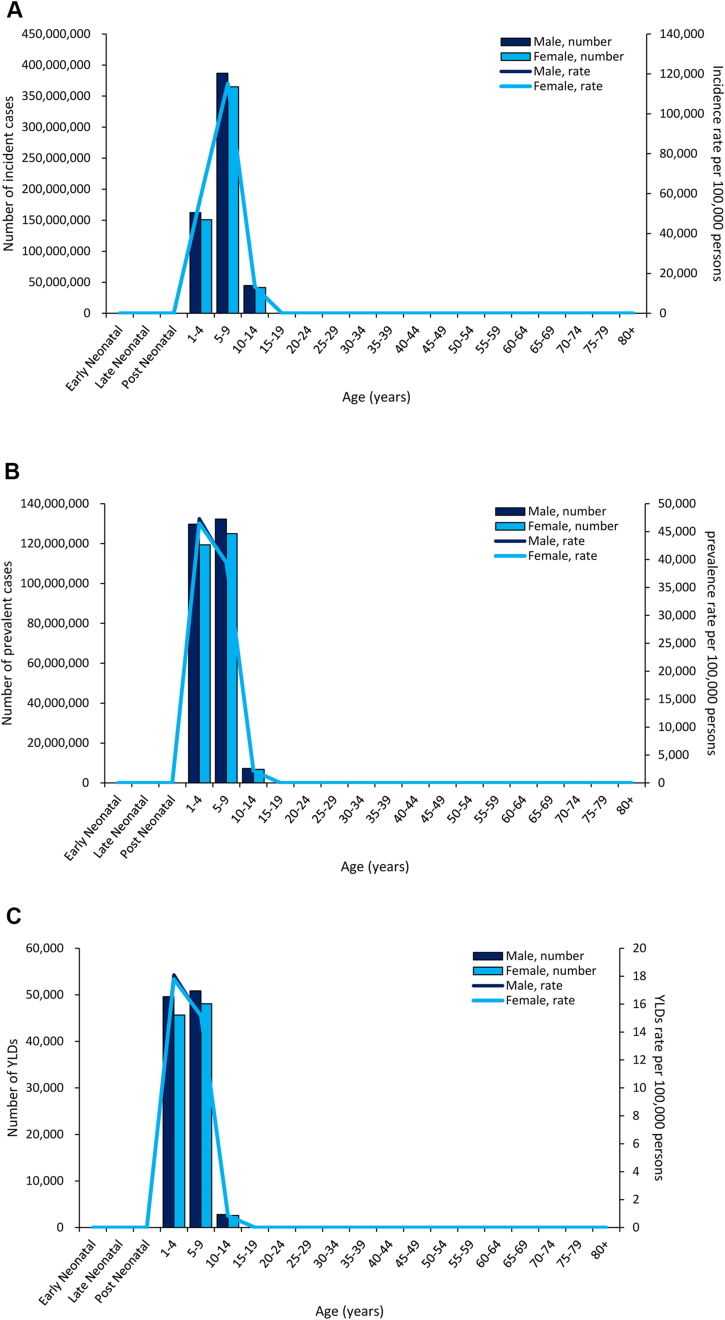
(a) Global incidence and (b) prevalence of and (c) YLDs for untreated caries in deciduous teeth by age and sex in 2019. YLDs, years lived with disability.

The 5‒9-year age group had the most incident cases and incidence rate among male and female children in 2019 (Figure 7a). The 5–9-year age group had the most prevalence and YLDs among male and female children, while the prevalence and YLD rate was highest for the 1‒4-year age group in both male and female children (Figures 7(b, c)).

## 4 Discussion

To the best of our knowledge, this study reports the most up-to-date and comprehensive estimates of the incidence, prevalence, YLDs, and EAPCs of untreated dental caries across 204 countries and territories from 1990 to 2019. The present study shows that the global ASIR of untreated dental caries in permanent teeth showed an upward trend, whereas the ASPR and ASYR showed a downward trend. The global ASIR of untreated dental caries in deciduous teeth showed a stable trend, whereas the ASPR and ASYR showed a downward trend from 1990 to 2019. The ASIR increases or remains stable while the ASPR decreases, the difference between these two values becomes larger, indicating that the treatment rate of caries is increasing. Explain that with symptom-oriented rather than population-level prevention, more resources are allocated to individual treatment. The treatment rate of dental caries is increasing, indicating that the health care system in various countries is constantly improving, especially in high SDI countries and regions, more people are getting dental treatment. However, the incidence of dental caries has not declined, which shows that our prevention work is not good enough, and the coverage of prevention is not wide enough. However, the incidence rate has not declined, indicating that our prevention efforts, and prevention coverage is not broad enough. The ethos and philosophy of dentistry should focus on upstream interventions of oral diseases, and use resources in a fairer, more effective and efficient way to formulate health policies to benefit more people, rather than focus on the downstream interventions of oral diseases, with patients. centered, therapeutic and rehabilitative approach. The numbers of incident cases, prevalent cases, and YLDs increased greatly, although the ASRs of untreated caries increased only slightly. These changes may be due to population growth, aging and changes in people's lifestyles, such as increased free sugar intake.

From 1990 to 2019, the trend of standardized incidence and standardized prevalence of untreated caries varied among 204 countries and regions, possibly due to the speed of economic development and lifestyles in each country and region, especially Eating habits are different. Most of the countries with declining or flat rates of untreated dental caries are those with lower populations per clinical dental provider, such as Brazil, Mexico, USA, Germany, Japan, South Korea, or with lower consumption of sugar-sweetened beverages countries such as Ethiopia, China, despite higher populations per clinical dental provider. The incidence of untreated dental caries is increasing in countries with high intake of sugar-sweetened beverages, such as Barbados, Saint Vincent, Grenadines, the Dominican Republic, and Trinidad and Tobago [14,15]. Therefore, controlling sugar intake is a more effective way to control the rising incidence of untreated dental caries. The countries with the highest number of patients with caries, incidence, and estimated YLDs across all age groups were India, China, and Indonesia. The results of E. Bernabe, et al. also showed that India, China, and Indonesia had the highest demand for dental treatment for caries, periodontal disease, and tooth loss from 1990 to 2017 [16] The likely reason is that these countries have large populations, limited medical resources, and increased need for dental treatment. At the same time, this study also found that the burden of untreated caries was also negatively correlated with the level of socioeconomic development. In areas with high SDI levels, the medical level has improved, the prevalence of untreated caries has decreased, and the disease burden of caries is lower. The impacts of caries disproportionately affect more deprived groups in the society [17], in line with the evidence supporting links between clear socioeconomic inequalities with higher risk of caries lesions or experience among those in lower socioeconomic positions [18]. This highlights the importance of caries prevention requiring attention not only on behavioral and biological risk factors but also on the broader social and environmental determinants of the disease [19].

In this study, there were two peaks of incidence (the 5‒9-year and 20‒24-year age groups) and three peaks of prevalence (the 1‒4-year, 20‒24-year, and 60‒64-year age groups). After reaching the peak incidence of deciduous teeth at the age of 9, the incidence began to decline due to the gradual loss of deciduous teeth, while the prevalence and incidence of untreated caries in permanent teeth peaked at 20–24, probably due to the promotion of oral health to school-aged children, and then in adult life after leaving school ignoring this aspect of health [20]. In addition, the consumption of Sugar-Sweetened Beverages and fruit juices in this age group is the highest of all age groups [15]. At the same time, childhood experiences can affect oral health in adulthood [21]. Peak prevalence at 60–64 years is due to accumulation of untreated caries at the root. This study also found that the prevalence and incidence gap between children aged 1–4 years and older individuals (older than 60 years) was very small, which implies that their rate of caries treatment is very low. There may be several reasons for this phenomenon. Due to poor cooperation during oral treatment, treatment under general anesthesia is often the only realistic approach in young children with extensive caries. This treatment is technically difficult and expensive [22]. Consequently, the treatment rate in preschool children was low. Due to the aging population and increase in life expectancy, the demand for oral treatment by older individuals is increasing. However, because of the complex oral conditions of older people, lack of financial resources, inconvenience in terms of mobility, and other reasons, older individuals have restricted access to dental services, resulting in the accumulation of untreated dental caries [23]. Untreated caries cases are widespread in older adults globally [24]. Providing financial assistance and targeted preventive oral care for these two groups may the greatest public health effects. We also found that the prevalence of permanent tooth caries among women of all ages was higher than among men in 2019. There are three possible reasons for the higher prevalence of caries in women: 1) teeth erupt earlier in women than in men and are therefore exposed to a cariogenic oral environment for longer; 2) hormonal influences (puberty, menstruation, pregnancy, and menopause) increase susceptibility to caries, owing to changes in the oral environment (composition, quantity, and flow rate of saliva) [25].

During the coronavirus disease 2019 (COVID-19) pandemic, the impact of COVID-19 on untreated dental caries has been two-fold. On the one hand, although pulpitis due to dental caries is considered a microbial disease caused mainly by bacteria, viruses have also been implicated in its pathogenesis. Studies have shown that the dental pulp is vulnerable to SARS-CoV2 infection and that SARS-CoV-2 infection of the dental pulp may contribute to worse outcomes of caries specifically pulpitis [26]. On the other hand, the COVID-19 pandemic has had a great impact on the access and utilization of dental services. Both policy factors and personal considerations prevent patients from seeking dental care, except in emergencies. This may lead to an upward trend in the incidence and prevalence of untreated dental caries in the future [27]. Demand for dental services is likely to explode in the post-COVID-19 period. It is recommended to coordinate the power of government administrative departments to implement comprehensive prevention and control measures in future oral care work. Also, during these times, most people's habits have changed negatively. In the context of the novel coronavirus pandemic, telemedicine and science education through short videos on social media for home oral care guidance will become a new direction for medical development.

Nevertheless, this study had some limitations. First, the data presented in our study were obtained from the GBD 2019; some values were estimated rather than directly measured; thus, they may be inaccurate. However, many adjusted methods, including misclassification corrections and redistribution of garbage codes, were used to reduce bias, and many similarities with estimates were reported between the GBD data and other global and national data regarding untreated dental caries. The previous literature and IHME annual reports have confirmed the reliability of this source [5]. Considering that there are very few available recorded data on the burden of untreated dental caries showing long-term trends, the GBD 2019 study provided important information that can be used to evaluate the long-term trends of the disease burden of untreated dental caries. Finally, the interpretation of the results focused on the population level rather than the individual level, rendering it difficult to avoid ecological fallacy; thus, more individual-based studies are needed in the future to confirm the findings in the current study.

## 5 Conclusions

From 1990 to 2019, the ASIR of untreated caries in permanent teeth increased globally, while ASPR and ASYR decreased. Shifting the focus of dentists from a treatment-oriented to a prevention-oriented approach will help to reduce the incidence of dental caries. The global age-standardized incidence, prevalence and YLD of untreated dental caries decreased slightly, but increased significantly in absolute counts, mainly in populous, low SDI regions and countries with an increased unmet need for dental services. Integrating oral health prevention programs with other chronic disease prevention and education programs and policies that share common risk factors is recommended, in addition to developing effective prevention and control strategies to slow the increase in untreated dental caries. Long-term national oral disease epidemiological surveys, especially D/DMFT, are needed for countries to provide reliable documented data on the burden of untreated caries in different countries. The prevalence of untreated dental caries is higher in women than in men and the highest burden of untreated caries was observed in children and the elderly. Broader caries prevention efforts for women, children and the elderly should help reduce the overall burden. Demand for dental services is likely to surge in the post-COVID-19 period and it is recommended that more attention be devoted to the oral cavity to reduce the stress of future dental treatment. In the context of the new coronavirus epidemic, telemedicine and short video science education for home oral care guidance will become another new direction of medical development.

## Declarations

### Author contribution statement

Xiao Feng Qin: Conceived and designed the experiments; Analyzed and interpreted the data; Contributed reagents, materials, analysis tools or data; Wrote the paper.

Hao Zi: Conceived and designed the experiments; Analyzed and interpreted the data; Contributed reagents, materials, analysis tools or data.

Xiao Juan Zeng: Conceived and designed the experiments; Wrote the paper.

### Funding statement

This study was funded by the National Natural Science Foundation of China as part of the Regional Science Foundation Program (grant number: 82060202).

### Data availability statement

Data associated with this study has been deposited at http://ghdx.healthdata.org/gbd-results-tool

### Declaration of interests statement

The authors declare no conflict of interest.

### Additional information

No additional information is available for this paper.
