# Supplementary material for: Changes in the global burden of untreated dental caries from 1990 to 2019: A systematic analysis for the Global Burden of Disease study
Source: Heliyon. 2022 Sep 21;8(9):e10714. doi: 10.1016/j.heliyon.2022.e10714 (PMC9526157; doi:10.1016/j.heliyon.2022.e10714)
Supplement: Supporting Information [file mmc1.docx]

Supporting Information

Changes in the global burden of untreated dental caries from 1990 to 2019: a systematic analysis for the Global Burden of Disease study

Appendix

This appendix provides further methodological detail for “Changes in the global burden of untreated dental caries from 1990 to 2019: a systematic analysis for the Global Burden of Disease study.” Portions of this supplementary methods appendix are reproduced, with permission, from the supplementary appendices of other GBD 2019 publications for analytic components that are common across them. Flowcharts illustrating the overall process of oral disorders modeling are shown in Appendix Figures. Input data sources for all components of GBD estimations are viewable at the Global Health Data Exchange (http://ghdx.healthdata.org). GBD 2019 results are viewable online in an interactive visualization tool called GBD Compare (<http://vizhub.healthdata.org/gbd-compare>).

Oral disorders

This document describes the nonfatal disease burden modeling process for GBD 2019 for each of

edentulism, caries of deciduous teeth, caries of permanent teeth, chronic periodontal disease, and other

oral disorders.

Input data

Data seeking and systematic literature reviews were completed for all oral disorders together given the

overlap in data types and data sources that inform the models. An initial literature review was done by

the Expert Group for GBD 2010 in PubMed, Embase, LILACS, and SciELO, including published articles as

well as the results of national and subnational reports. An updated systematic review was last

completed on February 11, 2018 for GBD 2017 in Pubmed and Embase. The search strings used are

below:

PubMed: ( ( ( (Deciduous caries[Title/Abstract] ) OR (milk caries[Title/Abstract] ) OR (baby caries[Title/Abstract] ) OR (caries[Title/Abstract] ) OR (dental health[Title/Abstract] ) OR (oral health[Title/Abstract]))OR ( (Permanent caries[Title/Abstract] ) OR (caries prevalence[Title/Abstract] ) OR (dental health[Title/Abstract] ) OR (oral health[Title/Abstract] ) ) OR ( (Peridontal disease[Title/Abstract]) OR (periodontitis[Title/Abstract]) OR (periodontal[Title/Abstract] ) ) OR ( (Edentulism[Title/Abstract] ) OR (edentulous[Title/Abstract] ) OR (endentulousness[Title/Abstract] ) OR (severe tooth loss[Title/Abstract] ) OR (total tooth loss[Title/Abstract] ) OR (complete tooth loss[Title/Abstract] ) ) ) AND ( (prevalence[Title/Abstract]) OR (incidence[Title/Abstract]) ) AND (2013/06/01[PDat]: 2016/12/31[PDat] ) )

**Embase**: 'deciduous caries':ab,ti OR 'milk caries':ab,ti OR 'baby caries':ab,ti OR caries:ab,ti OR 'permanent caries':ab,ti OR 'caries prevalence':ab,ti OR 'dental health':ab,ti OR 'oral health':ab,ti OR 'peridontal disease':ab,ti OR periodontitis:ab,ti OR periodontal:ab,ti OR edentulism:ab,ti OR edentulous:ab,ti OR endentulousness:ab,ti OR 'severe tooth loss':ab,ti OR 'total tooth loss':ab,ti OR 'complete tooth loss':ab,ti AND (prevalence:ab,ti OR incidence :ab,ti) AND [2008-2016]/py AND [humans]/lim AND [embase]/lim NOT [medline]/lim

For GBD 2019, we completed an updated systematic review of the Latin American and Caribbean Health Sciences Literature (LILACS) and the Scientific Electronic Library Online (SciELO), focusing first on the most recent period from 2014 to 2018 were subject to full text screening. The search used used for LILACS and SciELO was the same:

**LILACS/ SciELO**: “(deciduous caries OR milk caries OR baby caries OR caries OR dental health OR oral health OR permanent caries OR caries prevalence OR periodontal disease OR periodontitis OR periodontal OR edentulism OR edentulous OR edentulousness OR complete tooth loss OR tooth loss OR toothloss OR number of teeth OR dentate OR edentate) AND (prevalence OR incidence OR survey OR epidemiology)”.

A total of 1696 citations were identified after deduplication, 147 were selected for full text review, and 77 new sources extracted from the following countries: Argentina (1), Brazil (47), Chile (5), Colombia (5), Cuba (5), Ecuador (1), El Salvador (1), Honduras (1), Mexico (5), Peru (5) and Venezuela (1).

We eliminated many data points to avoid repetition in the dataset, while striving to maintain as much data detail as possible. Redundancy tended to arise in three data descriptors: age, gender and urbanicity. Our order of preference for maintaining detail was age, followed by gender, then urbanicity. Additionally, many of the studies presented dmft or DMFT scores, which represent lifetime prevalence and were often described as ”caries experience”. For the purposes of measuring the burden of disability from dental caries, we considered only data on current prevalence to be relevant, and thus converted lifetime prevalence data to current prevalence and incidence where possible. The complete dataset contents for each model are shown in tables for each cause in the corresponding sections below.

**Table** **1.** **Total** **number** **of** **sources** **and** **countries** **with** **data** **for** **Oral** **Disorders,** **by** **measure**

|  | **Total** **sources** | **Countries** **with** **data** |
| --- | --- | --- |
| All measures | 945 | 130 |
| Prevalence | 907 | 130 |
| Incidence | 81 | 40 |
| Proportion | 15 | 1 |
| Other | 22 | 13 |

Caries of permanent teeth and caries of deciduous teeth

Separate estimates of caries of deciduous teeth and caries of permanent teeth

The natural histories of deciduous and permanent caries share many similarities, but they also share some important differences. Age patterns of decay in permanent and deciduous dentition are distinct, and duration of a carious lesion in deciduous teeth also tends to be shorter than an untreated episode of permanent caries. Sugar consumption and feeding with formula are both associated with development of deciduous caries, while sugar consumption is associated with the development of caries of permanent teeth. Finally, it is unclear whether the gender patterns and regional differences are the same for both deciduous and permanent caries. For all of these reasons, we elected to model deciduous caries and permanent caries as separate entities and then add the estimates together for an overall estimation of the global burden of dental caries. This is the modelling approach which has been taken in each iteration since GBD 2010.

Flowchart


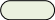

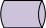

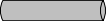

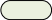

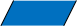

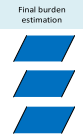

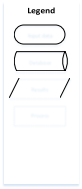

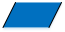

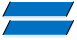

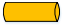

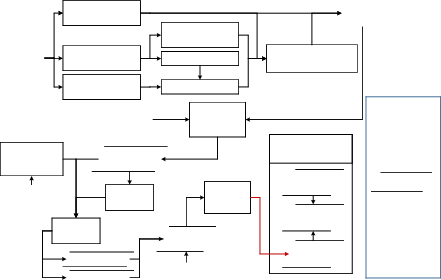


| Caries of deciduous teeth and caries of permanent teeth |
| --- |
| Prevalence of current decay  (d/D>0; reference definition)  Calculate incidence = dmf/DMF  increment by age (<3y gap for  cross-sectional studies)  dmft/DMFT: total and separate  by d/D, m/M, f/F compon ents  (t = reference; s = alternate)  Prevalence of dmf/DMF >0  (alternate definition)  **Location-level** **covariates:**  LN- LDI (prev), Dismod- MR 2 . 1  SEV sugar sweetened beverages (inc)  Meta-analysis of du ration  of pain in those with  caries of deciduous teeth  YLLs  Adjustment for   \| Process \| \| --- \|   Severity splits  YLD by  sequela  To oth pain  Asymptomatic |


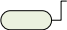


Disability weights Burden estimation

Prevalence & incidence of dental caries

MR- BRT Crosswalk (reference =

current decay and incidence for

teeth; alternate = converted lifetime

Disability weights for each sequela

decay and incidence for surfaces)

Calculate d/dmf or D/DMF ratio

Convert lifetime to current

Comorbidity adjusted

prevalence (d/D>0)

Scientific literature

Scientific literature

edentulism

(permanent only)

Covariates

Input Data

Comorbidity

Survey data

Unadjusted

Nonfatal

correction

Input data


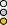


Database

database

Nonfatal

(COMO)

Results


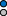


DALYs

YLDs

Case definition

The case definition for dental caries is “teeth with unmistakable coronal cavity at dentin level, root cavity in cementum that feel soft or leathery to probing, temporary or permanent restorations, or missing teeth extracted due to a caries lesion.” Excluded definitions crowns with isolated cosmetic defects, stained enamel pits or fissures without visible cavitation or softening, fluorosis, and abrasion lesions. This definition corresponds to an ICD-9 code of 521.0 and an ICD-10 code of K02.3 – K02.9. Most caries are subclinical in the sense that they do not cause symptoms a majority of the time. Once a carious lesion develops, it will occasionally recede without intervention, but often it worsens with time and eventually requires either filling or extraction.

Public health dentists commonly measure dental caries using the dmft/DMFT index, which is an incremental measure of the proportion of unhealthy teeth and is also a measure of an individual’s lifetime prevalence of caries. Lowercase letters (dmft) are used for deciduous dentition and uppercase letters (DMFT) for permanent dentition. D is for decayed, M for missing, F for filled, and T for teeth. The maximum dmft score is 20 and the maximum DMFT score is 32. Furthermore, some dentists prefer to measure dental caries in terms of tooth surfaces, rather than number of teeth, and report their results using an analogous dmfs/DMFS index. The maximum dmfs score is 88, and the maximum DMFS score is 128 or 148 depending on whether the third molars are counted.

The DMFT index is easy to measure and inter-rater reliability is high. However, the primary shortcoming of the DMFT is that it does not discriminate well between current and past caries. Strategies we employed to maximally utilise dmf/DMF data for estimating the prevalence of burden due to permanent caries are described below.

Input data and data processing

The approach for systematic literature review is described above. The reference definition for this model was presence of one or more teeth with current decay (for prevalence) whereas each additional carious tooth was counted as a separate incident event.

**Table** **1:** **Total** **number** **of** **sources** **and** **countries** **with** **data** **for** **caries** **of** **deciduous** **teeth,** **by** **measure**

|  | **Total** **sources** | **Countries** **with** **data** |
| --- | --- | --- |
| All measures | 419 | 87 |
| Prevalence | 384 | 86 |
| Incidence | 75 | 38 |
| Other | 22 | 13 |

**Table** **2:** **Total** **number** **of** **sources** **and** **countries** **with** **data** **for** **caries** **of** **permanent** **teeth,** **by** **measure**

|  | **Total** **sources** | **Countries** **with** **data** |
| --- | --- | --- |
| All measures | 306 | 91 |
| Prevalence | 306 | 91 |
| Incidence | 6 | 5 |

Converting lifetime to current prevalence

Many of the studies presented dmft or DMFT scores, which represent lifetime prevalence and were often described as “caries experience.” For the purposes of measuring the burden of disability from dental caries, we converted lifetime prevalence data to current prevalence for individuals aged 20 years and less. We did this by multiplying the observed lifetime prevalence by the ratio of d/D to dmf/DMF. When d/dmf or D/DMF information was available from the same study, this ratio was applied. When not available from the same study, the pooled ratio from the closest matching GBD geography was used for the multiplication (country, region, super-region, global).

Calculation of incidence from dmft/DMFT increment

Whereas in the deciduous dentition, a vast majority of the dmf index is accounted for by caries, tooth loss is a major contributor to the DMF index for the permanent dentition. Caries of permanent teeth may not necessarily be the primary driver of this tooth loss, as other factors such as periodontal disease and trauma may contribute significantly. Thus, we performed the conversions of incremental dmf/DMF scores to incidence values for permanent caries only in individuals ages 20 years or less and for all ages in the case of deciduous caries. For longitudinal studies, the difference between the dmf/DMF score in the initial versus subsequent examination was taken to be equivalent to the number of incident caries over that time period. This assumes a negligible proportion of dmf/DMF increment is due to trauma in children. For cross-sectional studies examining children of different ages, we only calculated incidence when the gap in age was three years or fewer given the propensity for strong cohort effects in caries epidemiology.

Age and sex splitting

For any datum that did not entirely fit within a GBD sex or age group, the observation was split to be multiple age-specific and sex-specific data points based on the age and sex pattern predicted by previous DisMod-MR 2.1 models. It is our intention to update with each cycle of GBD.

Crosswalks in MR- BRT

We then crosswalked alternative to reference definitions. In accordance with GBD 2019 principles for data processing, to make data comparable, we began by evaluating the number of observations of each alternate definition that matched with a corresponding observation from the reference definition. The total number of within and between study matches for deciduous caries and permanent caries are shown in the tables below.

**Table** **3:** **Data** **points** **and** **matches** **between** **alternate** **and** **reference** **definitions** **for** **caries** **of** **deciduous** **teeth**

|  | **Prev:** **Reference**  **(d>0)** | **Prev:** **Alternate**  **(cv_d_conversion)** | **Inc:** **Reference**  **(dmft** **increment)** | **Inc:** **Alternate**  **(cv_dmf_units_surfaces)** |
| --- | --- | --- | --- | --- |
| Number of data points | 3783 | 1451 | 1496 | 174 |
| Within-study matches | -- | 2157 | -- | 6 |

**Table** **4:** **Data** **points** **and** **matches** **between** **alternate** **and** **reference** **definitions** **for** **caries** **of** **permanent** **teeth**

|  | **Prev:** **Reference**  **(d>0)** | **Prev:** **Alternate**  **(cv_d_conversion)** | **Inc:** **Reference**  **(dmft** **increment)** | **Inc:** **Alternate**  **(cv_dmf_units_surfaces)** |
| --- | --- | --- | --- | --- |
| Number of data points | 3282 | 1347 | 1650 | 228 |
| Within-study matches | -- | 1648 | -- | 2 |

Owing to the significant heterogeneity in data on caries incidence and prevalence, we limited the comparisons to only “within” study matches where a match was defined as both methods of ascertainment being performed in the identical study population. The ratio of alternative to reference was calculated and logit-transformed. Standard error of the ratio was calculated using the delta method. Sex was included as a fixed effect and, for prevalence only, midpoint of age as a spline. The adjustment factors and spline plots for the crosswalks are shown below.

**Table** **5:** **MR-BRT** **Crosswalk** **Adjustment** **Factors** **for** **caries** **of** **deciduous** **teeth,** **5%** **trim** **for** **prevalence,** **no** **trim** **for** **incidence**

| **Parameter** | **Data** **input** | **Reference** **or** **alternative** **case** **definition** | **Gamma** | **Beta** **Coefficient,** **Logit** **(95%** **CI)** | **Adjustment**  **factor*** |
| --- | --- | --- | --- | --- | --- |
| Prevalence | Current decay | Reference | 0.46 | --- | --- |
| Prevalence | Converted  lifetime decay | Alt |  | -0.15  (-0.29 - 0) | 0.861  (0.748 - 1) |
| Incidence | dmft increment | Reference | 1.11 | --- | --- |
| Incidence | Increment based on surfaces | Alt |  | 0.01  (-0.16 - 0.18) | 1.01 (0.852 - 1.197) |

**Table** **6:** **MR-BRT** **Crosswalk** **Adjustment** **Factors** **for** **caries** **of** **permanent** **teeth,** **20%** **trim** **for** **prevalence,** **no** **trim** **for** **incidence**

| **Parameter** | **Data** **input** | **Reference** **or** **alternative** **case** **definition** | **Gamma** | **Beta** **Coefficient,** **Logit** **(95%** **CI)** | **Adjustment**  **factor*** |
| --- | --- | --- | --- | --- | --- |
| Prevalence | Current decay | Reference | 0.46 | --- | --- |
| Prevalence | Converted  lifetime decay | Alt |  | -0.84  (-2.14 - 0.48) | 0.432 (0.118 - 1.616) |
| Incidence | DMFT increment | Reference | 0.01 | --- | --- |
| Incidence | Increment based on surfaces | Alt |  | 0.03  (-2.27 - 2.21) | 1.03 (0.103 - 9.116) |

**Figure** **1:** **Spline** **plot** **showing** **crosswalk** **value** **by** **age** **group** **for** **alternate** **case** **definition** **of** **converted** **lifetime** **decay** **for** **(a)** **caries** **of** **deciduous** **teeth** **and** **(b)** **caries** **of** **permanent** **teeth**


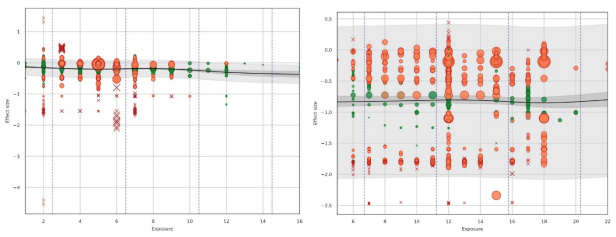

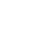

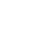


| **a** |  | **b** |
| --- | --- | --- |

Modelling strategy

DisMod model development

Serious health consequences of caries were also assumed to be uncommon and death very rare. We therefore assigned excess mortality to be zero from age 0 to 100. For both types of caries, most of the model settings were similar. The primary difference between the two models was in value priors. We assumed zero incident caries in infants under 1 year old and similarly zero incident deciduous caries from age 11 onward. For permanent caries, we assumed zero incident cases in children under 5 years old. Location-level covariates were assigned separately on prevalence and incidence. Sugar availability in food from the GBD diet analysis was used as a covariate on incidence with a positive beta, while prevalence was assigned log-transformed LDI with a negative beta to reflect the association with access to dental care.

**Table** **6:** **Covariate,** **parameter,** **beta,** **and** **exponentiated** **beta** **values** **for** **dental** **caries**

| **Cause** | **Covariate** | **Param** | **Beta** | **Exponentiated** **beta** |
| --- | --- | --- | --- | --- |
| Deciduous | LN-LDI | Prev | -0.12 ( -0.13 — -0.11) | 0.88 (0.87 — 0.89) |
| Deciduous | SEV High sweetened beverages  (age- and sex-specific) | Inc | 0.65 ( 0.079 — 1.31) | 1.91 (1.08 — 3.72) |
| Permanent | LN-LDI | Prev | -0.17 ( -0.21 — -0.13) | 0.85 (0.81 — 0.88) |
| Permanent | SEV High sweetened beverages  (age- and sex-specific) | Inc | 0.80 ( 0.056 — 1.71) | 2.23 (1.06 — 5.52) |

Although studies were screened carefully during data extraction to ensure that they specified whether they were measuring permanent or deciduous caries, some data points were marked as outliers during modelling due to their high prevalence values in young ages, as it was deemed likely that some of these studies were reporting deciduous in addition to permanent caries. As with deciduous caries, models for permanent caries were vetted based on the plausibility of the results, the extent to which estimates fit the data, and the plausibility of the range of estimates across location hierarchies.

Correction for edentulism

One systematic source of bias in the literature was the exclusion of edentate individuals from the study populations, which leads to systematic overestimation of caries prevalence when modelled over the entire population. To account for this bias, we used our GBD estimates of edentulism prevalence to adjust YLD estimates for caries of permanent teeth. Final DisMod-MR 2.1 estimates of edentulism prevalence were paired with the corresponding results for caries of permanent teeth by age group, sex, location, and year to adjust for the proportion of the population that was excluded from the denominator of permanent caries models. No adjustment was made to the estimates of caries of deciduous teeth.

Severity distributions and disability weights

As described above, the GBD definition of disability associated with symptomatic dental caries is “this person has a toothache, which causes some difficulty eating.” The disability weight associated with this condition is 0.01 (0.005–0.019), as derived from the GBD disability weights study.

Not all those with dental caries experience this disability all the time. We considered only those with active dentinal decay to experience symptomatic tooth pain. Those with deciduous caries who had undergone exfoliation or had their cavities filled were considered to have no disability. Likewise, those with permanent caries who had received fillings, had their cavities extracted, or lost a carious tooth altogether were considered to have no disability. Thus, two additional pieces of information are required to complete the calculation of years of life lived with disability (YLDs): proportion with symptoms and duration of disability.

To determine which segment of the population has ongoing tooth pain and the proportion of time spent with tooth pain, we considered several different options. First, we examined the data on dental caries symptoms and disability from the Medical Expenditure Panel Survey (MEPS) conducted by the USA Department of Health and Human Services in 2000–2009. MEPS data were widely used in GBD 2010 analyses. Respondents to the survey are asked about all medical conditions. Conditions for which provider care was sought are reported by the respondents at every round, and respondents also report problems for which they did not see a provider if the symptoms were “bothering” them. Conditions can be added to the condition roster if 1) they are reported as a reason for a medical event, 2) the condition was reported as the reason for one or more disability days, or 3) the condition was “bothering” the person during the reference period. Conditions are then recorded as verbatim text and coded to ICD- 9CM 3rd digit codes by professional medical coders. These ICD9 codes were mapped to GBD causes, including dental caries. From the MEPS, symptomatic caries in the previous year were reported by 48.4% (95% CI 44.3–52.9) of the respondents. This number is in agreement with our DisMod-MR 2.1 estimates of 1-2 years duration in North America, high-income for permanent caries if we consider people to only have symptoms at the end of a course of caries. The two primary shortcomings of using this approach are 1) it does not provide enough detail to differentiate between the experiences of those with deciduous versus permanent caries, and 2) it indicates the proportion of those with caries who were symptomatic during the previous year, but it does not provide information on the amount of time during that year spent with symptoms (ie, one day versus 12 months). The approach described below addresses both issues.

To determine duration, we adapted the method employed by the Australian Burden of Disease (AusBoD) Study in 1996. For total duration, we used the posterior estimates of duration from final DisMod-MR 2.1 models. For those with symptoms, we split this total duration into two distinct phases of caries disability. The “initial” phase is characterised by *periodic* pain that we assigned to occur an average of one hour per day. The “terminal” phase is a period of *constant* symptoms at the end of an episode. The length of the terminal phase was determined by literature review as described by the AusBoD group. For deciduous caries we used a study by Mason and colleagues of children in the UK presenting to a casualty ward with tooth pain [1]. The length of time each child had been experiencing tooth pain was recorded. Based on the distribution of time courses, a log-normal distribution was plotted that approximated the average duration of *constant* symptoms at 27.6 days leading up to seeking care. For permanent caries, a similar study of the tooth pain experience of adults in New Zealand who presented to hospital dental departments and an emergency clinic [2] resulted in an estimated 55.2 days spent in the terminal phase of caries. For those with severe disease, the length of time spent in the terminal phase was subtracted from the total duration to determine the amount of time spent in the initial phase. For those with mild disease, we considered the entire duration to be spent in the initial phase. These calculations were last completed as part of the GBD 2013 analysis.

To determine proportion with symptoms, we completed a supplemental literature review of tooth pain and caries. We identified a total of 21 studies with data about the prevalence of pain. The studies were grouped according to the type of dentition studied (deciduous or permanent) and the location of the study group (high-income or low- and middle-income countries). We extracted data on the proportion in each group that described symptoms of pain related to their caries as well as a subset who described their symptoms as being severe. The proportions in each group were weighted according to sample size to give estimates of the relative sizes of three groups: asymptomatic, mild, and severe. The results of this meta-analysis are illustrated in the table below.

We considered asymptomatic individuals to experience no disability. Those with mild disease spent the entire duration in the initial phase of disease (one hour of pain per day). Those with severe disease spent a majority of the duration in the initial phase followed by a period of time in the terminal phase (constant pain). YLDs were calculated by multiplying the prevalence, duration, proportion, and disability weight for each age, country, sex, and year.

**Table** **7:** **Duration** **and** **distribution** **of** **severity** **for** **tooth** **pain** **due** **to** **caries** **of** **deciduous** **and** **permanent** **teeth**

|  | **#** **of** **studies** | **%** **symptomatic** **of** **total** | **%** **severe** **among** **symptomatic** | **%** **mild** **of** **total** | **%** **severe** **of** **total** | **%** **asymptomatic** **of** **total** |
| --- | --- | --- | --- | --- | --- | --- |
| Deciduous caries | | | | | | |
| Data-rich | 5 | 0.35 | 0.257 | 0.26 | 0.09 | 0.65 |
| All others | 4 | 0.555 | 0.438 | 0.312 | 0.243 | 0.445 |
| Permanent caries | | | | | | |
| Data-rich | 6 | 0.602 | 0.315 | 0.412 | 0.189 | 0.398 |
| All others | 6 | 0.954 | 0.548 | 0.432 | 0.521 | 0.046 |
| Duration of phases | | | | | | |
| Initial phase | | | | 1 hour per day | | |
| Terminal phase (deciduous caries) | | | | 27.6 days | | |
| Terminal phase (permanent caries) | | | | 55.2 days | | |

| **Table-S1:** **Incident** **cases,** **prevalent** **cases,** **and** **years** **lived** **with** **disabilities** **(YLDs)** **for** **caries** **in** **permanent** **teeth** **in** **2019** **for** **both** **sexes** **and** **the** **estimated** **annual** **percentage** **changes** **(EAPCs)** **from** **1990** **to** **2019** **by** **204** **countries** **and** **territories.** | | | | | | | | | | | | |
| --- | --- | --- | --- | --- | --- | --- | --- | --- | --- | --- | --- | --- |
|  | **Incidence** **(95%** **UI)** | | | | **Prevalence** **(95%** **UI)** | | | | **YLDs** **(95%** **UI)** | | | |
|  | **Counts (2019)** | **Change(%)**  **(1990-2019)** | **ASIR (1/10^5^)**  **(2019)** | **EAPC of ASIR (95% CI)** | **Counts (2019)** | **Change(%)**  **(1990-2019)** | **ASPR (1/10^5^)**  **(2019)** | **EAPC of ASPR (95% CI)** | **Counts (2019)** | **Change(%)**  **(1990-2019)** | **ASYR (1/10^5^)**  **(2019)** | **EAPC of ASYR (95% CI)** |
| Afghanistan | 13850042(115 27161to158930 88) | 255.59% | 38005(32399to 43337) | 0.04(0.02to0.0 6) | 11643128(987 0712to13787243) | 232.84% | 34336(29593to 40048) | -0.09(-0.14to-0.04) | 11498(5126to2 2243) | 235.28% | 33(15to64) | -0.08(-0.13to-0.03) |
| Albania | 1003876(8760 45to1129555) | -11.23% | 35596(30529to 40304) | 0.15(0.12to0.1 7) | 1016995(8701 39to1172808) | -15.71% | 35534(30590to 40970) | -0.17(-0.2to-0.15) | 1001(446to194 1) | -16.42% | 35(16to67) | -0.17(-0.19to-0.15) |
| Algeria | 16468561(143 45150to18741436) | 77.14% | 38710(33857to 43827) | 0.08(0.05to0.1) | 13284758(11361393to15493129) | 75.99% | 31608(27091to 36648) | -0.1(-0.11to-0.09) | 13137(5828to2 5766) | 74.88% | 31(14to61) | -0.1(-0.12to-0.09) |
| American  Samoa | 21960(18994to 24783) | 19.62% | 39019(34030to 44032) | 0.02(0.01to0.0 4) | 17214(14539to 20144) | 26.34% | 30816(26345to 35860) | 0.02(0to0.03) | 17(8to33) | 25.35% | 30(13to60) | 0.01(0to0.02) |
| Andorra | 33568(29601to 37923) | 43.28% | 39288(34306to 43924) | 0.01(0to0.03) | 26445(22386to 30665) | 51.66% | 27916(23994to 32412) | -0.16(-0.19to-0.13) | 26(11to50) | 50.44% | 28(13to55) | -0.16(-0.19to-0.13) |
| Angola | 10678840(890 3785to12253198) | 195.52% | 38401(33314to 43836) | -0.01(-0.03to0.01) | 6796670(5678 999to8029816) | 185.00% | 26638(22704to 31050) | -0.18(-0.22to-0.15) | 6731(3043to13 074) | 186.12% | 26(12to51) | -0.17(-0.2to-0.14) |
| Antigua and  Barbuda | 34816(30773to 39095) | 52.53% | 37506(32933to 42110) | 0.07(0.06to0.0 8) | 23896(20187to 28220) | 49.81% | 25269(21323to 29850) | -0.12(-0.13to- 0.1) | 24(11to47) | 49.13% | 25(11to49) | -0.12(-0.13to-0.11) |
| Argentina | 17138752(14853882to19475675) | 42.50% | 37261(32147to 42405) | 0.05(0.03to0.0 7) | 15385537(13296834to17842334) | 37.43% | 32985(28420to 38291) | -0.08(-0.12to-0.05) | 15149(6770to2 9556) | 37.20% | 33(15to64) | -0.08(-0.11to-0.05) |
| Armenia | 1183807(1047 459to1330981) | -9.49% | 38077(33418to 42871) | 0.02(0to0.04) | 938356(79862 2to1094972) | -10.20% | 29883(25354to 34841) | -0.16(-0.2to- 0.12) | 925(406to1787 ) | -10.65% | 30(13to57) | -0.15(-0.19to-0.11) |
| Australia | 9724912(8445 899to11038763 | 51.75% | 39011(33630to 44086) | 0(-0.12to0.12) | 6794806(5815 834to7852535) | 18.51% | 25814(22199to 29928) | -0.29(-0.57to-0.01) | 6625(2931to12 967) | 17.97% | 25(11to50) | -0.29(-0.57to-0.01) |
| Austria | 3446990(3040 118to3864814) | 12.18% | 38747(33915to 43278) | 0.06(0.04to0.0 8) | 2530333(2143 451to2972280) | 11.70% | 25775(21831to 30026) | -0.14(-0.17to-0.11) | 2470(1084to48 04) | 11.22% | 25(11to50) | -0.13(-0.16to-0.1) |
| Azerbaijan | 4160376(3647 648to4705528) | 51.82% | 38459(33580to 43440) | 0.03(0to0.06) | 3175865(2716 184to3741904) | 48.63% | 29418(25029to 34323) | -0.15(-0.22to-0.08) | 3144(1396to60 76) | 48.11% | 29(13to57) | -0.15(-0.22to-0.08) |
| Bahamas | 149948(13078 7to169515) | 47.90% | 37610(32599to 42235) | 0.02(0.01to0.0 4) | 101886(84961t o121484) | 51.16% | 25240(21074to 30052) | -0.08(-0.09to-0.07) | 101(45to195) | 50.34% | 25(11to48) | -0.08(-0.09to-0.07) |
| Bahrain | 605078(53253 5to686646) | 198.79% | 38893(33874to 43711) | 0.07(0.06to0.0 9) | 470524(39330 4to550936) | 206.73% | 29755(25434to 34288) | -0.09(-0.11to-0.07) | 465(210to905) | 204.80% | 29(13to57) | -0.09(-0.11to-0.07) |
| Bangladesh | 67124672(587 06777to74124875) | 63.88% | 40454(35423to 44632) | 0.04(0.03to0.0 6) | 44207602(379 38984to508168 88) | 60.80% | 27353(23596to 31343) | -0.2(-0.21to- 0.19) | 43599(19455to 84310) | 60.22% | 27(12to52) | -0.19(-0.2to- 0.18) |
| Barbados | 112340(99326t o125762) | 15.09% | 37511(32989to 42105) | 0.05(0.03to0.0 6) | 80555(67913to 94517) | 16.70% | 25834(21853to 30648) | -0.08(-0.09to-0.08) | 79(36to156) | 15.94% | 26(11to49) | -0.09(-0.09to-0.08) |
| Belarus | 3652409(3166 231to4118481) | -7.18% | 37895(32509to 42548) | 0.05(0.03to0.0 7) | 3348417(2880 456to3877724) | -9.34% | 33573(28859to 38929) | -0.15(-0.19to-0.11) | 3278(1460to63 26) | -9.68% | 33(15to64) | -0.14(-0.18to-0.11) |
| Belgium | 4323129(3849 924to4884646) | 9.02% | 38394(33695to 43108) | 0.03(0.01to0.0 4) | 2957837(2492 886to3425428) | 7.60% | 24100(20329to 27756) | -0.12(-0.16to-0.08) | 2883(1263to57 03) | 6.99% | 24(11to47) | -0.13(-0.16to-0.09) |
| Belize | 159025(13894 1to179191) | 140.18% | 36901(32474to 41338) | 0.05(0.03to0.0 7) | 111919(93226t o131827) | 134.64% | 26472(22284to 31107) | -0.13(-0.15to-0.12) | 111(50to218) | 133.82% | 26(12to51) | -0.14(-0.16to-0.12) |
| Benin | 4349586(3654 209to4978300) | 173.58% | 37083(32119to 42029) | 0.03(0to0.06) | 3084193(2580 479to3636734) | 169.49% | 27973(23947to 32735) | -0.1(-0.12to-0.08) | 3063(1370to57 90) | 170.33% | 28(12to52) | -0.09(-0.11to-0.07) |
| Bermuda | 23951(21090to 27172) | 1.89% | 38169(33548to 42527) | 0.07(0.05to0.0 9) | 16262(13676to 19223) | 1.93% | 24577(20878to 28895) | -0.13(-0.14to-0.12) | 16(7to31) | 1.07% | 24(11to47) | -0.13(-0.14to-0.12) |
| Bhutan | 325245(29220 0to355249) | 36.78% | 40469(36209to 44255) | 0.06(0.04to0.0 9) | 204505(17580 3to235172) | 30.60% | 26486(22855to 30412) | -0.23(-0.25to-0.22) | 202(91to387) | 30.18% | 26(12to50) | -0.22(-0.24to-0.2) |
| Bolivia (Plurinational State of) | 4262184(3674 746to4904755) | 97.84% | 35751(31089to 41148) | 0.06(0.05to0.0 8) | 4121203(3487 947to4762364) | 91.63% | 35259(30285to 40591) | -0.07(-0.07to-0.07) | 4084(1802to82 20) | 91.53% | 35(16to70) | -0.06(-0.06to-0.05) |
| Bosnia and Herzegovina | 1232775(1046 975to1428443) | -25.17% | 35892(30091to 41423) | 0.16(0.12to0.1 9) | 1154172(9737 72to1341280) | -28.47% | 33161(28005to 38674) | -0.23(-0.28to-0.17) | 1129(501to215 9) | -29.27% | 33(15to63) | -0.23(-0.28to-0.18) |
| Botswana | 929582(81101 4to1059897) | 93.55% | 38432(33624to 43748) | 0.06(0.04to0.0 8) | 688169(57948 5to806646) | 96.57% | 28959(24558to 33708) | -0.14(-0.16to-0.12) | 676(306to1287 ) | 95.28% | 28(13to53) | -0.15(-0.17to-0.13) |
| Brazil | 86664551(776 87458to95635207) | 48.97% | 38292(34008to 42428) | -0.06(-0.13to0.02) | 52129780(450 31137to600714 27) | 52.53% | 22994(19807to 26462) | -0.22(-0.33to-0.11) | 51312(23299to 98928) | 51.90% | 23(10to44) | -0.22(-0.32to-0.11) |
| Brunei  Darussalam | 181277(16064 7to203390) | 81.22% | 37973(33437to 42706) | 0.01(0to0.02) | 116498(99220t o137104) | 87.30% | 24679(21099to 28863) | -0.05(-0.06to-0.05) | 115(52to215) | 86.39% | 24(11to45) | -0.05(-0.05to-0.04) |
| Bulgaria | 2573550(2221 754to2976532) | -17.21% | 35970(30712to 41474) | 0.11(0.09to0.1 3) | 2527649(2157 686to2921040) | -22.11% | 34986(30148to 40660) | -0.11(-0.13to-0.08) | 2474(1085to47 73) | -22.53% | 35(15to67) | -0.1(-0.13to-0.07) |
| Burkina Faso | 7824130(6686 909to8928079) | 149.73% | 37687(33192to 41993) | 0.07(0.04to0.1) | 5130822(4277 666to6087780) | 139.42% | 26051(22126to 30465) | -0.38(-0.46to-0.29) | 5097(2265to98 96) | 140.95% | 26(12to49) | -0.35(-0.44to-0.27) |
| Burundi | 4301869(3611 509to4935079) | 124.11% | 38373(33504to 43765) | 0.04(0.02to0.0 7) | 3124208(2617 886to3695528) | 122.20% | 30221(25796to 35254) | 0(-0.02to0.01) | 3087(1383to60 73) | 122.22% | 30(13to57) | 0(-0.01to0.01) |
| Cabo Verde | 220904(19041 7to250354) | 82.81% | 37757(32431to 42679) | 0.09(0.07to0.1 1) | 153864(13135 8to180693) | 76.91% | 26691(22850to 31310) | -0.22(-0.23to-0.21) | 153(68to296) | 76.63% | 26(12to51) | -0.22(-0.23to-0.21) |
| Cambodia | 6824938(5972 798to7673207) | 80.50% | 40276(35262to 45298) | 0.07(0.05to0.0 9) | 4664620(3983 108to5373852) | 76.96% | 28014(24220to 32163) | -0.06(-0.11to-0.01) | 4626(2074to87 98) | 77.18% | 28(12to53) | -0.04(-0.09to0.01) |
| Cameroon | 10624529(904 3405to12117334) | 202.93% | 37579(32599to 42241) | 0.07(0.05to0.0 9) | 7432328(6133 364to8883482) | 201.85% | 27712(23351to 32700) | -0.02(-0.04to0) | 7378(3213to14 317) | 203.36% | 27(12to53) | -0.01(-0.02to0.01) |
| Canada | 14250456(125 94461to159026 08) | 29.43% | 39046(34404to 43598) | 0(-0.02to0.02) | 8694198(7415 879to10156111 ) | 31.64% | 22438(19128to 26146) | -0.06(-0.08to-0.05) | 8514(3801to16 508) | 30.67% | 22(10to43) | -0.07(-0.08to-0.05) |
| Central African Republic | 1927226(1614 259to2200915) | 100.69% | 37873(32649to 42965) | 0.01(-0.01to0.03) | 1367621(1159 155to1610255) | 104.25% | 29330(25299to 34129) | 0.04(0.03to0.0 5) | 1349(607to259 8) | 104.79% | 29(13to55) | 0.05(0.04to0.0 6) |
| Chad | 5432069(4551 906to6248025) | 175.49% | 37813(32914to 42553) | 0.07(0.05to0.0 9) | 3803003(3122 277to4557450) | 160.77% | 28419(23977to 33242) | -0.15(-0.16to-0.13) | 3774(1647to73 73) | 161.46% | 28(12to54) | -0.14(-0.16to-0.13) |
| Chile | 6136916(5461 107to6654164) | 28.60% | 33274(29538to 36341) | -0.1(-0.37to0.17) | 8439113(8045 453to8853182) | 70.01% | 42672(40466to 45165) | 0.25(-0.09to0.59) | 8280(3680to16 157) | 68.73% | 42(19to82) | 0.25(-0.09to0.58) |
| China | 580601994(52 8464826to6371 58149) | 17.41% | 39190(35282to 43104) | -0.05(-0.07to-0.02) | 330136487(28 5836779to3811 28922) | 15.84% | 21614(18622to 24960) | -0.27(-0.31to-0.23) | 326163(14327 4to633995) | 15.07% | 21(10to41) | -0.26(-0.31to-0.22) |
| Colombia | 15305892(135 44271to17741371) | 31.52% | 31174(27352to 36255) | -0.57(-0.68to-0.46) | 19407752(161 37608to211955 78) | 103.63% | 38811(32325to 42412) | 1.19(0.97to1.4 1) | 19125(8353to3 6933) | 102.30% | 38(17to74) | 1.2(0.98to1.42) |
| Comoros | 278894(24277 0to312975) | 68.29% | 38452(33646to 43013) | 0(-0.02to0.02) | 205215(17230 7to239880) | 73.79% | 29227(24686to 34191) | -0.05(-0.07to-0.04) | 203(91to394) | 73.62% | 29(13to56) | -0.04(-0.06to-0.03) |
| Congo | 1977723(1685 061to2259028) | 129.03% | 38261(33119to 43208) | 0.03(0.03to0.0 4) | 1315285(1093 574to1552609) | 134.25% | 26766(22485to 31434) | -0.03(-0.04to-0.01) | 1299(580to250 5) | 134.47% | 26(12to51) | -0.02(-0.03to0) |
| Cook Islands | 6977(6190to78 36) | -4.26% | 38970(34388to 43516) | 0.06(0.04to0.0 8) | 5554(4755to64 65) | -2.17% | 29826(25252to 34625) | -0.11(-0.13to-0.09) | 5(2to11) | -3.48% | 29(13to57) | -0.11(-0.14to-0.09) |
| Costa Rica | 1754863(1528 340to1959614) | 62.59% | 35746(30872to 40097) | 0.04(0.03to0.0 6) | 1312854(1118 529to1529820) | 61.70% | 26582(22482to 31005) | -0.18(-0.19to-0.18) | 1295(580to251 6) | 60.52% | 26(12to51) | -0.18(-0.19to-0.18) |
| Croatia | 1545308(1352 404to1749221) | -12.85% | 35067(30417to 40079) | 0.04(0.02to0.0 5) | 1652935(1422 261to1905885) | -13.07% | 36978(31946to 42471) | 0.09(0.02to0.1 6) | 1614(701to301 8) | -13.67% | 37(16to70) | 0.1(0.02to0.17) |
| Cuba | 4250212(3810 336to4888895) | 1.41% | 37291(33020to 42499) | -0.1(-0.15to-0.04) | 3600587(3090 680to4213276) | 5.47% | 29731(25395to 34771) | 0.38(0.24to0.5 1) | 3529(1558to69 25) | 4.50% | 29(13to58) | 0.38(0.24to0.51) |
| Cyprus | 529407(47681 3to588376) | 72.73% | 39028(34811to 43222) | 0.09(0.07to0.1 1) | 418470(35960 4to484548) | 72.49% | 28805(24618to 33427) | -0.15(-0.17to-0.13) | 410(179to784) | 71.52% | 28(13to54) | -0.15(-0.17to-0.13) |
| Czechia | 3941234(3444 570to4572285) | 3.76% | 36107(30833to 41466) | 0.08(0.06to0.1) | 3758276(3198 909to4382791) | -0.85% | 34304(29433to 40065) | -0.09(-0.1to- 0.07) | 3666(1622to69 23) | -1.69% | 34(15to66) | -0.09(-0.1to-0.08) |
| Cote d'Ivoire | 9521334(8136 063to10832112 | 128.82% | 37875(33103to 42799) | 0.07(0.05to0.0 8) | 6582429(5504 307to7753007) | 127.51% | 27481(23401to 31841) | -0.01(-0.03to0.01) | 6520(2950to12 493) | 128.25% | 27(12to52) | 0(-0.02to0.02) |
| Democratic People's  Republic of  Korea | 10844697(962 5726to12053961) | 35.45% | 39288(34607to 43574) | 0.07(0.06to0.0 9) | 6442902(5538 095to7464971) | 41.25% | 22957(19595to 26724) | 0.11(0.09to0.1 4) | 6381(2857to12 115) | 40.65% | 23(10to44) | 0.11(0.09to0.1 4) |
| Democratic Republic of the Congo | 32193296(275 44095to36807285) | 141.85% | 38446(33810to 43519) | 0.01(-0.01to0.02) | 22394740(189 67159to26197633) | 146.81% | 29269(25160to 33946) | 0.07(0.02to0.1 2) | 22102(10073to 42790) | 148.06% | 29(13to54) | 0.09(0.05to0.1 4) |
| Denmark | 2148460(1942 370to2365954) | 6.97% | 37392(33399to 41275) | -0.06(-0.09to-0.03) | 1349778(1136 365to1591871) | 5.85% | 21779(18310to 25742) | -0.31(-0.36to-0.26) | 1321(594to2568) | 5.73% | 21(10to42) | -0.31(-0.36to-0.26) |
| Djibouti | 460076(40092 2to519476) | 162.49% | 38446(33853to 43313) | 0.03(0.01to0.0 4) | 324109(27299 3to383103) | 171.26% | 28008(23890to 32715) | -0.05(-0.09to-0.01) | 321(145to625) | 170.27% | 28(12to54) | -0.05(-0.08to-0.01) |
| Dominica | 26396(22930to 29948) | -2.51% | 37507(32334to 42509) | 0.04(0.03to0.0 5) | 18898(16115to 22333) | -4.24% | 26306(22349to 30970) | -0.16(-0.17to-0.15) | 19(8to36) | -4.85% | 26(12to50) | -0.16(-0.17to- 0.15) |
| Dominican  Republic | 4185452(3619 925to4679019) | 57.94% | 37600(32524to 42184) | 0.1(0.09to0.11) | 2876224(2455 427to3389549) | 51.89% | 26026(22243to 30639) | -0.19(-0.19to-0.18) | 2850(1275to56 17) | 51.50% | 26(12to51) | -0.19(-0.19to-0.18) |
| Ecuador | 6484249(5673 322to7353456) | 84.19% | 36178(31682to 40939) | 0.24(0.16to0.31) | 6145900(5240 745to7084129) | 82.17% | 34615(29572to 39832) | -0.62(-0.84to-0.39) | 6087(2648to12 053) | 81.45% | 34(15to67) | -0.62(-0.85to-0.39) |
| Egypt | 38625467(339 08671to42682734) | 90.00% | 38360(34175to 42229) | 0.11(0.08to0.13) | 26838238(227 30300to31612091) | 79.32% | 27292(23243to 31941) | -0.13(-0.16to- 0.1) | 26643(11890to 52600) | 79.28% | 27(12to52) | -0.13(-0.16to-0.1) |
| El Salvador | 2290584(1985 392to2575014) | 26.18% | 35574(30713to 40001) | 0.05(0.04to0.0 7) | 1741048(1464 206to2048859) | 21.59% | 27399(23189to 32295) | -0.2(-0.21to- 0.19) | 1718(769to335 0) | 21.22% | 27(12to52) | -0.19(-0.2to-0.19) |
| Equatorial  Guinea | 559051(48413 2to627161) | 290.47% | 39046(34485to 43118) | 0.06(0.05to0.0 8) | 329702(27514 4to389639) | 232.83% | 25045(21288to 29160) | -0.65(-0.75to-0.55) | 327(147to641) | 236.50% | 25(11to48) | -0.61(-0.71to-0.52) |
| Eritrea | 2514630(2179 891to2850081) | 137.69% | 37568(33143to 42044) | 0.02(0to0.04) | 2060078(1756 832to2417294) | 145.53% | 32766(28279to 37794) | 0(-0.03to0.04) | 2042(902to398 8) | 147.15% | 32(14to62) | 0.03(-0.01to0.06) |
| Estonia | 508873(44945 3to575879) | -15.26% | 38555(33546to 43421) | 0.09(0.07to0.1) | 393299(33269 1to461946) | -18.31% | 29007(24542to 34421) | -0.13(-0.19to-0.08) | 385(172to728) | -18.63% | 29(13to56) | -0.12(-0.18to- 0.07) |
| Eswatini | 425954(36250 4to488839) | 53.36% | 36722(31637to 41692) | 0.04(0.03to0.0 6) | 397054(33911 4to457848) | 56.81% | 36714(31915to 42012) | -0.08(-0.09to-0.07) | 390(169to751) | 55.53% | 36(16to69) | -0.11(-0.12to- 0.1) |
| Ethiopia | 41275151(356 11098to46358886) | 124.41% | 39738(35238to 44380) | 0.01(-0.01to0.02) | 24971873(209 79972to29154837) | 116.85% | 26009(22355to 30156) | -0.16(-0.19to-0.13) | 24763(11153to 48049) | 117.80% | 26(12to50) | -0.14(-0.17to- 0.11) |
| Fiji | 359494(31451 0to402598) | 22.96% | 38856(34046to 43458) | 0.05(0.04to0.0 7) | 289787(24638 7to336447) | 23.80% | 31753(27196to 36668) | -0.15(-0.27to-0.03) | 285(125to552) | 22.86% | 31(14to60) | -0.16(-0.28to-0.03) |
| Finland | 2110415(1841 650to2372166) | 7.40% | 38773(33411to 43267) | 0.01(-0.02to0.04) | 1563643(1333 265to1818678) | 7.28% | 25817(22320to 29938) | 0.06(-0.06to0.18) | 1523(661to298 0) | 6.68% | 25(11to50) | 0.06(-0.06to0.18) |
| France | 24871385(223 66370to27664289) | 12.17% | 38338(34237to 42852) | 0.06(0.05to0.0 8) | 23034785(199 80898to26558128) | 3.01% | 31714(27365to 36627) | -0.32(-0.44to-0.2) | 22518(10121to 42399) | 2.49% | 31(14to60) | -0.32(-0.44to-0.2) |
| Gabon | 683819(58983 4to768945) | 93.39% | 38537(33481to 43160) | 0.01(0to0.03) | 435482(36816 8to511867) | 95.82% | 25538(21730to 29721) | 0(-0.01to0.02) | 429(193to833) | 95.67% | 25(11to48) | 0.01(0to0.02) |
| Gambia | 816966(69561 8to939775) | 145.42% | 37434(32706to 42821) | 0.04(0.03to0.0 6) | 587586(49210 8to696904) | 144.10% | 28428(24211to 33368) | -0.04(-0.05to-0.02) | 582(254to1120 ) | 144.20% | 28(12to53) | -0.04(-0.05to-0.02) |
| Georgia | 1400335(1216 992to1574395) | -34.24% | 37778(32648to 42132) | -0.07(-0.11to-0.04) | 1192975(1023 320to1373366) | -33.08% | 31686(27110to 36687) | 0.14(0.08to0.2) | 1170(510to230 2) | -33.60% | 31(14to60) | 0.13(0.07to0.1 9) |
| Germany | 32821197(290 77088to365750 42) | 2.31% | 39212(34659to 43453) | -0.05(-0.08to-0.03) | 25633876(216 10147to297093 58) | 1.06% | 27150(23373to 31388) | -0.4(-0.49to-0.32) | 24948(10826to 49406) | 0.53% | 27(12to52) | -0.4(-0.49to-0.31) |
| Ghana | 11678083(995 2249to1328488 8) | 128.11% | 36728(31603to 41773) | 0.06(0.03to0.1) | 6966479(5752 543to8315512) | 124.64% | 22516(18827to 26682) | -0.11(-0.14to-0.08) | 6918(3094to13 410) | 125.28% | 22(10to43) | -0.1(-0.13to-0.07) |
| Greece | 3939571(3473 090to4435581) | 2.81% | 38886(34146to 43441) | 0.19(0.13to0.2 4) | 3700433(3191 967to4232911) | -14.46% | 31737(27463to 36587) | -0.76(-0.95to-0.57) | 3604(1591to68 90) | -15.42% | 31(14to60) | -0.77(-0.96to-0.58) |
| Greenland | 22626(20067to 25233) | -3.18% | 39207(34623to 43712) | -0.03(-0.05to-0.01) | 13090(11110to 15241) | -0.75% | 22106(18893to 25776) | -0.1(-0.12to- 0.09) | 13(6to25) | -1.22% | 22(10to42) | -0.09(-0.11to- 0.08) |
| Grenada | 39824(35329to 44571) | 28.79% | 36943(32505to 41606) | 0.04(0.02to0.0 5) | 28182(23966to 33056) | 25.95% | 25857(21968to 30291) | -0.22(-0.24to-0.21) | 28(12to54) | 25.28% | 26(11to49) | -0.23(-0.24to-0.22) |
| Guam | 67286(58539to 76086) | 21.15% | 39520(34323to 44679) | 0.07(0.04to0.0 9) | 50312(42899to 58354) | 25.79% | 29225(24910to 33952) | -0.03(-0.04to-0.02) | 50(22to99) | 24.66% | 29(13to57) | -0.04(-0.05to-0.02) |
| Guatemala | 6588381(5714 893to7453248) | 152.20% | 35848(31296to 40346) | 0.03(0.01to0.0 5) | 4857171(4101 513to5700088) | 142.49% | 27613(23535to 32385) | -0.15(-0.16to-0.14) | 4807(2158to9421) | 142.67% | 27(12to52) | -0.14(-0.15to-0.13) |
| Guinea | 4400869(3692 536to5047429) | 114.67% | 37580(32598to 42583) | 0.05(0.02to0.0 7) | 3142972(2633 694to3712399) | 107.84% | 28521(24096to 33292) | -0.02(-0.05to0) | 3116(1381to60 66) | 108.39% | 28(13to54) | -0.02(-0.04to0) |
| Guinea-Bissau | 679604(56796 5to779978) | 103.42% | 36973(31517to 41993) | 0.06(0.04to0.0 7) | 559450(47125 3to656241) | 86.47% | 32594(27877to 37780) | -0.09(-0.12to-0.06) | 555(246to1085 ) | 86.88% | 32(14to62) | -0.09(-0.12to- 0.06) |
| Guyana | 297769(25958 8to335107) | 3.55% | 37117(32208to 41995) | 0.04(0.01to0.0 7) | 211817(18030 9to247727) | -0.40% | 26659(22734to 31260) | -0.21(-0.23to-0.19) | 209(93to398) | -0.77% | 26(12to50) | -0.21(-0.23to-0.19) |
| Haiti | 4644494(3949 464to5270853) | 108.97% | 36882(31492to 41725) | 0.05(0.03to0.0 6) | 3493742(2939 715to4119908) | 107.01% | 28604(24207to 33595) | -0.06(-0.07to-0.05) | 3448(1554to67 52) | 106.74% | 28(13to55) | -0.06(-0.07to-0.05) |
| Honduras | 3551577(3058 326to4027802) | 129.61% | 35287(30847to 39594) | 0.05(0.03to0.0 6) | 2697403(2249 289to3184123) | 123.86% | 27934(23679to 32718) | -0.12(-0.14to-0.11) | 2677(1175to53 50) | 123.67% | 28(12to54) | -0.12(-0.14to-0.11) |
| Hungary | 3593950(3181 855to4091305) | -4.06% | 35871(31259to 40718) | 0.13(0.11to0.1 5) | 3496205(2990 406to4084292) | -12.60% | 34496(29695to 39962) | -0.31(-0.34to-0.28) | 3421(1505to66 46) | -13.00% | 34(15to66) | -0.3(-0.33to-0.27) |
| Iceland | 129959(11398 5to145687) | 36.78% | 37489(32378to 42089) | 0.28(0.23to0.3 4) | 131012(11412 5to149839) | 33.09% | 34969(30569to 40135) | -0.41(-0.5to-0.32) | 128(57to247) | 32.37% | 34(15to68) | -0.41(-0.5to-0.32) |
| India | 591137582(52 8315682to6470 19004) | 75.78% | 40586(36215to 44390) | 0.03(0.02to0.0 4) | 366858183(31 7135044to4193 62762) | 75.81% | 25743(22405to 29403) | -0.05(-0.12to0.02) | 360039(16202 7to692198) | 75.72% | 25(11to48) | -0.03(-0.1to0.04) |
| Indonesia | 111444001(98 899131to12227 2277) | 49.36% | 40913(36232to 44914) | 0.05(0.04to0.0 6) | 69024654(592 47927to79063232) | 47.69% | 25633(22017to 29329) | -0.05(-0.07to-0.02) | 68320(31107to 134517) | 47.41% | 25(12to49) | -0.03(-0.06to-0.01) |
| Iran (Islamic Republic of) | 34399378(304 07215to38371486) | 57.58% | 39350(34510to 43747) | 0.01(-0.01to0.04) | 25924109(221 54379to30208979) | 68.56% | 29766(25545to 34481) | 0.04(-0.05to0.12) | 25547(11328to 50618) | 67.45% | 29(13to58) | 0.05(-0.04to0.13) |
| Iraq | 16780353(146 58618to18737013) | 171.88% | 38694(34099to 43075) | 0.1(0.09to0.12) | 13155396(111 22001to155595 59) | 163.63% | 31611(26957to 37021) | -0.12(-0.15to-0.08) | 13014(5888to2 5473) | 163.45% | 31(14to60) | -0.11(-0.15to- 0.08) |
| Ireland | 1939581(1690 200to2190729) | 37.28% | 39484(34581to 44259) | 0.05(0.03to0.0 7) | 1451412(1233 304to1688321) | 31.10% | 27649(23510to 32099) | -0.35(-0.42to-0.27) | 1421(643to273 5) | 30.43% | 27(12to52) | -0.35(-0.42to- 0.27) |
| Israel | 3553516(3104 303to3960461) | 90.17% | 39090(34216to 43614) | 0.1(0.08to0.13) | 2937323(2535 323to3365407) | 71.02% | 31475(27094to 36098) | -0.31(-0.38to-0.25) | 2887(1294to54 66) | 70.31% | 31(14to59) | -0.31(-0.38to- 0.25) |
| Italy | 23354515(208 80076to25838958) | 0.77% | 39425(34703to 43573) | 0.07(0.05to0.0 9) | 17139999(146 64911to19816814) | 2.91% | 25367(21882to 29354) | -0.21(-0.26to-0.17) | 16662(7382to3 2364) | 2.32% | 25(11to48) | -0.2(-0.25to- 0.16) |
| Jamaica | 1101621(9752 07to1235549) | 25.30% | 37135(32644to 41717) | 0.01(0to0.02) | 790142(67003 7to930819) | 25.01% | 26555(22430to 31282) | -0.1(-0.11to- 0.09) | 781(342to1513 ) | 24.34% | 26(12to51) | -0.11(-0.12to- 0.1) |
| Japan | 47745486(429 13066to52931387) | -5.51% | 38669(34485to 42751) | 0.01(0to0.03) | 31752744(273 26008to367764 29) | -0.88% | 23041(19798to 26776) | -0.06(-0.07to- 0.05) | 31038(13790to 60315) | -1.69% | 23(10to44) | -0.06(-0.06to-0.05) |
| Jordan | 4498765(3904 655to5096002) | 231.33% | 37596(33011to 42265) | 0(-0.02to0.02) | 4018305(3429 669to4650482) | 250.96% | 34351(29715to 39678) | 0.09(0.05to0.1 2) | 3992(1765to7836) | 250.18% | 34(15to66) | 0.1(0.06to0.13) |
| Kazakhstan | 7041365(6249 310to7926343) | 11.89% | 38053(33669to 42890) | -0.01(-0.03to0.02) | 5164760(4456 262to5958346) | 14.18% | 28301(24365to 32507) | 0.11(0.07to0.1 5) | 5107(2224to9923) | 14.01% | 28(12to54) | 0.11(0.08to0.1 5) |
| Kenya | 19482493(171 21199to217369 39) | 139.38% | 38554(34612to 42629) | 0.01(-0.01to0.02) | 10498981(880 4743to12296705) | 128.63% | 21545(18494to 25025) | -0.07(-0.1to- 0.03) | 10422(4714to2 0440) | 128.46% | 21(10to41) | -0.06(-0.09to-0.02) |
| Kiribati | 45721(39194to 52130) | 67.13% | 38464(33221to 43753) | 0.05(0.04to0.0 7) | 38282(32623to 44753) | 67.68% | 33709(29028to 39059) | -0.01(-  0.02to0.01) | 38(17to75) | 67.48% | 33(15to65) | -0.01(-0.02to0.01) |
| Kuwait | 1874450(1630 972to2118840) | 164.78% | 39106(33779to 43972) | 0.07(0.05to0.0 8) | 1387729(1154 980to1659699) | 162.64% | 29233(24893to 34290) | -0.11(-0.12to- 0.1) | 1373(609to2671) | 160.75% | 29(13to56) | -0.11(-0.12to- 0.1) |
| Kyrgyzstan | 2468274(2127 160to2826355) | 51.17% | 37938(32907to 43165) | -0.04(-0.07to-0.02) | 2021868(1718 115to2359433) | 53.77% | 31497(26950to 36636) | 0.01(-  0.01to0.03) | 2008(883to383 3) | 53.96% | 31(14to60) | 0.02(0to0.04) |
| Lao People's Democratic Republic | 2934963(2587 402to3236525) | 93.13% | 39914(35184to 43938) | 0.04(0.02to0.0 5) | 1805509(1546 027to2083216) | 81.36% | 25043(21564to 28698) | -0.11(-0.15to- 0.06) | 1794(806to342 7) | 81.43% | 25(11to48) | -0.1(-0.14to-0.05) |
| Latvia | 729798(62720 7to823357) | -27.95% | 38021(32227to 42787) | 0.07(0.05to0.0 9) | 646048(55497 2to749869) | -29.23% | 32413(27602to 37708) | -0.13(-0.17to- 0.09) | 631(277to1210 ) | -29.49% | 32(14to61) | -0.12(-0.16to-0.08) |
| Lebanon | 2023143(1732 299to2290844) | 70.56% | 38795(33266to 43859) | 0.07(0.05to0.0 8) | 1629353(1386 730to1902061) | 65.37% | 31273(26621to 36462) | -0.16(-0.18to- 0.14) | 1604(715to3172) | 64.74% | 31(14to61) | -0.16(-0.18to-0.14) |
| Lesotho | 818964(71055 2to925571) | 25.22% | 37761(32976to 42577) | 0.03(0.02to0.0 4) | 641610(54182 5to744643) | 23.40% | 30885(26281to 35657) | -0.16(-0.17to- 0.15) | 630(282to1212 ) | 22.70% | 30(14to58) | -0.19(-0.19to- 0.18) |
| Liberia | 1793444(1512 792to2029963) | 171.71% | 37969(32837to 42482) | 0.07(0.05to0.0 8) | 1332095(1107 504to1571097) | 170.58% | 29560(25115to 34447) | -0.06(-0.11to- 0.01) | 1314(583to2570) | 172.25% | 29(13to56) | -0.04(-0.09to0.01) |
| Libya | 2779902(2379 993to3127422) | 81.39% | 38340(32767to 43066) | 0.07(0.04to0.1) | 2270303(1920 231to2677356) | 89.30% | 31608(26995to 37056) | 0.03(-  0.01to0.06) | 2241(979to4386) | 87.92% | 31(14to61) | 0.02(-0.01to0.05) |
| Lithuania | 1075374(9385 47to1229039) | -19.43% | 37974(32644to 43238) | 0.25(0.2to0.31) | 988214(85334 9to1145109) | -28.79% | 33593(28947to 38912) | -0.45(-0.57to-0.32) | 965(428to1851 ) | -29.36% | 33(15to64) | -0.44(-0.57to- 0.32) |
| Luxembourg | 251614(22873 7to282470) | 64.41% | 39750(35754to 44401) | 0.12(0.1to0.14) | 190516(16271 5to221341) | 56.06% | 27865(23934to 32391) | -0.15(-0.16to-0.13) | 186(82to358) | 55.69% | 27(12to53) | -0.15(-0.16to-0.13) |
| Madagascar | 9762686(8154 055to11163336 | 155.58% | 37355(31881to 42596) | 0.19(0.14to0.2 4) | 8461586(7226 868to9758425) | 90.10% | 35112(30363to 40243) | -0.55(-0.67to-0.43) | 8392(3635to16 632) | 90.36% | 35(15to68) | -0.54(-0.67to-0.42) |
| Malawi | 6969959(5861 270to7944150) | 107.38% | 38396(33511to 43162) | 0.03(0.02to0.0 5) | 4985775(4153 663to5874267) | 102.43% | 29783(25224to 34750) | -0.09(-0.11to-0.08) | 4943(2189to9504) | 103.31% | 29(13to56) | -0.07(-0.09to-0.06) |
| Malaysia | 12499924(108 37412to13860934) | 88.20% | 37728(32504to 42088) | 0.03(-0.01to0.06) | 6562688(5507 801to7679390) | 80.29% | 19963(16786to 23273) | -0.13(-0.18to-0.08) | 6506(2881to12 760) | 79.74% | 20(9to39) | -0.12(-0.17to-0.07) |
| Maldives | 216721(19385 6to241202) | 172.00% | 39926(35705to 44438) | 0.03(0.02to0.0 5) | 127982(10894 9to149261) | 150.37% | 24414(20808to 28254) | -0.24(-0.26to-0.23) | 127(56to242) | 150.40% | 24(11to47) | -0.23(-0.24to-0.22) |
| Mali | 7513747(6359 326to8550141) | 163.70% | 37772(33275to 42369) | 0.01(0to0.03) | 5308344(4437 860to6241311) | 149.27% | 28495(24239to 33279) | -0.15(-0.16to-0.13) | 5264(2342to10 115) | 150.37% | 28(13to54) | -0.13(-0.14to- 0.11) |
| Malta | 168820(14733 6to188330) | 15.41% | 38850(33854to 43111) | 0.03(0.02to0.0 4) | 140253(11926 4to162369) | 17.65% | 28606(24534to 33177) | -0.24(-0.26to-0.22) | 137(60to258) | 16.70% | 28(13to54) | -0.24(-0.26to-0.22) |
| Marshall  Islands | 22302(19108to 25066) | 36.75% | 38453(33255to 43206) | 0.05(0.02to0.0 7) | 18238(15609to 21264) | 37.70% | 32630(28335to 37613) | -0.05(-0.06to-0.03) | 18(8to35) | 36.48% | 32(14to62) | -0.06(-0.07to-0.04) |
| Mauritania | 1474972(1250 574to1669990) | 110.83% | 37667(32546to 42127) | 0.04(0.03to0.0 6) | 1036190(8684 22to1229456) | 106.30% | 27697(23663to 32309) | -0.09(-0.1to-0.07) | 1029(458to199 6) | 106.50% | 27(12to53) | -0.08(-0.1to-0.07) |
| Mauritius | 524035(46691 7to581275) | 15.45% | 39344(34660to 43654) | 0.02(0to0.03) | 325222(27799 6to376970) | 12.34% | 23919(20360to 27794) | -0.21(-0.22to-0.2) | 319(145to617) | 11.22% | 24(11to45) | -0.21(-0.22to-0.2) |
| Mexico | 47636024(427 39648to52553395) | 48.75% | 36743(32820to 40469) | -0.05(-0.07to-0.03) | 27047990(232 93289to31379215) | 49.62% | 20916(18000to 24249) | -0.23(-0.3to-0.16) | 26683(12062to 51347) | 48.64% | 21(9to40) | -0.24(-0.31to-0.17) |
| Micronesia  (Federated  States of) | 40549(35023to 45664) | 7.52% | 38557(33508to 43234) | 0.05(0.04to0.0 6) | 33637(28671to 39152) | 8.32% | 32920(28440to 38253) | -0.04(-0.07to-0.02) | 33(15to66) | 7.96% | 32(14to63) | -0.05(-0.07to-0.03) |
| Monaco | 13881(12325to 15467) | 17.65% | 38753(34373to 42806) | 0.08(0.06to0.1 1) | 10702(8948to1 2474) | 14.07% | 25824(22112to 30224) | -0.12(-0.15to-0.1) | 10(5to21) | 13.70% | 25(12to50) | -0.13(-0.15to-0.11) |
| Mongolia | 1296294(1112 100to1492450) | 70.56% | 38239(32954to 43888) | 0.05(0.03to0.0 7) | 995424(84867 4to1171831) | 63.42% | 29833(25534to 34997) | -0.14(-0.17to-0.11) | 986(436to1890 ) | 62.70% | 29(13to56) | -0.13(-0.16to-0.1) |
| Montenegro | 226498(19230 2to264631) | 0.89% | 35314(29685to 40961) | 0.07(0.04to0.1 1) | 227095(19238 9to263554) | 0.51% | 34851(29903to 40729) | -0.08(-0.12to-0.05) | 223(97to426) | -0.29% | 34(15to66) | -0.09(-0.12to-0.05) |
| Morocco | 14224928(122 66030to16351351) | 52.54% | 38321(33101to 43911) | 0.07(0.05to0.0 8) | 11836316(101 28256to138326 73) | 48.61% | 32091(27467to 37341) | -0.17(-0.18to-0.16) | 11682(5131to2 3029) | 47.94% | 32(14to62) | -0.17(-0.18to- 0.16) |
| Mozambique | 10582111(887 7716to1208988 6) | 159.96% | 38110(32939to 43260) | 0.24(0.16to0.3 2) | 8151217(6906 485to9569287) | 80.31% | 32320(27874to 37206) | -0.48(-0.61to-0.35) | 8055(3575to15 984) | 80.95% | 32(14to61) | -0.47(-0.6to-0.34) |
| Myanmar | 21219738(188 53094to236030 23) | 38.80% | 37643(33418to 41878) | -0.09(-0.12to-0.06) | 11299861(963 0093to1339678 2) | 28.94% | 20174(17235to 23845) | -0.43(-0.48to-0.38) | 11198(4985to2 2088) | 28.80% | 20(9to39) | -0.41(-0.46to-0.36) |
| Namibia | 928426(80555 2to1046114) | 83.61% | 38389(33559to 43124) | 0.11(0.08to0.1 4) | 676278(57636 8to787491) | 62.23% | 29292(25174to 34103) | -0.45(-0.52to-0.38) | 666(297to1278 ) | 61.69% | 29(13to54) | -0.46(-0.53to-0.38) |
| Nauru | 4110(3558to4623) | 10.52% | 38738(34035to 43157) | 0.04(0.03to0.0 6) | 3134(2633to36 90) | 12.72% | 31346(26856to 36542) | 0.1(0.03to0.16) | 3(1to6) | 12.53% | 31(14to60) | 0.09(0.02to0.16) |
| Nepal | 12484465(108 63756to139029 03) | 73.21% | 39842(34779to 44289) | -0.02(-0.05to0.01) | 8631039(7389 313to10010453 ) | 78.16% | 28097(24342to 32542) | 0(-0.05to0.06) | 8520(3822to16 353) | 78.25% | 28(12to53) | 0.03(-0.03to0.09) |
| Netherlands | 6655812(5844 591to7543087) | 10.14% | 39032(33982to 44191) | 0.02(0.01to0.0 3) | 4755359(3958 773to5562274) | 10.60% | 25340(21494to 29741) | -0.19(-0.22to-0.17) | 4651(2079to90 30) | 9.84% | 25(11to48) | -0.19(-0.21to-0.17) |
| New Zealand | 1741135(1541 621to1937498) | 28.07% | 38831(33990to 43304) | -0.03(-0.1to0.04) | 1107404(9385 22to1286262) | 32.28% | 23512(20001to 27432) | 0.05(-0.03to0.14) | 1080(482to209 7) | 31.86% | 23(10to44) | 0.06(-0.02to0.14) |
| Nicaragua | 2389134(2076 385to2697825) | 85.39% | 35367(30943to 39757) | 0.03(0.01to0.0 4) | 1822518(1532 299to2134951) | 83.58% | 27916(23575to 32720) | -0.15(-0.17to-0.13) | 1806(805to360 0) | 83.29% | 28(12to55) | -0.14(-0.16to-0.12) |
| Niger | 7663865(6357 207to8888312) | 194.77% | 37903(32834to 43076) | 0.24(0.18to0.3 1) | 5380028(4449 378to6426299) | 185.92% | 28642(24317to 33449) | -0.39(-0.53to-0.25) | 5348(2400to10 293) | 186.92% | 28(13to54) | -0.39(-0.53to-0.24) |
| Nigeria | 78971111(684 09173to886485 04) | 149.16% | 38764(34753to 42736) | 0.04(0.03to0.0 6) | 43271487(361 88009to509032 86) | 145.85% | 22129(19004to 25695) | -0.02(-0.03to- 0.01) | 42875(19282to 82853) | 146.37% | 22(10to42) | -0.02(-0.03to0) |
| Niue | 651(557to741) | -24.88% | 38934(33399to 44288) | 0.1(0.06to0.13) | 537(458to627) | -24.45% | 31068(26523to 36242) | -0.07(-0.09to-0.05) | 1(0to1) | -25.00% | 31(14to59) | -0.08(-0.1to-0.06) |
| North  Macedonia | 802909(68357 7to912633) | 12.61% | 35528(29847to 40585) | 0.1(0.09to0.12) | 810397(68765 6to932188) | 9.97% | 35286(30362to 40758) | -0.06(-0.09to-0.03) | 797(352to1558 ) | 9.30% | 35(15to67) | -0.06(-0.09to-0.03) |
| Northern  Mariana | 17154(14972to 19320) | -10.38% | 38980(34104to 43847) | 0(-0.01to0.02) | 13604(11521to 15889) | -0.09% | 29796(25466to 34839) | 0.05(0.03to0.0 8) | 13(6to26) | -1.34% | 29(13to58) | 0.04(0.02to0.0 7) |
| Norway | 2054116(1820 929to2275132) | 22.36% | 38475(33798to 42648) | -0.03(-0.06to0.01) | 1776325(1537 312to2031711) | 37.19% | 29478(25533to 33794) | 0.25(0.14to0.3 5) | 1730(775to3288) | 36.74% | 29(13to56) | 0.24(0.14to0.3 5) |
| Oman | 1952118(1718 483to2208964) | 173.28% | 38672(34375to 43395) | 0.09(0.07to0.1) | 1438085(1199 255to1718609) | 162.49% | 29854(25398to 34901) | -0.12(-0.13to- 0.1) | 1429(620to2782) | 161.87% | 29(13to57) | -0.12(-0.13to-0.1) |
| Pakistan | 89463535(784 81413to992898 26) | 118.64% | 40540(36234to 44764) | 0.08(0.07to0.1) | 52232324(443 08231to603245 57) | 97.31% | 25439(21953to 29184) | -0.18(-0.24to-0.12) | 51686(22946to 99782) | 97.11% | 25(11to48) | -0.18(-0.24to-0.12) |
| Palau | 7311(6472to82 89) | 18.58% | 39148(34434to 44120) | 0.06(0.03to0.0 9) | 5945(5061to69 92) | 23.80% | 30844(26410to 35868) | -0.03(-0.05to-0.01) | 6(3to11) | 22.53% | 30(13to59) | -0.04(-0.06to-0.02) |
| Palestine | 1935237(1696 312to2173112) | 170.05% | 38693(34502to 43577) | 0.1(0.06to0.14) | 1402291(1218 543to1597150) | 164.98% | 28971(25199to 33012) | -0.18(-0.23to-0.13) | 1389(608to2642) | 164.44% | 28(13to54) | -0.19(-0.24to-0.14) |
| Panama | 1517921(1309 662to1718315) | 80.39% | 36098(31137to 40870) | 0.15(0.14to0.1 7) | 1235575(1056 021to1444531) | 58.94% | 29403(25142to 34341) | -0.42(-0.47to-0.37) | 1220(535to2345) | 58.12% | 29(13to56) | -0.43(-0.48to-0.37) |
| Papua New Guinea | 3755433(3187 401to4325098) | 147.88% | 38725(33374to 44242) | 0.05(0.03to0.0 6) | 3019615(2559 178to3520576) | 140.72% | 32899(28274to 37915) | -0.06(-0.08to-0.04) | 2986(1333to5824) | 140.56% | 32(14to62) | -0.05(-0.07to-0.03) |
| Paraguay | 2701264(2406 687to2988571) | 87.86% | 37505(33436to 41461) | 0.04(0.02to0.0 6) | 1679732(1411 070to1961500) | 79.20% | 23611(19995to 27508) | -0.14(-0.16to-0.11) | 1663(735to3189) | 78.73% | 23(10to44) | -0.13(-0.16to-0.11) |
| Peru | 12491369(109 32637to14177256) | 64.22% | 35959(31406to 40863) | 0.06(0.05to0.0 7) | 11790777(100 84099to13556443) | 60.43% | 34344(29468to 39479) | -0.11(-0.13to-0.1) | 11677(5102to2 2716) | 59.95% | 34(15to66) | -0.1(-0.12to-0.09) |
| Philippines | 45924543(404 05951to50635240) | 85.43% | 40588(35852to 44651) | 0.02(0.01to0.0 3) | 29206277(249 97610to337263 86) | 86.23% | 26596(22902to 30507) | -0.11(-0.22to0) | 28919(13140to 56485) | 86.12% | 26(12to51) | -0.1(-0.21to0.01) |
| Poland | 15242017(136 98762to16930008) | 1.90% | 38509(33977to 42860) | 0.08(0.04to0.1 2) | 10886010(928 7372to1263023 4) | 0.87% | 26657(22815to 31112) | -0.12(-0.14to-0.11) | 10669(4795to2 0703) | 0.58% | 26(12to51) | -0.11(-0.13to-0.09) |
| Portugal | 3980939(3556 045to4432900) | 0.41% | 37884(33434to 42195) | -0.09(-0.14to-0.04) | 2817195(2376 009to3302749) | 2.01% | 24016(20480to 28146) | -0.5(-0.6to-0.4) | 2749(1235to5301) | 1.56% | 24(11to45) | -0.5(-0.59to-0.4) |
| Puerto Rico | 1318165(1164 215to1474858) | -3.15% | 37619(33305to 42050) | 0.07(0.04to0.1 1) | 883842(74751 7to1032946) | -3.05% | 24260(20485to 28548) | -0.06(-0.09to-0.02) | 866(385to1678) | -3.94% | 24(11to46) | -0.06(-0.09to-0.02) |
| Qatar | 1333100(1175 396to1509427) | 624.73% | 40027(35332to 44897) | 0.14(0.1to0.17) | 915021(74653 5to1107740) | 589.92% | 28618(23843to 33777) | -0.13(-0.16to- 0.1) | 909(398to1747 ) | 588.38% | 28(13to55) | -0.14(-0.17to-0.11) |
| Republic of  Korea | 20965618(186 36283to23830760) | 17.30% | 37890(33322to 42566) | -0.09(-0.14to-0.04) | 14991454(126 87088to175288 46) | 19.15% | 25187(21465to 29640) | -0.43(-0.52to-0.34) | 14749(6631to2 9318) | 18.31% | 25(11to49) | -0.42(-0.51to-0.33) |
| Republic of  Moldova | 1437192(1228773to1666799) | -13.44% | 37423(31993to 43225) | 0.04(0.02to0.06) | 1383565(1175 958to1594748) | -9.84% | 34836(29924to 40328) | 0.01(-0.05to0.07) | 1357(609to2645) | -10.19% | 34(15to68) | 0.02(-0.05to0.08) |
| Romania | 6946996(6049 577to8029801) | -13.48% | 34945(30115to 40218) | 0.19(0.16to0.21) | 7557765(6516 963to8717684) | -23.60% | 37497(32404to 43075) | -0.37(-0.42to-0.32) | 7405(3247to13 957) | -24.00% | 37(16to71) | -0.37(-0.42to-0.32) |
| Russian  Federation | 58481755(51907053to65299438) | -2.53% | 39667(34826to 44104) | 0.06(0.04to0.0 9) | 45329444(388 42827to52339032) | -5.31% | 29056(24993to 33483) | -0.18(-0.24to-0.12) | 44293(19822to 85387) | -5.65% | 29(13to56) | -0.17(-0.24to-0.11) |
| Rwanda | 4889965(4216 727to5536001) | 94.40% | 38580(33744to 43575) | 0.06(0.04to0.0 8) | 3468417(2918 633to4066976) | 90.68% | 29023(24565to 33685) | -0.16(-0.2to- 0.12) | 3430(1539to66 74) | 90.87% | 29(13to56) | -0.14(-0.18to-0.1) |
| Saint Kitts and Nevis | 23146(20390to 25895) | 51.52% | 37349(32479to 41894) | 0.02(0to0.03) | 16005(13576to 18955) | 49.09% | 25273(21575to 29818) | -0.19(-0.2to- 0.18) | 16(7to30) | 48.46% | 25(11to48) | -0.19(-0.2to-0.18) |
| Saint Lucia | 68532(60007to 76995) | 36.01% | 37455(32385to 42047) | 0.03(0.01to0.0 6) | 48933(41749to 57883) | 37.33% | 26123(22236to 30963) | -0.14(-0.16to-0.13) | 48(22to93) | 36.30% | 26(12to50) | -0.14(-0.16to-0.12) |
| Saint Vincent and the Grenadines | 43580(38030to 49290) | 12.29% | 37590(32627to 42443) | 0.17(0.12to0.2 1) | 37662(32406to 43741) | -8.32% | 31789(27227to 36915) | -0.64(-0.76to-0.51) | 37(16to73) | -9.33% | 31(14to61) | -0.65(-0.78to-0.52) |
| Samoa | 82699(69994to 93216) | 33.37% | 38589(33079to 43229) | 0.07(0.06to0.0 8) | 66529(56344to 77778) | 31.36% | 32018(27173to 37199) | -0.1(-0.12to-0.08) | 66(30to131) | 30.91% | 32(14to62) | -0.11(-0.13to-0.08) |
| San Marino | 12962(11344to 14756) | 38.82% | 39096(33935to 44178) | 0.06(0.04to0.0 9) | 10228(8607to1 2052) | 39.44% | 28051(23932to 32929) | -0.13(-0.18to-0.08) | 10(4to19) | 38.62% | 28(12to51) | -0.14(-0.19to-0.08) |
| Sao Tome and Principe | 77494(65751to 87992) | 87.63% | 37262(32037to 41697) | 0.07(0.05to0.1) | 54813(45953to 65133) | 85.73% | 27421(23119to 32256) | -0.08(-0.11to-0.05) | 54(24to106) | 85.81% | 27(12to52) | -0.08(-0.11to-0.05) |
| Saudi Arabia | 15023554(13144017to16977050) | 150.76% | 38040(33558to 42628) | 0.03(-0.02to0.08) | 12966618(110 49335to15231082) | 159.99% | 34054(29721to 39040) | -0.22(-0.33to-0.1) | 12835(5513to2 4907) | 158.65% | 34(15to64) | -0.22(-0.34to-0.11) |
| Senegal | 5368432(4571 313to6187937) | 115.78% | 36549(31633to 41857) | 0.1(0.07to0.13) | 5003881(4287 966to5724349) | 122.21% | 35776(31026to 40717) | 0.12(0.07to0.1 6) | 4952(2193to97 09) | 122.55% | 35(15to69) | 0.13(0.08to0.1 7) |
| Serbia | 3161316(2761 651to3578944) | -4.48% | 34958(30179to 39951) | -0.45(-0.78to-0.11) | 3409797(2928 663to3937322) | -9.65% | 37154(32334to 42719) | 0.48(0.08to0.8 7) | 3340(1454to63 06) | -10.20% | 37(16to70) | 0.47(0.08to0.8 7) |
| Seychelles | 41757(37440to 46070) | 43.84% | 39514(35482to 43655) | 0.07(0.04to0.0 9) | 25029(21288to 29131) | 37.65% | 23677(20070to 27355) | -0.12(-0.14to-0.09) | 25(11to48) | 36.82% | 23(11to46) | -0.12(-0.15to-0.09) |
| Sierra Leone | 2990664(2577 210to3368492) | 161.46% | 37157(32549to 41355) | 0.28(0.21to0.3 5) | 2299624(1911 516to2715177) | 100.10% | 30209(25695to 35365) | -0.59(-0.7to- 0.48) | 2281(987to439 9) | 101.10% | 30(13to57) | -0.58(-0.69to-0.47) |
| Singapore | 2245085(1957154  to2514634) | 77.42% | 37730(33049to 42213) | 0(-0.02to0.03) | 1530625(1277 537to1805818) | 78.50% | 24280(20543to 28504) | -0.24(-0.26to-0.22) | 1513(657to292 2) | 77.70% | 24(11to45) | -0.23(-0.25to-0.21) |
| Slovakia | 2031912(1761519  to2337907) | 7.83% | 35971(30641to 41063) | 0.07(0.05to0.0 8) | 1960110(1663 424to2264851) | 2.22% | 34294(29413to 39586) | -0.19(-0.21to-0.16) | 1921(821to363 2) | 1.65% | 34(15to64) | -0.18(-0.21to-0.15) |
| Slovenia | 773975(668961to870344) | 8.67% | 36557(31438to 41253) | 0.27(0.23to0.3 2) | 684313(57825 6to803946) | -6.19% | 32266(27574to 37791) | -0.36(-0.41to-0.31) | 669(292to1285 ) | -6.89% | 32(14to62) | -0.35(-0.4to-0.3) |
| Solomon  Islands | 245511(21109 6to279386) | 102.75% | 38260(33634to 42929) | 0.08(0.05to0.1) | 202482(17129 1to236655) | 98.17% | 33429(28887to 38657) | -0.01(-0.03to0.01) | 201(89to397) | 97.56% | 33(15to64) | -0.02(-0.04to0) |
| Somalia | 7209802(6046 355to8178815) | 190.90% | 37902(32996to 42370) | 0(0to0.01) | 5600998(4678 107to6618275) | 191.01% | 32404(27788to 37523) | 0.02(-0.01to0.05) | 5539(2477to10 825) | 191.53% | 32(14to60) | 0.03(0to0.05) |
| South Africa | 22755044(201 21597to25096464) | 59.88% | 39492(34802to 43621) | 0.06(0.04to0.0 7) | 14068467(120 41717to16420740) | 60.10% | 24528(21018to 28563) | -0.05(-0.08to-0.01) | 13806(6220to2 6880) | 59.20% | 24(11to46) | -0.05(-0.09to-0.02) |
| South Sudan | 3385928(2882 717to3858455) | 58.41% | 38736(34037to 43459) | 0.02(0to0.04) | 2209294(1852 206to2620077) | 58.28% | 26928(23067to 31152) | -0.05(-0.07to-0.03) | 2180(973to425 0) | 58.30% | 26(12to51) | -0.04(-0.06to-0.03) |
| Spain | 17848125(157 76897to19986271) | 17.68% | 39161(34748to 43623) | 0.17(0.13to0.2) | 14978184(128 19514to172133 43) | 9.07% | 29110(25144to 33621) | -0.42(-0.51to-0.34) | 14631(6505to2 8514) | 8.32% | 29(13to56) | -0.42(-0.51to-0.34) |
| Sri Lanka | 8861231(7868 373to9894350) | 26.84% | 39598(35053to 44189) | 0.01(-0.01to0.03) | 5571213(4746 998to6505226) | 21.18% | 24606(20952to 28726) | -0.25(-0.27to-0.23) | 5490(2492to10 549) | 20.32% | 24(11to46) | -0.25(-0.27to-0.23) |
| Sudan | 15411112(132 13891to17613353) | 120.17% | 37854(33198to 42774) | 0.07(0.05to0.0 9) | 12529969(102 63986to14722291) | 108.42% | 32352(27371to 37744) | -0.14(-0.16to-0.11) | 12451(5443to2 4531) | 108.64% | 32(14to63) | -0.13(-0.16to-0.11) |
| Suriname | 205420(18109 2to232546) | 47.30% | 35089(30811to 39641) | -0.04(-0.06to-0.02) | 115880(98376t o138001) | 46.02% | 19669(16633to 23404) | -0.21(-0.25to-0.16) | 114(51to218) | 45.49% | 19(9to37) | -0.21(-0.26to-0.17) |
| Sweden | 3921470(3462 410to4361362) | 21.83% | 39069(34228to 43368) | 0.18(0.13to0.2 3) | 3479793(3021 591to3974219) | 20.23% | 30632(26345to 35161) | -0.12(-0.13to-0.11) | 3395(1526to65 30) | 19.75% | 30(14to58) | -0.12(-0.13to-0.12) |
| Switzerland | 3311783(2937 056to3685788) | 24.29% | 37970(33249to 42195) | 0.09(0.06to0.1 2) | 3357146(2895 369to3864485) | 23.64% | 34086(29411to 39251) | -0.14(-0.19to-0.09) | 3272(1457to61 30) | 23.42% | 34(15to65) | -0.13(-0.18to-0.08) |
| Syrian Arab Republic | 5636957(4823 025to6333501) | 24.85% | 37837(32493to 42178) | 0.07(0.06to0.0 9) | 4934204(4150 771to5793579) | 29.14% | 32924(28133to 38405) | -0.1(-0.13to-0.07) | 4864(2157to94 64) | 28.00% | 32(14to63) | -0.11(-0.13to-0.08) |
| Taiwan (Province of China) | 9262106(8271 960to10240527) | 11.80% | 37896(33708to 42024) | -0.02(-0.05to0.01) | 5141576(4357 172to5994389) | 14.69% | 20298(17376to 23493) | -0.1(-0.13to-0.06) | 5083(2252to10 109) | 13.85% | 20(9to39) | -0.09(-0.13to-0.06) |
| Tajikistan | 3532078(3039 604to3978619) | 89.57% | 37555(32791to 41867) | -0.03(-0.04to-0.02) | 2901549(2458 038to3407988) | 96.18% | 31357(26842to 36655) | 0.04(-0.02to0.1) | 2884(1244to54 87) | 96.05% | 31(14to59) | 0.04(-0.02to0.1) |
| Thailand | 28933510(258 65315to32102081) | 22.71% | 39596(35246to 43934) | 0.05(0.04to0.0 7) | 18113076(155 29364to21105417) | 19.54% | 24248(20776to 28238) | -0.19(-0.2to-0.18) | 17858(7913to3 3759) | 18.73% | 24(11to46) | -0.18(-0.19to- 0.17) |
| Timor-Leste | 515217(44627 1to579031) | 78.19% | 39640(34800to 44546) | 0.01(-0.02to0.04) | 410891(34875 5to478979) | 87.26% | 31376(26880to 36186) | -0.04(-0.1to0.02) | 408(178to796) | 87.28% | 31(14to60) | -0.02(-0.07to0.04) |
| Togo | 2908075(2456 474to3322298) | 136.85% | 37862(32777to 42962) | 0.05(0.05to0.0 6) | 2111107(1763 977to2493796) | 137.37% | 28806(24391to 33818) | -0.01(-0.02to0) | 2092(930to402 9) | 137.45% | 28(13to55) | 0(-0.01to0.01) |
| Tokelau | 528(453to598) | -9.47% | 38864(33577to 43993) | 0.1(0.07to0.12) | 430(367to502) | -13.89% | 31705(27130to 36968) | -0.14(-0.16to-0.13) | 0(0to1) | -14.08% | 31(14to61) | -0.15(-0.16to-0.13) |
| Tonga | 38983(33919to 44203) | 8.05% | 38668(34159to 43539) | 0.06(0.05to0.0 7) | 31768(27083to 36911) | 6.93% | 32220(27864to 37329) | -0.06(-0.07to-0.05) | 31(14to61) | 6.52% | 32(14to61) | -0.06(-0.07to-0.06) |
| Trinidad and  Tobago | 533616(47637 7to593675) | 17.61% | 37735(33272to 42070) | 0.11(0.09to0.1 2) | 368270(31355 1to431174) | 16.04% | 25373(21624to 29843) | -0.22(-0.24to-0.2) | 362(162to698) | 15.06% | 25(11to49) | -0.22(-0.24to-0.2) |
| Tunisia | 4536846(3998 227to5138936) | 44.43% | 38267(33632to 43231) | 0.04(0.01to0.0 6) | 3720081(3169 537to4366573) | 42.65% | 30953(26529to 36198) | -0.02(-0.11to0.07) | 3670(1619to71 14) | 41.47% | 31(13to59) | -0.02(-0.11to0.07) |
| Turkey | 32659193(286 67576to370707 56) | 48.82% | 38143(33377to 43357) | 0.14(0.09to0.1 9) | 29494422(252 83344to34082399) | 45.79% | 34206(29401to 39477) | -0.15(-0.26to-0.04) | 29092(12887to 57225) | 44.96% | 34(15to66) | -0.15(-0.25to- 0.04) |
| Turkmenistan | 1971348(1740 251to2226302) | 47.81% | 38632(34233to 43534) | 0.05(0.03to0.0 7) | 1475553(1247 851to1743381) | 43.04% | 29147(24736to 34371) | -0.13(-0.18to-0.08) | 1464(649to2838) | 42.56% | 29(13to56) | -0.13(-0.18to-0.08) |
| Tuvalu | 4626(3967to5232) | 34.25% | 38300(32826to 43332) | 0.01(-0.01to0.03) | 3876(3327to44 78) | 29.92% | 32533(27996to 37460) | -0.12(-0.13to-0.11) | 4(2to8) | 29.58% | 32(14to64) | -0.13(-0.14to-0.12) |
| Uganda | 14694124(125 11792to16814830) | 152.03% | 37908(33253to 42243) | 0(-0.02to0.02) | 8468594(6994 290to10054808 ) | 141.77% | 23348(19874to 27500) | -0.13(-0.17to- 0.1) | 8406(3776to16 147) | 143.51% | 23(10to44) | -0.1(-0.14to-0.07) |
| Ukraine | 17618487(155 28609to19996839) | -16.23% | 39318(34429to 44137) | 0.01(-0.01to0.03) | 13579832(115 62183to15764436) | -13.10% | 28481(24355to 33093) | -0.01(-0.04to0.02) | 13281(5897to2 5824) | -13.33% | 28(13to55) | 0(-0.04to0.03) |
| United Arab  Emirates | 3960903(3423 024to4547694) | 420.63% | 38524(34080to 43136) | 0.01(-0.01to0.02) | 3014656(2441 600to3722017) | 458.10% | 28840(24587to 33544) | -0.01(-0.03to0) | 2985(1309to57 68) | 454.88% | 28(13to56) | -0.02(-0.03to0) |
| United  Kingdom | 26567606(238 94782to29225955) | 15.57% | 39582(35057to 43636) | 0.04(0.03to0.0 5) | 19349507(167 20902to22169661) | 22.35% | 26402(22859to 30399) | -0.01(-0.09to0.07) | 18831(8295to3 6795) | 21.86% | 26(12to51) | -0.01(-0.08to0.07) |
| United Republic  of Tanzania | 20609689(173 30273to235727 05) | 129.56% | 38172(33241to 43018) | 0.09(0.05to0.1 2) | 15527694(129 66613to18332930) | 134.43% | 31034(26424to 36196) | -0.51(-0.72to-0.29) | 15371(6913to2 9811) | 135.97% | 31(14to59) | -0.49(-0.7to-0.27) |
| United States of America | 129828790(117712295to141908411) | 26.33% | 39112(35073to 42965) | -0.04(-0.06to-0.01) | 75034853(649 16762to869903 73) | 24.12% | 21722(18749to 25090) | -0.13(-0.21to-0.05) | 72797(32142to 139849) | 23.05% | 21(10to41) | -0.13(-0.21to-0.05) |
| United States Virgin Islands | 38877(34378to 43267) | -2.11% | 38548(33886to 42423) | 0.11(0.08to0.1 3) | 26210(22073to 31001) | -5.26% | 25013(21347to 29550) | -0.16(-0.18to-0.13) | 26(12to50) | -6.20% | 25(11to48) | -0.16(-0.19to-0.14) |
| Uruguay | 1287015(1123 383to1465853) | 14.54% | 37315(32389to 42529) | 0.1(0.07to0.13) | 1206317(1037 650to1388589) | 4.34% | 33275(28778to 38491) | -0.28(-0.29to-0.27) | 1183(522to224 8) | 3.84% | 33(15to63) | -0.28(-0.29to-0.27) |
| Uzbekistan | 13004418(111 69066to14790791) | 76.30% | 38058(32704to 43090) | 0.03(0to0.06) | 10251135(865 5566to11984685) | 72.97% | 30320(25946to 35268) | -0.08(-0.11to-0.06) | 10172(4494to1 9602) | 72.79% | 30(13to58) | -0.08(-0.1to-0.05) |
| Vanuatu | 111920(94993t o127002) | 105.39% | 38284(33205to 43250) | 0.06(0.05to0.0 7) | 92000(77869to 107500) | 104.71% | 32976(28279to 38267) | -0.04(-0.05to-0.02) | 91(40to183) | 104.19% | 32(14to63) | -0.04(-0.05to-0.03) |
| Venezuela  (Bolivarian  Republic of) | 10174300(901 4769to11439152) | 51.13% | 35585(31357to 40092) | 0.03(0.01to0.0 6) | 7816668(6578 892to9199942) | 57.58% | 27042(22749to 31678) | -0.09(-0.12to-0.06) | 7713(3404to14 719) | 56.41% | 27(12to51) | -0.09(-0.12to-0.06) |
| Viet Nam | 40024552(346 43745to44669601) | 52.61% | 39514(34328to 44246) | 0.12(0.07to0.1 7) | 25052422(212 37416to29006494) | 39.92% | 24949(21096to 29016) | -0.78(-0.95to-0.62) | 24848(11286to 48066) | 39.59% | 25(11to47) | -0.78(-0.95to-0.61) |
| Yemen | 11693422(100 32797to13365384) | 157.46% | 37664(33061to 42331) | 0.06(0.04to0.0 7) | 9944327(8368 784to11651721 ) | 153.44% | 33826(29186to 39121) | -0.09(-0.13to-0.06) | 9843(4344to19 222) | 153.09% | 33(15to64) | -0.09(-0.12to-0.06) |
| Zambia | 6825558(5811 993to7635199) | 146.06% | 38448(33617to 42172) | 0(-0.01to0.02) | 4315764(3543 400to5139619) | 114.74% | 25952(21724to 30567) | -0.27(-0.31to-0.24) | 4268(1914to8257) | 114.63% | 25(11to49) | -0.27(-0.31to-0.23) |
| Zimbabwe | 5593262(4792 213to6300075) | 52.53% | 37776(32922to 42122) | 0.03(0.02to0.0 4) | 3622155(3022 427to4293135) | 60.97% | 25891(21899to 30476) | 0.16(0.12to0.2) | 3584(1652to7014) | 60.59% | 25(12to49) | 0.16(0.12to0.1 9) |

EAPC estimated annual percentage change; ASIR age-standardized incidence rate; ASPR age-standardized prevalence rate; ASYR age-standardized YLDs rate; CI confidence interval

*All data reported as number or rate (95% uncertainty interval)

| **TableS2:** **Incident** **cases,** **prevalent** **cases,** **and** **years** **lived** **with** **disabilities** **(YLDs)** **for** **caries** **in** **deciduous** **teeth** **in** **2019** **for** **both** **sexes** **and** **the** **estimated** **annual** **percentage** **changes** **(EAPCs)** **from** **1990** **to** **2019** **by** **204** **countries** **and** **territories.** **EAPCs,** **estimated** **annual** **percentage** **changes** | | | | | | | | | | | | |
| --- | --- | --- | --- | --- | --- | --- | --- | --- | --- | --- | --- | --- |
|  | **Incidence** **(95%** **UI)** | | | | **Prevalence** **(95%** **UI)** | | | | **YLDs** **(95%** **UI)** | | | |
|  | **Counts (2019)** | **Change(%)**  **(1990-2019)** | **ASIR (1/105)**  **(2019)** | **EAPC of ASIR (95% CI)** | **Counts (2019)** | **Change(%)**  **(1990-2019)** | **ASPR (1/105)**  **(2019)** | **EAPC of ASPR (95% CI)** | **Counts (2019)** | **Change(%)**  **(1990-2019)** | **ASYR (1/105)**  **(2019)** | **EAPC of ASYR (95% CI)** |
| Afghanistan | 10490748(7392497to13804177) | 252.15% | 17138(11914to 22526) | 0(-0.01to0) | 5120200(3993 603to6266089) | 252.58% | 8173(6418to10 036) | 0(-0.02to0.02) | 1961(847to409 0) | 253.54% | 3(1to7) | 0.01(-0.01to0.03) |
| Albania | 287001(20423 8to375522) | -58.53% | 17764(12624to 23167) | 0.01(0to0.02) | 140100(11366 9to165497) | -59.14% | 8688(7041to10 262) | -0.02(-0.03to-0.01) | 54(23to109) | -59.07% | 3(1to7) | -0.01(-0.03to0) |
| Algeria | 7191621(4928 715to9529730) | 8.37% | 17239(11792to 22850) | 0(-0.01to0.01) | 3309294(2565 766to4079758) | 9.36% | 7849(6096to96 57) | -0.02(-0.03to-0.01) | 1271(547to257 4) | 9.41% | 3(1to6) | -0.01(-0.02to0) |
| American  Samoa | 9788(6727to12 798) | -14.54% | 17413(12193to 22556) | 0.01(0to0.02) | 4529(3605to54 36) | -19.38% | 8257(6518to99 35) | 0.01(0to0.02) | 2(1to4) | -19.44% | 3(1to7) | 0.01(0.01to0.0 2) |
| Andorra | 5059(2923to76 02) | 14.85% | 13758(7809to2 0231) | 0.02(0to0.03) | 1837(1295to24 29) | 13.42% | 5185(3609to68 61) | 0.03(0.02to0.0 4) | 1(0to1) | 13.59% | 2(1to4) | 0.03(0.02to0.0 4) |
| Angola | 7977069(4758 496to10979724 | 201.67% | 15825(9407to2 1912) | -0.03(-0.04to-0.02) | 3458324(2542 552to4543504) | 190.87% | 6792(5000to89 02) | -0.07(-0.09to-0.05) | 1323(552to277 3) | 190.50% | 3(1to5) | -0.07(-0.09to-0.05) |
| Antigua and  Barbuda | 9865(6529to13 047) | -13.01% | 17291(11663to 22616) | -0.01(-0.02to0.01) | 4443(3411to54 04) | -15.82% | 8041(6154to97 79) | -0.04(-0.05to-0.02) | 2(1to4) | -15.54% | 3(1to6) | -0.03(-0.05to-0.02) |
| Argentina | 5948029(3778 419to8082461) | 6.89% | 16011(10232to 21715) | 0.01(-0.02to0.03) | 2699256(2049 206to3457495) | 6.34% | 7367(5571to94 51) | 0.11(-0.06to0.27) | 1040(447to222 1) | 6.77% | 3(1to6) | 0.11(-0.05to0.27) |
| Armenia | 392119(27319 5to511480) | -40.70% | 17888(12503to 23329) | 0.01(-0.01to0.03) | 185106(14665 2to218224) | -43.12% | 8537(6768to10 059) | -0.03(-0.04to-0.02) | 71(31to139) | -43.01% | 3(1to6) | -0.02(-0.04to-0.01) |
| Australia | 2319479(1414 432to3260520) | 22.42% | 14511(8844to  20295) | 1.04(0.31to1.7 8) | 1110027(8210 29to1430067) | 21.64% | 7020(5155to90 52) | 1.49(1.03to1.9 5) | 427(178to874) | 21.60% | 3(1to6) | 1.48(1.03to1.9 4) |
| Austria | 610619(35303 9to890150) | -6.11% | 13712(7914to  19914) | 0.01(-0.01to0.03) | 233961(16668 8to307999) | -6.09% | 5267(3752to69 33) | 0.02(0to0.04) | 90(37to184) | -5.97% | 2(1to4) | 0.02(0to0.04) |
| Azerbaijan | 1507380(1041 864to1989217) | -2.74% | 18045(12508to 23762) | 0.01(0to0.03) | 690477(54944 6to818625) | -7.29% | 8431(6695to99 71) | -0.02(-0.04to-0.01) | 266(115to542) | -7.03% | 3(1to7) | -0.02(-0.03to-0.01) |
| Bahamas | 45965(29841to 63046) | -6.47% | 17307(11612to 23323) | 0.01(-0.01to0.02) | 20092(15790to 24545) | -8.58% | 8000(6243to97 57) | -0.03(-0.04to-0.02) | 8(3to16) | -8.76% | 3(1to6) | -0.03(-0.04to-0.02) |
| Bahrain | 141665(97358t o189156) | 38.79% | 17298(12031to 23005) | 0(-0.01to0.01) | 61179(48338to 75587) | 32.42% | 7709(6074to95 69) | -0.02(-0.03to-0.01) | 23(10to49) | 32.41% | 3(1to6) | -0.02(-0.04to-0.01) |
| Bangladesh | 26550889(181 57337to347206 38) | -12.85% | 17380(12026to 22518) | -0.02(-0.03to0) | 11653294(915 6005to1444148 2) | -18.10% | 7835(6136to96 81) | -0.06(-0.07to-0.05) | 4474(1899to96 66) | -17.76% | 3(1to6) | -0.04(-0.05to-0.03) |
| Barbados | 28891(19169to 38817) | -22.38% | 17349(11810to 23053) | -0.01(-0.02to0) | 12943(10140to 15788) | -23.95% | 8073(6268to98 31) | -0.01(-0.02to0) | 5(2to10) | -23.88% | 3(1to6) | -0.02(-0.03to0) |
| Belarus | 1055597(7415 64to1351793) | -30.86% | 17798(12569to 22797) | 0.03(0.02to0.0 4) | 509266(40854 4to602540) | -31.83% | 8625(6917to10 192) | -0.05(-0.06to-0.04) | 196(86to406) | -31.70% | 3(1to7) | -0.05(-0.06to-0.03) |
| Belgium | 955855(55580 9to1374227) | 28.33% | 14131(8176to2 0299) | 3.19(2.14to4.2 4) | 363146(26051 2to476620) | 38.32% | 5431(3873to71 34) | 1.7(1.05to2.36) | 140(57to286) | 38.41% | 2(1to4) | 1.71(1.06to2.3 6) |
| Belize | 71830(46769to 95875) | 43.79% | 17294(11531to 22826) | -0.01(-0.03to0) | 32801(25551to 39475) | 38.38% | 8140(6323to97 86) | -0.05(-0.05to-0.04) | 13(6to27) | 38.60% | 3(1to7) | -0.04(-0.05to-0.03) |
| Benin | 3306167(2123 766to4448700) | 132.60% | 16140(10402to 21860) | 0(-0.01to0.01) | 1492166(1078 031to1919286) | 126.43% | 7101(5190to91 01) | -0.06(-0.07to-0.05) | 570(234to1192 ) | 127.01% | 3(1to6) | -0.05(-0.06to-0.05) |
| Bermuda | 5238(3510to70 10) | -27.72% | 17496(11947to 23284) | 0.03(0.01to0.0 4) | 2294(1798to28 08) | -32.54% | 7965(6206to97 28) | -0.06(-0.07to-0.05) | 1(0to2) | -32.55% | 3(1to6) | -0.06(-0.08to-0.05) |
| Bhutan | 114223(77509t o149984) | -24.61% | 17294(11770to 22615) | 0(-0.02to0.01) | 50718(39128to 62323) | -28.53% | 7711(5940to94 82) | -0.1(-0.11to- 0.09) | 19(8to41) | -28.25% | 3(1to6) | -0.09(-0.1to- 0.08) |
| Bolivia (Plurinational State of) | 2365098(1605 399to3168942) | 44.38% | 16886(11284to 22722) | -0.04(-0.06to-0.03) | 1157423(8898 16to1408588) | 43.11% | 8063(6231to98 04) | -0.06(-0.08to-0.04) | 443(190to927) | 43.22% | 3(1to7) | -0.06(-0.08to-0.04) |
| Bosnia and Herzegovina | 293609(21859 7to366887) | -57.10% | 17827(13473to 22145) | 0.24(0.16to0.3 1) | 152300(12759 5to176923) | -54.50% | 9454(7921to10 947) | -0.05(-0.2to0.11) | 58(25to114) | -54.53% | 4(2to7) | -0.04(-0.19to0.11) |
| Botswana | 388367(23637 8to536041) | 22.15% | 15712(9656to2 1634) | 0(-0.01to0.01) | 165806(12582 5to216303) | 21.06% | 6767(5119to88 37) | -0.02(-0.03to-0.02) | 64(27to135) | 21.10% | 3(1to6) | -0.01(-0.02to-0.01) |
| Brazil | 28699728(193 33168to379811 10) | -11.52% | 17450(11823to 23030) | -0.15(-0.48to0.18) | 13513288(108 70958to160867 78) | -13.15% | 8343(6679to99 10) | -0.04(-0.34to0.27) | 5189(2230to10 804) | -13.04% | 3(1to7) | -0.03(-0.33to0.27) |
| Brunei  Darussalam | 58385(39516to 75863) | 3.06% | 17736(12007to 23034) | 0.01(0to0.02) | 25763(20468to 31302) | -1.21% | 7920(6296to96 09) | 0.01(0to0.02) | 10(4to21) | -1.26% | 3(1to6) | 0.01(0to0.02) |
| Bulgaria | 613692(43884 8to802198) | -40.25% | 17905(12869to 23376) | 0.1(0.06to0.14) | 290059(23376 4to344850) | -42.35% | 8655(6952to10 275) | 0.08(0.04to0.1 3) | 111(48to224) | -42.32% | 3(1to7) | 0.08(0.04to0.1 3) |
| Burkina Faso | 5994306(3907 222to8087416) | 118.89% | 16315(10582to 22096) | -0.12(-0.2to-  0.03) | 2730148(1971 082to3491938) | 113.49% | 7229(5266to92 39) | 0.55(0.16to0.9 3) | 1039(427to217 7) | 114.41% | 3(1to6) | 0.55(0.17to0.9 4) |
| Burundi | 3019657(1939 054to4121334) | 103.91% | 15784(9953to2 1694) | 0.01(0to0.03) | 1456442(1078 805to1846793) | 103.55% | 7429(5572to93 96) | 0.06(0.05to0.0 6) | 558(234to1149 ) | 104.30% | 3(1to6) | 0.07(0.06to0.0 8) |
| Cabo Verde | 89104(54667to 121132) | -1.98% | 16077(9888to2 1905) | -0.01(-0.02to0) | 37652(27096to 49347) | -7.15% | 6858(4923to89 92) | -0.12(-0.13to-0.11) | 14(6to30) | -6.96% | 3(1to5) | -0.11(-0.12to-0.11) |
| Cambodia | 2858250(1922 068to3754994) | 3.38% | 16194(10867to 21301) | 0.01(-0.12to0.14) | 1443467(1131 989to1760325) | -0.60% | 8156(6393to99 52) | 0(-0.32to0.32) | 550(236to1083 ) | -0.54% | 3(1to6) | 0.01(-0.31to0.32) |
| Cameroon | 6826286(4184 159to9344355) | 148.05% | 16103(9866to2 2057) | 0.01(-0.01to0.03) | 2988539(2141 526to3850804) | 135.43% | 7053(5054to90 93) | -0.05(-0.06to-0.04) | 1145(464to239 9) | 136.00% | 3(1to6) | -0.04(-0.05to-0.03) |
| Canada | 3478692(2208 400to4774671) | 7.59% | 16366(10427to 22274) | -0.01(-0.04to0.02) | 1456987(1115 409to1869933) | 5.80% | 7002(5297to90 43) | 0.04(0.02to0.0 5) | 560(238to1196 ) | 5.75% | 3(1to6) | 0.04(0.03to0.0 6) |
| Central African Republic | 1248396(7663 82to1707060) | 81.45% | 15993(9758to2 1924) | -0.01(-0.02to0.01) | 570147(41811 4to725548) | 79.95% | 7162(5281to90 91) | 0.06(0.06to0.0 7) | 217(90to455) | 80.34% | 3(1to6) | 0.07(0.06to0.0 8) |
| Chad | 4796790(3016 006to6447557) | 186.45% | 16256(10140to 21970) | -0.01(-0.02to0) | 2158392(1542 004to2813551) | 174.95% | 7065(5098to91 68) | -0.07(-0.09to-0.05) | 824(340to1678 ) | 175.24% | 3(1to6) | -0.06(-0.08to-0.04) |
| Chile | 2399658(1662 971to3201114) | -6.61% | 18828(13175to 24912) | -0.04(-0.08to-0.01) | 1045703(8313 32to1258716) | -6.89% | 8404(6658to10 084) | -0.42(-0.63to-0.21) | 402(171to831) | -7.29% | 3(1to7) | -0.43(-0.63to-0.22) |
| China | 137318634(98 321622to17663 1394) | -25.69% | 17539(12458to 22598) | 0.23(0.16to0.2 9) | 67172112(540 28765to796582 70) | -28.74% | 8401(6762to99 81) | 0.07(0.03to0.1 2) | 25868(11288to 54496) | -28.63% | 3(1to7) | 0.08(0.03to0.1 3) |
| Colombia | 5125437(3472 021to7188703) | -25.99% | 13775(9324to1 9232) | -1.24(-1.51to-0.97) | 2231245(1842 284to2696738) | -28.25% | 5995(4945to72 69) | -0.95(-1.19to-0.71) | 859(367to1811 ) | -28.05% | 2(1to5) | -0.94(-1.18to- 0.7) |
| Comoros | 126769(77666t o174461) | 9.37% | 15687(9633to2 1504) | 0.02(0to0.04) | 57977(43907to 73927) | 5.72% | 7240(5459to92 25) | 0.03(0.02to0.0 4) | 22(9to48) | 5.51% | 3(1to6) | 0.04(0.03to0.0 5) |
| Congo | 1116046(6635 08to1534782) | 83.35% | 15828(9436to2 1791) | 0.01(0to0.01) | 476549(34765 3to629223) | 78.74% | 6762(4929to89 35) | 0(-0.02to0.02) | 182(76to385) | 78.93% | 3(1to5) | 0(-0.01to0.02) |
| Cook Islands | 2504(1708to32 76) | -38.18% | 17599(12108to 22949) | 0.02(0.01to0.0 3) | 1133(885to138 0) | -41.79% | 8099(6329to98 54) | -0.05(-0.06to-0.03) | 0(0to1) | -41.81% | 3(1to6) | -0.05(-0.06to-0.03) |
| Costa Rica | 601197(39411 3to813162) | -8.84% | 16465(10863to 22192) | 0.01(-0.01to0.02) | 262198(20105 2to328650) | -12.71% | 7295(5574to91 92) | -0.07(-0.08to-0.06) | 101(44to210) | -12.72% | 3(1to6) | -0.07(-0.08to-0.06) |
| Croatia | 377112(26382 1to492953) | -38.56% | 17898(12582to 23188) | -0.01(-0.02to0) | 176270(14078 8to212680) | -38.26% | 8651(6855to10 384) | 0.01(-0.01to0.02) | 68(29to136) | -38.16% | 3(1to7) | 0.01(-0.01to0.02) |
| Cuba | 1098671(7353 99to1461143) | -26.15% | 17189(11662to 22769) | 0(-0.02to0.01) | 508291(39737 6to617624) | -28.68% | 8180(6368to99 28) | -0.01(-0.02to0) | 195(83to417) | -28.59% | 3(1to7) | -0.01(-0.02to0) |
| Cyprus | 102762(58168t o148868) | 8.80% | 13789(7811to1 9960) | 0(-0.02to0.02) | 39522(28049to 52074) | 8.59% | 5302(3765to69 86) | 0.01(-0.01to0.04) | 15(6to30) | 8.53% | 2(1to4) | 0.01(-0.02to0.04) |
| Czechia | 1057126(7424 97to1356701) | -18.39% | 17981(12716to 23032) | 0.01(-0.01to0.03) | 500677(40632 8to594719) | -16.47% | 8578(6952to10 177) | 0(-0.01to0.01) | 191(82to384) | -16.67% | 3(1to7) | 0(-0.01to0.01) |
| Cote d'Ivoire | 6120176(3794 222to8308423) | 84.88% | 16166(10023to 22067) | 0(-0.01to0.01) | 2699829(1934 853to3506839) | 80.21% | 7002(5050to90 61) | -0.02(-0.04to0) | 1031(441to215 3) | 80.74% | 3(1to6) | -0.01(-0.03to0) |
| Democratic  People's  Republic of  Korea | 2643753(1769 152to3518043) | -24.31% | 15811(10565to 20963) | 0.01(0to0.02) | 1395657(1110 391to1689469) | -27.12% | 8376(6661to10 138) | 0.1(0.09to0.11) | 534(228to1071 ) | -27.12% | 3(1to6) | 0.1(0.09to0.12) |
| Democratic Republic of the Congo | 21398332(131 96927to290683 16) | 109.95% | 16006(9856to2 1780) | 0(-0.01to0.02) | 9546889(7039 426to12243430 ) | 103.11% | 7083(5229to90 91) | 0.08(0.06to0.1 1) | 3636(1511to73 64) | 103.02% | 3(1to5) | 0.1(0.07to0.12) |
| Denmark | 379456(16896 4to624815) | 15.23% | 11608(5119to1 9044) | -2.36(-3.14to-1.57) | 119433(68569t o170018) | 13.67% | 3694(2113to52 60) | -3.52(-4.67to-2.36) | 46(17to96) | 13.31% | 1(1to3) | -3.53(-4.67to-2.37) |
| Djibouti | 233952(15009 8to315657) | 99.15% | 15761(10043to 21291) | 0(-0.02to0.01) | 107770(80746t o137944) | 92.13% | 7113(5332to91 01) | 0.04(0.02to0.0 6) | 41(17to86) | 92.03% | 3(1to6) | 0.05(0.03to0.0 7) |
| Dominica | 8458(5551to11 344) | -42.07% | 17365(11637to 22883) | 0(-0.02to0.01) | 3786(2990to45 80) | -45.03% | 8092(6336to97 61) | -0.06(-0.07to-0.05) | 1(1to3) | -44.87% | 3(1to6) | -0.06(-0.07to-0.05) |
| Dominican  Republic | 1831055(1256 299to2398645) | 11.71% | 17235(11775to 22653) | -0.01(-0.02to0.01) | 867530(67058 7to1059397) | 9.61% | 8080(6256to98 62) | -0.05(-0.06to-0.05) | 333(143to705) | 9.78% | 3(1to7) | -0.05(-0.06to-0.04) |
| Ecuador | 2942047(1966 826to3943170) | 26.34% | 16848(11262to 22560) | 0.34(0.18to0.5) | 1400896(1104 201to1709170) | 25.40% | 8058(6343to98 24) | -0.92(-1.21to-0.62) | 539(227to1122 ) | 25.82% | 3(1to6) | -0.9(-1.19to- 0.61) |
| Egypt | 20193521(140 76682to266621 21) | 50.09% | 17322(12096to 22858) | 0.01(0to0.01) | 9118156(7109 928to11266060 ) | 42.43% | 7926(6164to98 03) | -0.08(-0.09to-0.07) | 3505(1504to73 07) | 42.84% | 3(1to6) | -0.07(-0.08to-0.05) |
| El Salvador | 987362(65189 7to1327121) | -17.66% | 16462(10955to 22093) | 0(-0.02to0.02) | 437562(33791 5to548760) | -21.10% | 7360(5674to92 35) | -0.09(-0.1to- 0.09) | 168(71to359) | -20.65% | 3(1to6) | -0.08(-0.09to-0.07) |
| Equatorial  Guinea | 307841(17734 0to427505) | 164.65% | 15791(9107to2 1845) | -0.09(-0.11to-0.06) | 124510(92413t o166028) | 132.61% | 6476(4757to86 46) | -0.37(-0.42to-0.32) | 48(20to101) | 134.38% | 2(1to5) | -0.34(-0.38to-0.29) |
| Eritrea | 1456069(9047 73to1976970) | 85.47% | 15734(9778to2 1378) | 0(-0.02to0.01) | 672999(50560 3to857235) | 79.02% | 7256(5452to92 42) | -0.04(-0.07to-0.02) | 258(108to538) | 80.21% | 3(1to6) | -0.03(-0.05to0) |
| Estonia | 135314(94648t o176911) | -37.90% | 17929(12624to 23389) | 0.02(0to0.04) | 63212(50624to 75300) | -40.75% | 8530(6810to10 122) | -0.05(-0.06to-0.03) | 24(11to50) | -40.83% | 3(1to7) | -0.04(-0.06to-0.03) |
| Eswatini | 230621(14789 6to311578) | 4.48% | 16120(10369to 21704) | -0.02(-0.03to-0.01) | 111927(86805t o137950) | 2.07% | 7851(6079to96 78) | 0(-0.01to0) | 43(19to87) | 1.89% | 3(1to6) | 0(-0.01to0) |
| Ethiopia | 27356143(182 67460to36579518) | 83.94% | 16966(11255to 22764) | -0.03(-0.04to-0.03) | 12340648(933 3618to15458052) | 80.22% | 7574(5746to94 79) | -0.02(-0.02to-0.01) | 4718(1991to97 95) | 80.83% | 3(1to6) | 0(-0.01to0) |
| Fiji | 162667(11416 0to211395) | -5.90% | 17295(12170to 22467) | 0.34(-0.34to1.02) | 77916(62147to 93898) | -6.44% | 8377(6656to10 107) | -0.33(-0.95to0.29) | 30(13to59) | -6.85% | 3(1to6) | -0.33(-0.94to0.29) |
| Finland | 482746(29616 8to671619) | 5.97% | 15747(9816to2 1743) | 0.27(0.15to0.4) | 183039(13798 2to235122) | 13.89% | 6126(4661to78 75) | 0.35(0.15to0.5 6) | 70(30to144) | 13.65% | 2(1to5) | 0.35(0.14to0.5 6) |
| France | 5106244(2857 378to7594291) | 198.35% | 12463(6970to1 8345) | 2.03(1.29to2.7 8) | 2117688(1471 227to2812433) | -20.58% | 5247(3652to70 01) | 0.56(0.1to1.02) | 815(323to1652 ) | -20.31% | 2(1to4) | 0.58(0.12to1.0 4) |
| Gabon | 320592(19233 1to444345) | 40.37% | 15803(9465to2 1869) | 0.01(0to0.02) | 133571(99302t o175768) | 34.08% | 6588(4895to86 68) | 0.03(0.02to0.0 5) | 51(21to107) | 34.02% | 3(1to5) | 0.03(0.02to0.0 5) |
| Gambia | 521193(32100 3to717144) | 93.90% | 16141(9954to2 2223) | -0.01(-0.01to0) | 233064(17294 0to298364) | 87.70% | 7176(5330to91 85) | -0.04(-0.06to-0.03) | 89(37to185) | 88.04% | 3(1to6) | -0.03(-0.05to-0.02) |
| Georgia | 471070(32829 1to617195) | -45.54% | 17837(12476to 23402) | -0.36(-0.46to-0.25) | 223877(17857 4to266694) | -44.91% | 8555(6830to10 173) | 0.64(0.45to0.8 3) | 85(37to168) | -44.95% | 3(1to6) | 0.63(0.45to0.8 2) |
| Germany | 5722429(3236 792to8176719) | 26.39% | 14184(8056to2 0242) | 0.45(0.21to0.6 9) | 2116054(1547 781to2699348) | -31.01% | 5264(3840to67 15) | -1.92(-2.36to-1.47) | 814(331to1625 ) | -31.10% | 2(1to4) | -1.92(-2.36to-1.47) |
| Ghana | 6331551(3949 726to8804767) | 61.93% | 16025(9999to2 2290) | -0.02(-0.03to-0.01) | 2769046(2006 883to3586524) | 55.84% | 6995(5080to90 58) | -0.07(-0.09to-0.05) | 1059(437to222 9) | 56.35% | 3(1to6) | -0.07(-0.08to-0.05) |
| Greece | 675972(41434 2to960176) | -0.22% | 13621(8480to1 9184) | -0.06(-0.61to0.49) | 299825(22051 7to386726) | -42.94% | 6154(4520to79 37) | -3.12(-3.92to-2.31) | 116(49to248) | -42.89% | 2(1to5) | -3.11(-3.91to-2.31) |
| Greenland | 6709(4491to88 49) | -19.43% | 16513(11053to 21744) | 0.07(0.04to0.1) | 3154(2467to39 52) | -22.07% | 7775(6074to97 43) | 0.09(0.02to0.1 6) | 1(1to3) | -21.96% | 3(1to6) | 0.09(0.02to0.1 5) |
| Grenada | 13134(8726to1 7640) | -33.82% | 17266(11665to 23051) | 0.02(0to0.03) | 6003(4676to73 20) | -36.08% | 8074(6254to98 35) | -0.06(-0.08to-0.05) | 2(1to5) | -36.03% | 3(1to7) | -0.06(-0.08to-0.05) |
| Guam | 27825(19336to 36451) | 10.69% | 17496(12148to 22917) | 0.01(0to0.03) | 12952(10226to 15720) | 7.08% | 8131(6419to98 66) | 0(0to0.01) | 5(2to10) | 6.97% | 3(1to7) | 0(0to0.01) |
| Guatemala | 3356003(2183 699to4516463) | 57.29% | 16543(10777to 22232) | -0.02(-0.03to0) | 1482510(1136 634to1853778) | 49.23% | 7332(5619to91 65) | -0.04(-0.06to-0.02) | 569(245to1171 ) | 49.74% | 3(1to6) | -0.03(-0.05to0) |
| Guinea | 3295912(2080 983to4459015) | 104.49% | 16201(10202to 22002) | -0.01(-0.03to0.01) | 1485221(1085 104to1916783) | 95.00% | 7180(5266to92 56) | -0.02(-0.03to-0.01) | 566(240to1189 ) | 94.99% | 3(1to6) | -0.02(-0.03to-0.01) |
| Guinea-Bissau | 451935(28776 4to608249) | 67.50% | 16051(10247to 21718) | 0(-0.02to0.01) | 204424(14871 7to263974) | 61.50% | 7203(5266to92 87) | -0.03(-0.04to-0.02) | 78(33to161) | 61.80% | 3(1to6) | -0.02(-0.03to-0.01) |
| Guyana | 124843(85808t o164807) | -25.56% | 17246(11880to 22716) | 0(-0.01to0.01) | 58948(45811to 71113) | -27.33% | 8171(6354to98 61) | -0.06(-0.07to-0.05) | 23(10to47) | -27.23% | 3(1to6) | -0.06(-0.07to-0.05) |
| Haiti | 2557850(1785 595to3376102) | 55.40% | 17132(11913to 22627) | -0.04(-0.05to-0.02) | 1247015(9780 50to1491053) | 53.76% | 8301(6520to99 33) | 0(-0.01to0.01) | 476(210to970) | 53.56% | 3(1to6) | -0.01(-0.02to0) |
| Honduras | 1890647(1253 685to2514791) | 45.36% | 16517(10964to 21942) | 0(0to0.01) | 853796(65462 7to1075012) | 41.65% | 7483(5735to94 28) | -0.05(-0.06to-0.04) | 329(139to683) | 42.46% | 3(1to6) | -0.04(-0.05to-0.02) |
| Hungary | 849180(60377 5to1096653) | -19.99% | 17978(12877to 23095) | 0.34(0.16to0.5 2) | 398299(32323 0to471098) | -40.49% | 8585(6958to10 145) | -0.57(-0.83to-0.31) | 152(66to301) | -40.48% | 3(1to6) | -0.56(-0.82to- 0.3) |
| Iceland | 32474(18705to 47566) | 8.15% | 13792(7880to2 0193) | 0.03(0.02to0.0 4) | 12191(8632to1 6092) | 5.28% | 5245(3691to69 24) | 0(-0.03to0.02) | 5(2to10) | 5.15% | 2(1to4) | 0(-0.03to0.03) |
| India | 221407837(15 0871274to2922 46998) | 7.55% | 17138(11901to 22433) | -0.08(-0.1to-0.06) | 98199025(774 90240to119506 981) | 4.73% | 7805(6106to94 98) | -0.06(-0.11to- 0.01) | 37573(16289to 80500) | 5.09% | 3(1to6) | -0.05(-0.1to0) |
| Indonesia | 39277787(275 96309to51187782) | -6.53% | 17535(12525to 22704) | -0.01(-0.02to0) | 18343127(148 38517to218348 89) | -9.94% | 8475(6819to10 075) | -0.05(-0.12to0.02) | 7049(3090to14 790) | -9.61% | 3(1to7) | -0.04(-0.11to0.03) |
| Iran (Islamic Republic of) | 13114476(934 0746to17128572) | -23.04% | 18066(12866to 23617) | 0.02(0to0.03) | 6073198(4809 736to7247728) | -21.86% | 8373(6628to99 89) | 0.08(0.04to0.1 3) | 2336(1016to48 81) | -21.69% | 3(1to7) | 0.09(0.05to0.1 3) |
| Iraq | 8312394(5742 819to10912594 | 67.39% | 17230(11921to 22575) | -0.02(-0.04to-0.01) | 3778805(2934 320to4643234) | 64.70% | 7888(6107to97 06) | -0.01(-0.04to0.02) | 1451(624to299 6) | 65.11% | 3(1to6) | -0.01(-0.04to0.02) |
| Ireland | 469304(24551 3to708239) | 5.72% | 13011(6795to1 9636) | 0.04(0.02to0.0 5) | 162652(11060 3to220530) | 5.24% | 4568(3095to61 93) | 0.03(0to0.05) | 63(26to126) | 5.14% | 2(1to3) | 0.03(0to0.06) |
| Israel | 1323276(9027 96to1753924) | 96.12% | 14445(9768to1 9190) | 0.5(0.43to0.58) | 643101(52050 9to778977) | 86.04% | 6985(5690to84 36) | 0.76(0.58to0.9 4) | 247(107to515) | 85.82% | 3(1to6) | 0.76(0.58to0.9 3) |
| Italy | 4363453(2698 727to6058773) | -10.06% | 15863(9841to2 1943) | -0.06(-0.31to0.19) | 1733808(1306 307to2256787) | -10.42% | 6514(4843to85 62) | -0.55(-0.83to-0.27) | 667(281to1419 ) | -10.39% | 3(1to5) | -0.55(-0.83to-0.27) |
| Jamaica | 362667(23963 0to486516) | -28.14% | 17224(11627to 22865) | -0.02(-0.03to-0.01) | 165633(13006 9to199228) | -29.63% | 8153(6368to97 79) | -0.03(-0.04to-0.01) | 64(27to135) | -29.51% | 3(1to7) | -0.02(-0.04to-0.01) |
| Japan | 9370114(6277 956to12552306 | -31.64% | 17370(11849to 23019) | -0.17(-0.21to-0.13) | 3738066(2925 293to4772978) | -37.13% | 7112(5527to91 31) | -0.88(-1.01to-0.74) | 1437(614to298 3) | -37.11% | 3(1to6) | -0.87(-1.01to-0.73) |
| Jordan | 2195134(1483 159to2955454) | 123.90% | 16959(11546to 22632) | 0.95(0.49to1.4) | 979788(75956 6to1227660) | 115.73% | 7703(5951to96 80) | 0.42(0.19to0.6 6) | 376(160to783) | 115.87% | 3(1to6) | 0.43(0.19to0.6 6) |
| Kazakhstan | 3336200(2371 201to4291302) | 1.55% | 18134(12884to 23421) | 0.03(0.01to0.0 4) | 1558224(1239 588to1848755) | 0.17% | 8388(6692to99 44) | -0.02(-0.03to-0.01) | 596(259to1206 ) | 0.43% | 3(1to6) | -0.01(-0.02to0) |
| Kenya | 10842161(718 2668to1442590 8) | 66.97% | 16274(10804to 21618) | -0.06(-0.09to-0.03) | 5325572(4276 758to6410833) | 66.36% | 8075(6465to97 33) | 0.12(0.07to0.1 6) | 2043(901to423 7) | 66.73% | 3(1to6) | 0.13(0.08to0.1 7) |
| Kiribati | 25420(17946to 33387) | 41.23% | 17127(12088to 22503) | 0(-0.02to0.01) | 12679(10022to 15210) | 38.28% | 8550(6751to10 261) | -0.01(-0.01to0) | 5(2to10) | 38.41% | 3(1to7) | 0(-0.01to0.01) |
| Kuwait | 521287(35678 6to687665) | 100.32% | 17111(11712to 22562) | 1.32(1.13to1.5 1) | 237113(17907 9to298180) | 48.97% | 7798(5890to98 09) | 0.07(-0.07to0.22) | 91(39to185) | 49.33% | 3(1to6) | 0.07(-0.07to0.22) |
| Kyrgyzstan | 1360600(9633 21to1739426) | 27.37% | 17876(12652to 22966) | -0.01(-0.02to0.01) | 656114(52651 6to776130) | 26.41% | 8569(6877to10 153) | 0.02(0to0.03) | 250(107to501) | 26.28% | 3(1to7) | 0.01(0to0.03) |
| Lao People's Democratic Republic | 1266008(8434 39to1665691) | 20.93% | 16206(10751to 21313) | 0.05(0.01to0.0 9) | 642099(50430 9to790670) | 17.96% | 8159(6420to10 053) | 0.12(-0.13to0.36) | 245(105to504) | 17.83% | 3(1to6) | 0.12(-0.13to0.37) |
| Latvia | 189492(13441 3to246986) | -50.51% | 17958(12722to 23413) | -0.19(-0.25to-0.12) | 89864(70666to 107394) | -47.84% | 8501(6679to10 150) | 0.01(-0.02to0.03) | 35(15to72) | -47.73% | 3(1to7) | 0(-0.02to0.03) |
| Lebanon | 854459(58978 6to1124827) | 14.03% | 17304(11931to 22834) | 0.01(-0.01to0.04) | 391297(30579 8to488347) | 10.12% | 7847(6144to97 80) | -0.05(-0.06to-0.03) | 150(65to318) | 10.26% | 3(1to6) | -0.04(-0.06to-0.03) |
| Lesotho | 358960(22791 8to495979) | -14.45% | 15818(10031to 21760) | -0.01(-0.02to0.01) | 158085(12150 2to201852) | -16.62% | 7044(5404to89 92) | -0.03(-0.04to-0.02) | 61(26to124) | -16.66% | 3(1to6) | -0.03(-0.04to-0.02) |
| Liberia | 1055554(6780 54to1417315) | 101.75% | 16199(10423to 21779) | 0(-0.01to0) | 470142(34962 9to598174) | 99.69% | 7268(5379to92 47) | -0.03(-0.06to-0.01) | 180(76to370) | 100.35% | 3(1to6) | -0.02(-0.04to0.01) |
| Libya | 857167(58473 4to1140006) | -24.38% | 17315(12147to 22664) | 0.03(0.02to0.0 5) | 378084(29773 8to467931) | -27.69% | 7942(6177to98 48) | 0.02(-0.01to0.05) | 145(63to306) | -27.57% | 3(1to6) | 0.02(-0.01to0.05) |
| Lithuania | 261877(18529 5to334230) | -49.32% | 17756(12569to 22662) | -0.32(-0.9to0.27) | 126703(10064 6to150328) | -50.09% | 8602(6834to10 203) | 0(-0.39to0.39) | 49(21to101) | -49.97% | 3(1to7) | 0(-0.39to0.38) |
| Luxembourg | 47564(26759to 68846) | 51.48% | 13799(7732to1 9944) | 0.04(0.02to0.0 7) | 18035(12832to 23799) | 50.35% | 5273(3738to69 66) | 0.02(0to0.03) | 7(3to14) | 50.20% | 2(1to4) | 0.02(0to0.03) |
| Madagascar | 6154052(4006 491to8175215) | 125.83% | 15987(10365to 21306) | 0.39(0.28to0.4 9) | 3104605(2409 949to3775344) | 42.83% | 8002(6217to97 19) | -1.2(-1.51to- 0.88) | 1189(508to244 1) | 43.53% | 3(1to6) | -1.18(-1.49to-0.86) |
| Malawi | 4327477(2710 718to5915244) | 76.05% | 15742(9857to2 1449) | 0(-0.02to0.01) | 1982230(1515 124to2487548) | 67.81% | 7323(5560to91 93) | -0.02(-0.03to-0.01) | 759(320to1573 ) | 68.36% | 3(1to6) | -0.01(-0.02to0) |
| Malaysia | 4372348(2889 990to5781920) | 15.64% | 16440(10907to 21658) | -0.02(-0.03to0) | 2085882(1601 850to2555137) | 10.36% | 7938(6104to97 14) | -0.05(-0.07to-0.04) | 802(337to1661 ) | 10.63% | 3(1to6) | -0.05(-0.06to-0.04) |
| Maldives | 63848(43099to 85085) | 3.66% | 16246(10834to 21688) | -0.01(-0.03to0.01) | 31491(24724to 38607) | 1.80% | 7866(6201to96 54) | -0.04(-0.05to-0.03) | 12(5to25) | 2.25% | 3(1to6) | -0.02(-0.03to0) |
| Mali | 5873268(3762 213to7991749) | 149.28% | 16164(10296to 22132) | -0.02(-0.03to0) | 2689013(1938 101to3472529) | 141.50% | 7178(5191to92 10) | -0.08(-0.09to-0.06) | 1023(439to211 7) | 141.59% | 3(1to6) | -0.07(-0.09to-0.06) |
| Malta | 30269(17319to 44876) | -26.09% | 13747(7851to2 0359) | 0.07(0.06to0.0 8) | 11591(8185to1 5272) | -27.20% | 5266(3717to69 36) | 0(-0.03to0.02) | 4(2to9) | -27.19% | 2(1to4) | -0.01(-0.03to0.02) |
| Marshall  Islands | 11193(7738to1 4606) | -20.39% | 17377(12042to 22652) | -0.01(-0.02to0.01) | 5344(4281to64 40) | -20.71% | 8405(6705to10 125) | 0.01(0to0.02) | 2(1to4) | -20.83% | 3(1to6) | 0.01(0to0.02) |
| Mauritania | 913770(55875 8to1248796) | 70.14% | 16077(9836to2 1883) | 0(-0.01to0.02) | 394100(28320 0to506540) | 61.29% | 7042(5043to90 75) | -0.06(-0.07to-0.04) | 151(62to307) | 61.09% | 3(1to5) | -0.05(-0.07to-0.04) |
| Mauritius | 117032(76586t o157674) | -35.35% | 16306(10766to 21762) | -0.02(-0.04to0) | 54394(42126to 67205) | -37.49% | 7842(6064to96 75) | -0.04(-0.05to-0.02) | 21(9to43) | -37.35% | 3(1to6) | -0.03(-0.05to-0.02) |
| Mexico | 19573553(132 47768to26072073) | -3.90% | 17551(11984to 23325) | 0.22(0.14to0.2 9) | 8679102(6781 798to10541112 ) | -6.45% | 7914(6161to96 20) | 0.48(0.3to0.65) | 3337(1425to69 83) | -6.39% | 3(1to6) | 0.48(0.31to0.6 5) |
| Micronesia  (Federated  States of) | 18614(13043to 24354) | -34.66% | 17362(12332to 22589) | 0(-0.02to0.02) | 8836(6943to10 755) | -37.65% | 8459(6627to10 261) | 0(-0.01to0.01) | 3(1to7) | -37.67% | 3(1to6) | 0(-0.01to0.01) |
| Monaco | 2349(1287to34 34) | 43.75% | 13561(7392to1 9797) | 0.04(0.03to0.0 5) | 865(607to1161 ) | 42.72% | 5032(3534to67 50) | 0.03(0.01to0.0 4) | 0(0to1) | 42.96% | 2(1to4) | 0.03(0.01to0.0 5) |
| Mongolia | 669062(47564 9to855729) | 19.39% | 18029(12542to 23145) | 0.04(0.03to0.0 5) | 321128(25338 5to382008) | 16.07% | 8433(6717to10 061) | -0.06(-0.07to-0.05) | 123(53to249) | 16.68% | 3(1to7) | -0.05(-0.06to-0.04) |
| Montenegro | 66476(47447to 86822) | -33.85% | 17769(12717to 23002) | 0(-0.01to0.02) | 31985(25664to 38268) | -33.85% | 8728(7010to10 416) | 0.01(0to0.01) | 12(5to25) | -33.66% | 3(1to7) | 0.01(0to0.02) |
| Morocco | 5663173(3949 209to7437961) | -3.97% | 17302(12103to 22510) | -0.02(-0.03to-0.01) | 2570544(1980 044to3150470) | -7.35% | 7982(6151to97 84) | -0.01(-0.03to0) | 987(436to2127 ) | -7.21% | 3(1to7) | -0.01(-0.02to0) |
| Mozambique | 7759931(4930 632to10339071 | 133.56% | 15722(9955to2 1104) | 0.01(0to0.02) | 3645059(2745 544to4674035) | 124.67% | 7294(5513to93 55) | -0.11(-0.12to-0.1) | 1395(589to286 7) | 126.07% | 3(1to6) | -0.09(-0.09to-0.08) |
| Myanmar | 8185356(4950 060to11332962 | -6.47% | 15824(9563to2 1921) | -0.29(-0.59to0.01) | 3348002(2564 946to4442050) | -10.64% | 6522(4983to86 48) | -0.56(-1.06to-0.05) | 1274(529to266 2) | -10.53% | 2(1to5) | -0.55(-1.06to-0.05) |
| Namibia | 463344(28600 3to633114) | 41.37% | 15744(9686to2 1531) | 0(-0.01to0.02) | 200584(15084 2to261096) | 37.79% | 6790(5114to88 28) | -0.04(-0.05to-0.03) | 77(32to162) | 38.07% | 3(1to5) | -0.03(-0.04to-0.02) |
| Nauru | 2396(1658to31 11) | -12.81% | 17530(12126to 22752) | 0(-0.01to0.01) | 1145(901to139 5) | -15.39% | 8314(6553to10 134) | 0.05(0.02to0.0 7) | 0(0to1) | -15.49% | 3(1to7) | 0.05(0.02to0.0 7) |
| Nepal | 5530489(4078 495to7071881) | 7.23% | 17551(13031to 22300) | 0(-0.02to0.03) | 2856909(2451 219to3237734) | 13.69% | 9194(7907to10 429) | 0.33(0.27to0.3 8) | 1096(475to217 1) | 13.93% | 4(2to7) | 0.34(0.29to0.3 9) |
| Netherlands | 1373265(8752 26to1878202) | 8.14% | 14643(9376to1 9955) | 0.27(0.22to0.3 1) | 529199(42456 0to636050) | 6.61% | 5699(4519to68 68) | 0.31(0.27to0.3 5) | 204(86to404) | 6.49% | 2(1to4) | 0.3(0.26to0.33) |
| New Zealand | 367991(18662 1to556064) | -10.68% | 11859(6055to1 7854) | -1.23(-1.44to-1.01) | 105402(63938t o152775) | -38.78% | 3434(2074to49 63) | -3.01(-3.44to-2.57) | 41(16to93) | -38.68% | 1(1to3) | -2.99(-3.42to-2.56) |
| Nicaragua | 1166150(7597 37to1568595) | 7.92% | 16645(10902to 22308) | 0.01(0to0.02) | 515930(39352 9to652384) | 4.11% | 7458(5685to94 44) | -0.06(-0.07to-0.04) | 199(85to419) | 4.19% | 3(1to6) | -0.05(-0.06to-0.03) |
| Niger | 6936723(4973 054to9040455) | 196.54% | 16240(11461to 21337) | -0.01(-0.04to0.01) | 3725275(2915 321to4485601) | 194.55% | 8400(6625to10 173) | -0.03(-0.05to-0.01) | 1419(633to292 9) | 194.81% | 3(1to7) | -0.02(-0.04to-0.01) |
| Nigeria | 53784642(334 08036to737343 47) | 139.37% | 16380(10176to 22483) | -0.06(-0.09to-0.02) | 21082438(156 47599to283940 76) | 125.79% | 6393(4750to85 88) | -0.21(-0.3to- 0.12) | 8032(3373to17 309) | 126.55% | 2(1to5) | -0.2(-0.29to- 0.1) |
| Niue | 238(166to310) | -54.88% | 17354(12206to 22500) | -0.01(-0.02to0.01) | 112(89to136) | -54.81% | 8357(6598to10 111) | 0.01(-0.01to0.02) | 0(0to0) | -54.82% | 3(1to7) | 0.01(-0.01to0.02) |
| North  Macedonia | 213803(15156 0to274927) | -35.65% | 17775(12593to 22735) | 0.02(0.02to0.0 3) | 103692(83843t o122931) | -35.48% | 8728(7072to10 332) | -0.01(-0.01to0) | 40(17to82) | -35.35% | 3(1to7) | -0.01(-0.02to0) |
| Northern  Mariana | 4156(2886to54 51) | -43.00% | 17450(12165to 22602) | -0.01(-0.02to-0.01) | 1917(1527to2311) | -45.63% | 8218(6532to98 99) | 0.03(0.02to0.0 4) | 1(0to2) | -45.57% | 3(1to7) | 0.03(0.02to0.0 5) |
| Norway | 543152(34385 2to741006) | 25.09% | 16594(10530to 22515) | 0.1(0.02to0.19) | 208291(15872 3to273444) | 24.32% | 6508(4925to86 12) | 0.22(-0.12to0.57) | 80(34to173) | 24.30% | 3(1to5) | 0.23(-0.11to0.57) |
| Oman | 648518(45553 7to848772) | 35.48% | 17307(12061to 22936) | 0.31(0.23to0.4) | 310952(24190 5to379407) | 7.06% | 8136(6378to99 10) | -0.63(-0.8to- 0.46) | 119(51to249) | 7.68% | 3(1to6) | -0.61(-0.77to-0.44) |
| Pakistan | 56162181(393 47419to72684303) | 71.04% | 18452(12924to 23876) | -0.09(-0.15to-0.03) | 24968276(196 57273to29973024) | 64.35% | 8193(6450to98 32) | -0.5(-0.79to- 0.21) | 9555(4142to19 571) | 64.62% | 3(1to6) | -0.49(-0.79to- 0.2) |
| Palau | 2008(1389to26 42) | -28.27% | 17375(12186to 22631) | -0.01(-0.03to0) | 928(731to1131 ) | -31.17% | 8357(6551to10 119) | 0.02(0to0.04) | 0(0to1) | -31.00% | 3(1to7) | 0.02(0.01to0.0 4) |
| Palestine | 1134005(7959 28to1460297) | 87.73% | 17266(12153to 22145) | -0.02(-0.05to0.02) | 504933(39657 6to622099) | 87.86% | 7746(6059to95 82) | 0.1(-0.07to0.27) | 194(80to407) | 88.15% | 3(1to6) | 0.1(-0.07to0.28) |
| Panama | 663944(43991 5to897951) | 38.55% | 16505(10980to 22308) | 0.03(0.02to0.0 4) | 289869(22248 4to367700) | 33.39% | 7253(5550to92 10) | -0.08(-0.09to- 0.08) | 111(47to228) | 33.47% | 3(1to6) | -0.08(-0.09to-0.07) |
| Papua New  Guinea | 2237976(1610 636to2872319) | 119.90% | 17335(12317to 22420) | 0.01(0to0.02) | 1131683(8906 90to1362698) | 123.83% | 8484(6677to10 196) | -0.02(-0.03to0) | 431(181to864) | 123.59% | 3(1to7) | -0.02(-0.03to0) |
| Paraguay | 1101395(6997 96to1482712) | 12.78% | 16119(10389to 21706) | 0(-0.01to0.01) | 516628(39613 7to639413) | 9.36% | 7683(5876to95 13) | -0.02(-0.03to- 0.01) | 198(84to423) | 9.39% | 3(1to6) | -0.02(-0.03to-0.01) |
| Peru | 5382186(3495 684to7169729) | 8.04% | 16862(10996to 22459) | -0.01(-0.02to0) | 2542102(1992 873to3076240) | 5.28% | 8008(6259to96 83) | -0.06(-0.07to- 0.05) | 976(416to2056 ) | 5.76% | 3(1to6) | -0.05(-0.06to-0.04) |
| Philippines | 21704462(147 39654to28364093) | 39.95% | 17470(11816to 22898) | 0.18(0.12to0.2 5) | 10065430(792 0232to1205192 6) | 37.74% | 8043(6320to96 42) | -0.13(-0.21to- 0.06) | 3865(1693to8057) | 38.04% | 3(1to6) | -0.12(-0.2to- 0.05) |
| Poland | 3746713(2609 592to4918624) | -39.36% | 18187(12815to 23723) | -0.02(-0.03to-0.01) | 1674487(1336 193to1986828) | -38.77% | 8283(6588to98 08) | 0(-0.03to0.02) | 644(282to1346 ) | -38.72% | 3(1to7) | 0(-0.03to0.03) |
| Portugal | 641903(36923 1to945357) | -32.54% | 13700(7781to2 0015) | -0.05(-0.09to-0.01) | 245321(17272 1to324393) | -32.32% | 5348(3710to70 63) | -0.2(-0.27to- 0.13) | 95(39to199) | -32.14% | 2(1to4) | -0.2(-0.28to- 0.12) |
| Puerto Rico | 303915(19702 4to411415) | -48.17% | 17392(11793to 22984) | 0.02(0.01to0.0 3) | 128512(10036 2to156598) | -52.18% | 7969(6149to97 04) | -0.06(-0.07to- 0.04) | 49(21to104) | -52.18% | 3(1to6) | -0.06(-0.07to-0.05) |
| Qatar | 263787(18603 7to342063) | 244.09% | 17433(12409to 22665) | -0.06(-0.1to-0.03) | 124276(98287t o150355) | 227.34% | 8277(6546to10 028) | 0.77(0.53to1.0 1) | 48(20to101) | 228.24% | 3(1to7) | 0.78(0.54to1.0 2) |
| Republic of  Korea | 4288457(2945 875to5488216) | -38.63% | 17775(12318to 22719) | 0.16(0.02to0.3 1) | 1947804(1543 152to2372290) | -39.16% | 8280(6525to10 083) | -0.55(-0.92to- 0.17) | 749(330to1562 ) | -39.26% | 3(1to7) | -0.54(-0.92to-0.17) |
| Republic of  Moldova | 357447(24526 3to457201) | -53.99% | 17798(12458to 22549) | 0.03(0.02to0.0 4) | 168922(13573 6to200528) | -55.75% | 8723(7028to10 342) | -0.01(-0.02to0.01) | 65(28to134) | -55.73% | 3(1to7) | 0(-0.02to0.01) |
| Romania | 1831149(1302 142to2376517) | -31.54% | 17843(12662to 22858) | 0.92(0.67to1.1 7) | 869073(71317 0to1034492) | -56.72% | 8663(7094to10 308) | -0.92(-1.16to- 0.67) | 334(145to692) | -56.67% | 3(1to7) | -0.91(-1.15to-0.67) |
| Russian  Federation | 17811322(126 14828to23078264) | -21.29% | 18261(12964to 23624) | 0(-0.01to0.01) | 8145100(6569 202to9621156) | -24.30% | 8433(6812to9945) | -0.09(-0.12to- 0.05) | 3133(1367to64 56) | -24.28% | 3(1to7) | -0.09(-0.12to-0.05) |
| Rwanda | 2640812(1666 575to3596692) | 38.82% | 15643(9906to2 1262) | -0.01(-0.03to0.01) | 1206633(8967 22to1520698) | 33.75% | 7189(5337to9068) | -0.05(-0.07to- 0.02) | 463(193to957) | 34.37% | 3(1to6) | -0.02(-0.04to0) |
| Saint Kitts and Nevis | 6973(4682to9344) | -18.39% | 17302(11802to 23118) | 0.01(0to0.02) | 3119(2442to37 65) | -20.63% | 7997(6211to9704) | -0.05(-0.06to- 0.04) | 1(1to3) | -20.60% | 3(1to6) | -0.05(-0.06to-0.04) |
| Saint Lucia | 18387(12096to 24625) | -42.11% | 17196(11669to 22681) | -0.02(-0.03to- 0.01) | 8324(6555to10 114) | -44.16% | 8105(6312to9831) | 0(-0.01to0.01) | 3(1to7) | -44.02% | 3(1to6) | 0(0to0.01) |
| Saint Vincent and the Grenadines | 14703(10269to 19503) | -28.96% | 17172(12134to 22621) | 0.74(0.54to0.9 4) | 7231(5872to85 45) | -56.76% | 8673(7059to10 236) | -1.14(-1.44to- 0.83) | 3(1to6) | -56.67% | 3(1to7) | -1.13(-1.43to-0.83) |
| Samoa | 44992(30585to 59525) | 10.59% | 17318(11938to 22744) | 0.02(0to0.04) | 20832(16583to 25153) | 9.27% | 8419(6693to10 141) | -0.01(-0.03to0) | 8(3to16) | 9.03% | 3(1to6) | -0.02(-0.04to-0.01) |
| San Marino | 2509(1452to36 68) | 19.49% | 13779(7922to1 9997) | 0.04(0.02to0.0 6) | 936(656to1250 ) | 18.40% | 5227(3660to69 84) | 0.02(0.01to0.0 4) | 0(0to1) | 18.30% | 2(1to4) | 0.03(0.01to0.0 4) |
| Sao Tome and Principe | 41578(25496to 57690) | 27.88% | 16076(9858to2 2118) | -0.01(-0.03to0) | 17735(12856to 22857) | 22.29% | 7038(5097to90 99) | -0.05(-0.07to-0.04) | 7(3to14) | 22.60% | 3(1to6) | -0.04(-0.06to-0.03) |
| Saudi Arabia | 3531399(2509 036to4564571) | 3.18% | 14441(10377to 18552) | 0.48(0.32to0.6 4) | 2311096(2024 629to2652338) | 41.87% | 9525(8323to10 939) | 0.58(0.35to0.8) | 888(391to1786 ) | 41.99% | 4(2to7) | 0.58(0.35to0.8 1) |
| Senegal | 3993803(2733 475to5139858) | 91.02% | 18691(12783to 24061) | 0.45(0.36to0.5 3) | 1495063(1056 938to1932993) | 53.94% | 6975(4934to90 16) | 0.92(0.58to1.2 5) | 571(234to1143 ) | 54.27% | 3(1to5) | 0.92(0.59to1.2 5) |
| Serbia | 898193(63664 7to1182837) | -29.88% | 17657(12573to 23141) | -0.09(-0.18to0) | 437530(35584 6to520020) | -29.33% | 8838(7194to10 453) | 0.24(-0.09to0.57) | 167(72to339) | -29.24% | 3(1to7) | 0.24(-0.09to0.57) |
| Seychelles | 12481(8094to1 6507) | -7.93% | 16359(10632to 21642) | 0.03(0.01to0.0 4) | 5939(4655to73 15) | -7.69% | 7813(6124to96 32) | -0.02(-0.03to-0.01) | 2(1to5) | -7.31% | 3(1to6) | -0.01(-0.02to0) |
| Sierra Leone | 1872596(1158 471to2583049) | 96.49% | 16011(9771to2 2163) | -0.15(-0.18to-0.13) | 797878(56794 7to1054436) | 62.10% | 6734(4819to88 68) | -0.9(-1.12to- 0.68) | 304(129to635) | 62.47% | 3(1to5) | -0.89(-1.11to-0.67) |
| Singapore | 507242(33876 9to672292) | 30.30% | 17743(11780to 23520) | -0.14(-0.24to-0.05) | 216986(17183 4to271079) | 34.91% | 7498(5945to93 77) | -1.02(-1.28to-0.75) | 84(36to174) | 35.13% | 3(1to6) | -1.01(-1.28to-0.75) |
| Slovakia | 530162(37629 6to686186) | -34.68% | 17888(12735to 23061) | 0.04(0.02to0.0 5) | 252890(20176 3to299434) | -33.99% | 8603(6867to10 182) | -0.05(-0.06to-0.04) | 97(42to199) | -34.02% | 3(1to7) | -0.05(-0.06to-0.04) |
| Slovenia | 199074(14010 1to258478) | -20.10% | 17893(12676to 23163) | 0.04(0.02to0.0 6) | 93158(73781to 111099) | -26.10% | 8551(6774to10 182) | -0.22(-0.29to-0.16) | 36(15to72) | -25.86% | 3(1to7) | -0.22(-0.29to-0.15) |
| Solomon  Islands | 155967(11113 7to202704) | 64.77% | 17229(12244to 22490) | -0.02(-0.03to-0.01) | 78577(61957to 94169) | 64.97% | 8535(6752to10 231) | 0.02(0.01to0.0 4) | 30(13to63) | 65.29% | 3(1to7) | 0.02(0.01to0.0 4) |
| Somalia | 5278930(3478 426to7063827) | 176.93% | 15768(10192to 21250) | 0(-0.01to0.01) | 2645296(2003 793to3312512) | 185.85% | 7668(5827to96 20) | 0.03(0.02to0.0 4) | 1011(433to212 4) | 186.38% | 3(1to6) | 0.04(0.03to0.0 5) |
| South Africa | 8967806(5923 309to12077020 | 20.68% | 16945(11217to 22782) | 0.19(0.14to0.2 5) | 3876137(2986 713to4850995) | 22.70% | 7379(5678to92 45) | 0.38(0.26to0.5) | 1490(626to309 9) | 22.90% | 3(1to6) | 0.38(0.26to0.5) |
| South Sudan | 2221552(1418 647to3058682) | 57.61% | 15533(9833to2 1372) | 0(-0.01to0.01) | 1000343(7477 74to1297244) | 56.58% | 6929(5200to89 55) | 0(-0.01to0.01) | 382(159to808) | 56.76% | 3(1to6) | 0.01(0to0.02) |
| Spain | 3147941(1855 063to4530520) | 120.27% | 13441(7912to1 9238) | 2.66(2.05to3.2 8) | 1364342(1009 671to1777701) | 10.76% | 5954(4390to77 80) | 1.03(0.8to1.27) | 525(216to1121 ) | 11.12% | 2(1to5) | 1.05(0.81to1.2 8) |
| Sri Lanka | 2875553(1855 441to3861512) | -10.58% | 16364(10822to 21870) | 0(0to0.01) | 1323143(1020 001to1629216) | -13.37% | 7785(6021to95 74) | 0.12(-0.02to0.26) | 509(223to1075 ) | -12.92% | 3(1to6) | 0.13(-0.01to0.27) |
| Sudan | 9471913(6451 678to12535031 | 97.95% | 17188(11705to 22739) | 0.11(-0.09to0.3) | 4162329(3204 444to5286174) | 67.40% | 7550(5814to95 85) | -0.67(-0.83to-0.51) | 1594(682to336 2) | 67.76% | 3(1to6) | -0.66(-0.81to- 0.5) |
| Suriname | 89083(60817to 118048) | 10.41% | 17346(11991to 22847) | 0(-0.02to0.02) | 40219(31128to 49110) | 6.54% | 8070(6249to97 97) | -0.05(-0.06to-0.04) | 15(7to33) | 6.57% | 3(1to7) | -0.05(-0.05to- 0.04) |
| Sweden | 1010619(6394 19to1368075) | 36.79% | 16028(10197to 21741) | 0.26(0.13to0.3 9) | 433013(32430 5to558133) | 52.64% | 6951(5176to90 04) | 0.82(0.46to1.1 7) | 167(70to349) | 52.40% | 3(1to6) | 0.82(0.47to1.1 8) |
| Switzerland | 609000(32363 3to910489) | 26.93% | 13265(7034to1 9808) | -0.36(-0.61to- 0.1) | 218922(14893 6to292024) | 32.40% | 4780(3250to63 80) | -0.74(-1.17to-0.32) | 84(34to172) | 32.68% | 2(1to4) | -0.74(-1.17to-0.31) |
| Syrian Arab Republic | 2236465(1451 536to3032306) | -40.76% | 17408(11450to 23227) | 0.07(0.02to0.1 2) | 876804(70059 5to1100249) | -42.89% | 7080(5647to88 84) | 0.04(0.02to0.0 6) | 336(143to708) | -42.99% | 3(1to6) | 0.05(0.03to0.0 7) |
| Taiwan (Province of China) | 1694909(1090 576to2264644) | -43.91% | 15909(10353to 21113) | -0.45(-0.62to-0.29) | 852630(66744 6to1039358) | -40.93% | 8122(6341to98 63) | 1.25(0.83to1.6 7) | 327(138to659) | -40.98% | 3(1to6) | 1.25(0.83to1.6 7) |
| Tajikistan | 2073771(1481 832to2653131) | 40.94% | 17908(12702to 23036) | 0.02(0.01to0.0 3) | 1013103(8089 33to1202942) | 37.26% | 8613(6889to10 218) | -0.01(-0.02to0) | 387(169to776) | 37.72% | 3(1to7) | 0(-0.01to0.01) |
| Thailand | 5943932(3890 936to8020845) | -34.59% | 16308(10889to 21760) | 0.19(0.13to0.2 6) | 2834571(2209 361to3474004) | -49.35% | 8100(6305to99 02) | -0.67(-0.72to-0.62) | 1084(461to216 5) | -49.36% | 3(1to6) | -0.66(-0.72to-0.61) |
| Timor-Leste | 283276(18883 2to366234) | 45.70% | 16310(10835to 21111) | 0(-0.01to0.01) | 139458(10934 3to170444) | 37.61% | 8021(6282to97 96) | -0.04(-0.05to-0.02) | 53(23to106) | 37.68% | 3(1to6) | -0.03(-0.04to-0.01) |
| Togo | 1810613(1147 667to2461537) | 76.08% | 16202(10267to 22035) | 0(-0.01to0) | 803480(57681 6to1032249) | 70.36% | 7173(5150to92 14) | -0.02(-0.04to0) | 307(128to635) | 70.92% | 3(1to6) | -0.02(-0.03to0) |
| Tokelau | 291(204to378) | -32.31% | 17436(12174to 22742) | 0.03(0.01to0.0 5) | 142(113to171) | -35.56% | 8348(6677to10 071) | -0.03(-0.05to-0.02) | 0(0to0) | -35.43% | 3(1to7) | -0.03(-0.05to-0.01) |
| Tonga | 21831(15451to 28375) | -9.96% | 17279(12247to 22454) | 0(-0.01to0) | 10570(8400to1 2706) | -8.87% | 8414(6690to10 102) | -0.01(-0.01to0) | 4(2to9) | -9.07% | 3(1to7) | 0(-0.01to0) |
| Trinidad and  Tobago | 150309(90418t o212487) | -40.33% | 15718(9542to2 1947) | -0.4(-0.47to-0.32) | 54165(42525to 68577) | -49.56% | 5850(4600to7405) | -1.18(-1.41to-0.95) | 21(9to44) | -49.39% | 2(1to5) | -1.18(-1.4to- 0.95) |
| Tunisia | 1602746(1094 284to2117169) | -17.17% | 17211(11825to 22523) | 0(-0.01to0.02) | 728319(57226 8to899978) | -19.44% | 7924(6196to9806) | -0.03(-0.04to-0.03) | 281(120to584) | -19.20% | 3(1to6) | -0.03(-0.03to-0.02) |
| Turkey | 9137064(6333 581to12098461 | -28.35% | 17190(11915to 22510) | 0(-0.04to0.05) | 4086896(3143 791to5082540) | -30.84% | 7866(6014to9756) | 0.09(-0.04to0.23) | 1573(669to331 8) | -30.58% | 3(1to6) | 0.11(-0.03to0.25) |
| Turkmenistan | 979251(68738 6to1270845) | 2.88% | 17984(12632to 23411) | 0.04(0.03to0.0 6) | 464386(37059 7to554197) | 0.15% | 8454(6753to10 104) | -0.04(-0.05to-0.03) | 178(76to362) | 0.25% | 3(1to7) | -0.04(-0.05to-0.02) |
| Tuvalu | 2051(1420to27 02) | 1.05% | 17399(12138to 22865) | 0.01(-0.01to0.02) | 970(777to1166 ) | -6.75% | 8433(6770to10 134) | 0(-0.01to0) | 0(0to1) | -6.73% | 3(1to7) | 0(-0.01to0.01) |
| Uganda | 10801165(680 9390to1465337 2) | 127.06% | 16113(10158to 21976) | 0.26(-0.26to0.78) | 4668972(3452 060to6076933) | 120.03% | 6865(5091to8908) | 0.02(-0.24to0.28) | 1786(770to379 8) | 121.32% | 3(1to6) | 0.05(-0.21to0.31) |
| Ukraine | 4489502(3164 467to5906699) | -38.67% | 18325(13141to 24036) | -0.02(-0.03to-0.01) | 1996488(1614 746to2371428) | -40.26% | 8420(6810to99 53) | 0(-0.01to0.01) | 768(333to1596 ) | -40.24% | 3(1to7) | 0.01(-0.01to0.02) |
| United Arab  Emirates | 743918(52907 1to945804) | 87.84% | 17542(12734to 22086) | 0.03(-0.02to0.08) | 374188(32644 4to424676) | 98.97% | 9110(7908to10 352) | 0.3(0.14to0.45) | 144(63to285) | 99.55% | 4(2to7) | 0.3(0.15to0.45) |
| United  Kingdom | 3382516(1844 682to5373561) | -39.97% | 8178(4477to12 827) | -2.78(-3.25to-2.3) | 1432228(1032 545to1917786) | -34.98% | 3503(2535to4693) | -2.63(-3.16to-2.11) | 551(234to1174 ) | -34.96% | 1(1to3) | -2.63(-3.15to- 2.1) |
| United  Republic  Of Tanzania | 10141545(526 5794to14989705) | 115.45% | 11834(6102to1 7623) | -0.88(-1.28to-0.48) | 3613512(2495 127to4946218) | 42.56% | 4144(2860to5711) | -2.26(-2.68to-1.83) | 1388(563to3203) | 44.21% | 2(1to4) | -2.22(-2.64to-1.79) |
| United States of America | 35650223(235 11379to473016 22) | 7.36% | 17438(11695to 23108) | 0.08(0.01to0.1 4) | 15381525(120 25700to189383 40) | 4.16% | 7681(5998to9460) | 0.07(-0.21to0.35) | 5915(2555to12 588) | 4.10% | 3(1to6) | 0.07(-0.21to0.35) |
| United States Virgin Islands | 12068(7985to1 6162) | -35.91% | 17493(11736to 23358) | 0.01(0to0.03) | 5389(4239to65 07) | -38.67% | 7970(6223to9630) | -0.03(-0.04to0.01) | 2(1to4) | -38.62% | 3(1to6) | -0.03(-0.04to-0.01) |
| Uruguay | 427432(29394 7to566985) | -11.46% | 17600(12208to 23252) | 0.02(-0.01to0.05) | 184865(14413 7to230303) | -12.60% | 7705(5986to9610) | -0.07(-0.14to0.01) | 71(30to149) | -12.45% | 3(1to6) | -0.07(-0.13to-0.01) |
| Uzbekistan | 6605533(4677 219to8531827) | 20.96% | 17929(12701to 23253) | 0(-0.01to0.01) | 3151926(2502 451to3747440) | 16.10% | 8547(6787to10 163) | 0.01(0to0.02) | 1202(530to241 9) | 16.39% | 3(1to7) | 0.01(0to0.02) |
| Vanuatu | 69178(49182to 89747) | 67.61% | 17496(12466to 22673) | 0.01(0to0.02) | 33006(26063to 39751) | 59.87% | 8427(6659to10 144) | -0.01(-0.02to0.01) | 13(6to25) | 60.41% | 3(1to6) | -0.01(-0.02to0) |
| Venezuela  (Bolivarian  Republic of) | 3969459(2519 922to5352853) | -3.25% | 16407(10519to 21972) | -0.02(-0.03to0.01) | 1757942(1355 344to2197046) | -3.59% | 7374(5668to92 29) | -0.03(-0.05to0.02) | 675(290to1388 ) | -3.56% | 3(1to6) | -0.03(-0.05to-0.01) |
| Viet Nam | 12507437(828 9946to16523831) | -17.49% | 16730(11219to 22037) | 0.13(0.1to0.16) | 6165684(4990 826to7430116) | -19.40% | 8378(6756to10 078) | 0.09(0.04to0.1 5) | 2365(996to485 6) | -19.11% | 3(1to7) | 0.1(0.04to0.16) |
| Yemen | 7921778(5443 213to10349289 | 79.06% | 17331(11934to 22661) | 0.02(0.01to0.0 3) | 3676579(2850 414to4504447) | 71.00% | 8082(6255to99 06) | -0.02(-0.04to0.01) | 1401(596to294 4) | 70.81% | 3(1to6) | -0.03(-0.04to-0.02) |
| Zambia | 4287399(2645 794to5813998) | 111.10% | 15719(9693to2 1345) | -0.02(-0.03to0.01) | 1953684(1437 380to2493125) | 105.62% | 7090(5229to90 31) | -0.04(-0.06to0.01) | 746(316to1553 ) | 105.79% | 3(1to6) | -0.03(-0.05to0) |
| Zimbabwe | 3209569(1884 261to4490103) | 48.38% | 15256(8950to2 1353) | 0.85(0.69to1.0 2) | 1272852(9678 96to1717828) | 62.29% | 6033(4589to81 38) | 1.4(1.15to1.65) | 489(203to1071 ) | 62.87% | 2(1to5) | 1.41(1.16to1.6 6) |
